# Electrochemical Synthesis of Building Blocks with a Structure Inspired by Nonsteroidal Anti-Inflammatory Drugs

**DOI:** 10.1021/acsomega.6c07418

**Published:** 2026-08-31

**Authors:** Jakub Adamek, Agnieszka Październiok-Holewa

**Affiliations:** † 49569 Silesian University of Technology, Department of Organic Chemistry, Bioorganic Chemistry and Biotechnology, B. Krzywoustego 4, Gliwice 44-100, Poland; ‡ Silesian University of Technology, Biotechnology Center, B. Krzywoustego 8, Gliwice 44-100, Poland

## Abstract

A mild, metal- and additive-free, inexpensive electrochemical decarboxylation of arylalkanecarboxylic acids, including all the most important nonsteroidal anti-inflammatory drugs based on such a framework, is discussed. An efficient, nonaqueous, and essentially no-chromatography workup procedure enhances the attractiveness of the disclosed solution. This methodology can facilitate scaffold diversification by functionalization of NSAIDs or by providing easy access to building blocks from a simple arylalkanecarboxylic acid feedstock.

## Graphical abstract

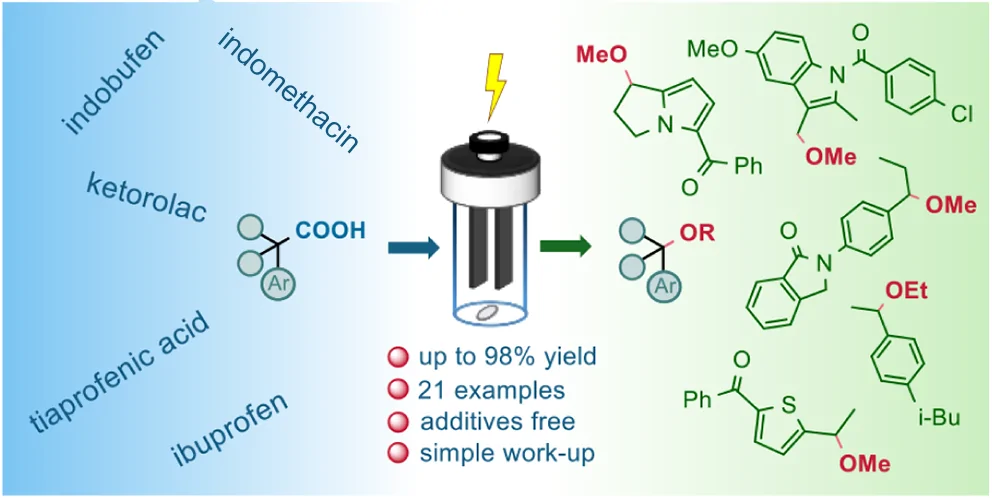

## 1 Introduction

Nonsteroidal anti-inflammatory drugs are a structurally diverse group of compounds exhibiting extremely valuable, desirable, and crucial properties, such as anti-inflammatory, antipyretic, and analgesic effects.^1–4^ Their mechanism of action is based on the inhibition of prostaglandin cyclooxygenase (COX) activity.^5,6^ Among all NSAIDs, aromatic compounds derived from propionic acid (profens, e.g., ibuprofen, zaltoprofen, naproxen) and acetic acid (e.g., indomethacin, diclofenac) constitute a significant group. Furthermore, the direct proximity of the aryl and carboxyl moieties (attached to the same carbon atom) means that they should undergo electrochemical decarboxylation relatively easily.^7^ Decarboxylation induced by an electric current has been reported in the literature for years. Depending on the substrate structure and reaction conditions, the process can proceed via a radical mechanism, leading to the formation of dimers (the Kolbe reaction), or via cationic intermediates, which then react with nucleophiles to give a diverse range of products (the so-called non-Kolbe reaction).^7–12^

Despite the wide range of applications of electrochemical decarboxylation methods, there are relatively few reports in the literature regarding their use for the structural modification of nonsteroidal anti-inflammatory drugs. Initial studies on the influence of substrate structure on the methoxylation/oxidation of 1-arylalkanecarboxylates, using phenylacetic acid derivatives as an example, were already presented in 1971 by Coleman et al.^13^ However, only in 2024, Li et al. described electrochemical decarboxylative cross-coupling with nucleophiles (including methanol) of a few compounds whose structures were inspired by nonsteroidal anti-inflammatory drugs. The reactions were carried out in a complex electrolytic system containing: substrate (e.g., NSAID); solvent: methylene chloride or a mixture of tetrahydrofuran (THF) and 1,1,1,3,3,3-hexafluoro-2-propanol (HFIP); Na_2_HPO_4_; electrolyte (e.g., Et_4_NBF_4_ or *n*-Bu_4_NPF_6_); and the appropriate nucleophile: methanol, Et_3_N·3HF, water, or potassium acetate. Electrolysis was conducted under an argon atmosphere, in an undivided cell, using a graphite anode and a nickel or platinum cathode. Particularly, methoxylations of ibuprofen and loxoprofen were carried out at a current of 10 mA for 90 min (2.8 F/mol) with yields of 40 and 41% (Scheme 1A).^14^

**Scheme 1:**
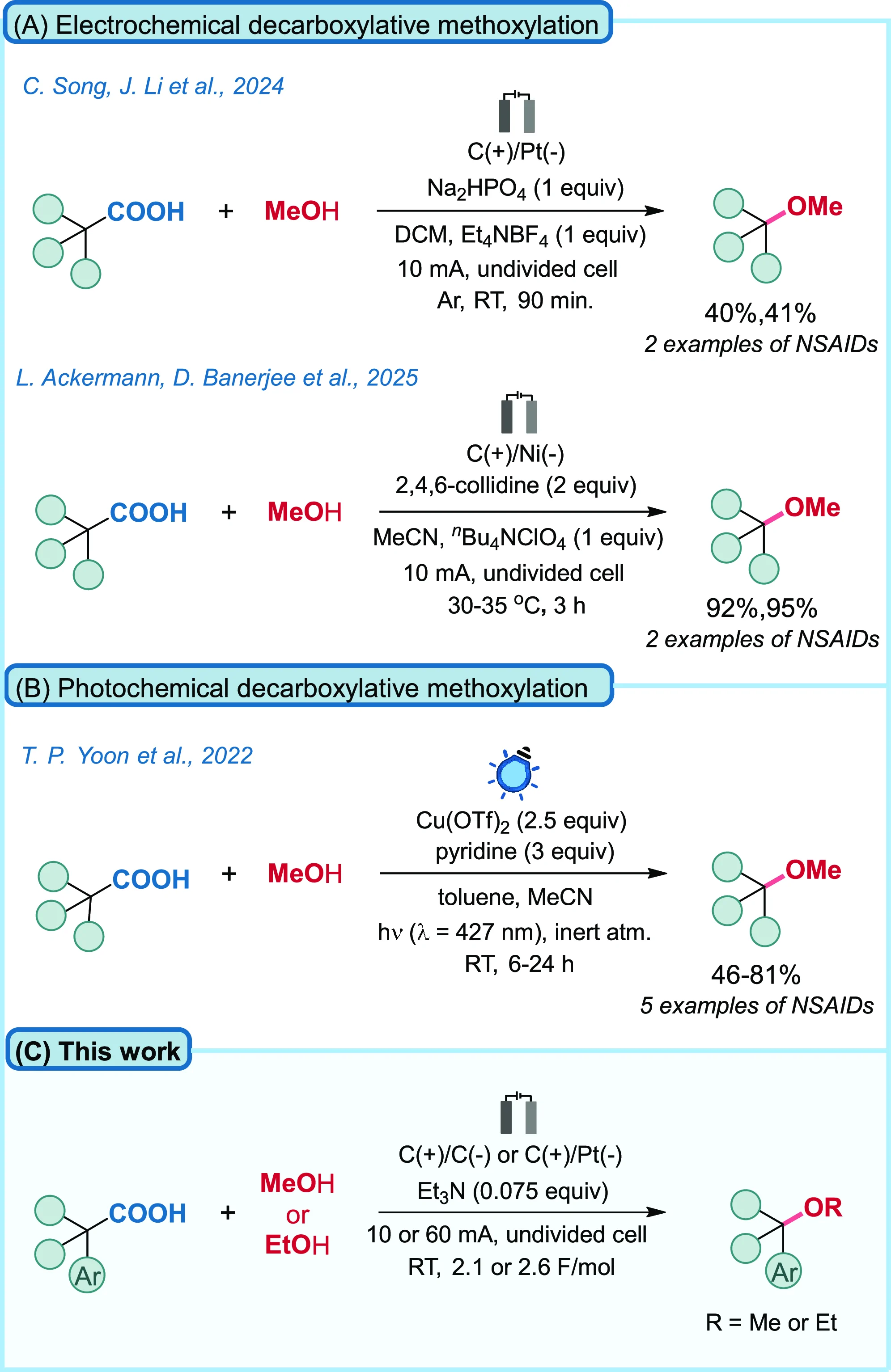
Strategies for the Decarboxylative Alkoxylation of Nonsteroidal Anti-Inflammatory Drugs (NSAIDs)

A year later, the electrochemical decarboxylative methoxylation of ibuprofen and naproxen was discussed by Banerjee and coworkers.^15^ It was carried out at room temperature, at a current of 10 mA for 3 h (3.75 F/mol), in an electrolyzer of similar design (undivided electrolytic cell, graphite anode, and nickel cathode). The reaction mixture contained the substrate (ibuprofen or naproxen), acetonitrile as a solvent, 2,4,6-collidine, *n*-Bu_4_NClO_4_, and methanol. The methoxy derivatives were obtained in high yields of 92% (ibuprofen) and 95% (naproxen), respectively (Scheme 1A).^15^ Under similar conditions, with a modified stoichiometric ratio of the reagents, decarboxylative alkoxylation of ibuprofen was also performed in the presence of 1-phenylethanol, affording the product in 95% yield.^15^

Decarboxylation of such compounds can also be conducted using nonelectrochemical methods. The most interesting one is the photochemical procedure, based on the action of electromagnetic radiation at a wavelength of 427 nm, generated by a 40 W blue LED lamp. Decarboxylative methoxylation of substrates (0.3 mmol; ibuprofen, flurbiprofen, loxoprofen, ketoprofen, and naproxen) was carried out in a mixture of toluene and acetonitrile (19:1, v/v), in the presence of pyridine (0.9 mmol, 3 equiv), Cu­(OTf)_2_ (0.75 mmol, 2.5 equiv), and methanol (0.3–0.9 mmol), at room temperature, for 6 to 24 h, with yields ranging from 46% to 81% (Scheme 1B).^16^

All in all, each of the described methods uses numerous additional troublesome substances (electrolytes, such as perchlorates and ammonium salts; bases, such as pyridine, 2,4,6-collidine, etc.). As a result, the isolation of the product requires an aqueous workup and chromatographic purification, which is a significant disadvantage of these protocols. Moreover, because of the lack of a comprehensive analysis, there is a gap in the research on possible structural modifications of NSAIDs using decarboxylative methoxylation. It seems justified to continue searching for a simple method to transform the carboxyl group, which can be used for the functionalization of NSAIDs, and to increase the availability of building blocks based on 1-alkoxy-1-arylalkane scaffolds inspired by NSAIDs.^17,18^

## 2 Results and Discussion

We began our research by selecting conditions for a model reaction of electrochemical decarboxylative methoxylation of ibuprofen (see Table 1). Each experiment was performed in an undivided electrolyzer using the ElectraSyn 2.0 setup equipped with a glass vial (5 or 20 mL) and a magnetic stirrer. We decided to check whether the reaction takes place in a simple three-component system: substrate/methanol/base, without any other additives. Considering the use of different bases, we chose triethylamine, which can be easily evaporated together with methanol after the reaction completion.

**Table 1:**
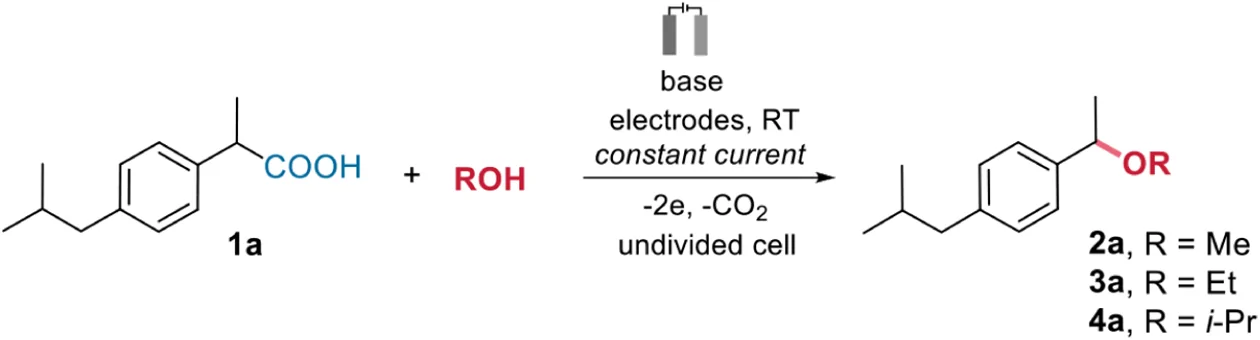
Optimization of the Reaction Conditions^a^

Entry	ROH	Electrodes	Base (% mol)	I [mA]	Q [F mol^–1^]	Time [min]	Product	Conv. [%]^b^	**Yield** ^c^ [%]
1	MeOH	C(+)/Pt(−)	Et_3_N (7.5)	10	2.1	135	**2a**	>99	96
2	MeOH	C(+)/Pt(−)	Et_3_N (7.5)	60	2.1	22.5	**2a**	>99	93
3	MeOH	C(+)/C(−)	Et_3_N (7.5)	60	2.1	22.5	**2a**	>99	95
4^d^	MeOH	C(+)/C(−)	Et_3_N (7.5)	60	2.1	90	**2a**	>99	90
5^e^	MeOH	C(+)/C(−)	Et_3_N (7.5)	60	2.1	360	**2a**	>99	92
6	MeOH	C(+)/C(−)	SiO_2_–Pip (22.5)	8	2.1	169	**2a**	>78	^f^
7	MeOH	C(+)/C(−)	SiO_2_–Pip (30.0)	8	3.0	241	**2a**	>99	90
8	EtOH	C(+)/C(−)	Et_3_N (7.5)	10	2.1	135	**3a**	>80	^f^
9	EtOH	C(+)/C(−)	Et_3_N (7.5)	10	2.6	167	**3a**	>99	95
10	*i*-PrOH	C(+)/C(−)	Et_3_N (7.5)	1	2.1	1350	**4a**	^g^	^g^

a Conditions: substrate **1a** (0.4 mmol), base, ROH (4 mL), room temperature. Entries 1–4 and 6–10 were performed in a 5 mL vial using an IKA ElectraSyn 2.0.

b Determined by integration of signals in the ^1^H NMR spectrum.

c Isolated yield.

d The reaction was carried out on a scale of 1.6 mmol of **1a**, MeOH (4 mL) in a 5 mL vial.

e The reaction was carried out on a scale of 6.4 mmol (1.32 g) of **1a**, MeOH (16 mL) in a 20 mL vial (see SI).

f Due to the incomplete conversion of substrate **1a**, the product was not isolated.

g Due to the impractically long reaction time and unstable electrolysis conditions (for the maximum current achieved of 1 mA, the voltage was 30 V—the maximum for ElectraSyn 2.0), the reaction with 2-propanol was discontinued.

Initially, the current conditions (constant current of 10 mA, electrode material: C­(+)/Pt(−)) were proposed, taking into account the protocol described in the literature.^14,15^ The reaction proceeded smoothly, but the full conversion of 0.4 mmol of substrate took over 2 h (entry 1, Table 1). Therefore, we decided to increase the current to 60 mA (entry 2, Table 1), resulting in a 6-fold reduction of the electrolysis time, which is very important from a practical point of view. The use of the cheapest available graphite electrodes also turned out to be a good move, leading to a slightly higher yield of 95% (entry 3, Table 1).

Next, we tested the possibility of using solid-supported piperidine (SiO_2_–Pip) as a base to simplify the workup procedure and make it more “green” (SiO_2_–Pip could be a reusable catalyst^19,20^ ). Nevertheless, this modification resulted in a decrease in solution conductivity, a decrease in current efficiency (3.0 F/mol of the charge is required; see entries 6 and 7, Table 1), and also mechanical damage to the electrodes.

Finally, having selected conditions in hand (C­(+)/C(−), 60 mA, 2.1 F/mol), the electrochemical decarboxylation was successfully scaled up from 0.4 to 6.4 mmol, i.e., over the gram scale (entries 4–5, Table 1; see SI).

Moreover, we verified the possibility of using other simple alcohols (ethanol or isopropanol) as solvents and nucleophiles in electrochemical decarboxylation. We observed that the conductivity of the reaction mixture consisting of EtOH, Et_3_N, and an NSAID was significantly lower. Furthermore, the current efficiency also decreased, and 2.6 F/mol of charge was required to achieve full substrate conversion (entries 8 and 9, Table 1). However, we were still able to obtain ethoxylation products in high yields (entry 9, Table 1). On the contrary, achieving sufficient conductivity in 2-propanol solutions proved to be even more challenging. Although increasing the amount of Et_3_N (0.075 equiv to 1.0 equiv) led to some improvement, the long reaction time was still very impractical; for a current of 1 mA (the voltage was 30 V—the maximum for ElectraSyn 2.0), and the reaction time was 22.5 h. For this reason, we did not conduct further studies with 2-propanol (entry 10, Table 1).

Interesting results of our preliminary studies encouraged us to verify the method on a wide group of anti-inflammatory drugs and on several simple 1-arylalkanecarboxylic acids. To the best of our knowledge, this type of research has not been conducted to this extent so far. Naproxen, loxoprofen, flurbiprofen, fenoprofen, and 1-arylalkanecarboxylic acids react efficiently under optimized conditions with yields ranging from 85% to 98% (compounds **2b**–**2j**, Scheme 2, Procedure A). However, in the case of ketoprofen or compounds containing a nitrogen or sulfur atom (e.g., indobufen, ketorolac, pranoprofen, zaltoprofen, or tiaprofenic acid), the platinum-coated cathode and constant current of 10 mA proved to be more suitable (compounds **2k**–**2p**, Scheme 2, Procedure B). Moreover, the lower solubility of pranoprofen required the use of a larger amount of Et_3_N (22.5 mol%, see compound **2m**, Scheme 2, Procedure B). Generally, the workup procedure is quite simple and involves only the removal of volatile components, but a certain difficulty is the relatively high volatility of some of the obtained compounds (especially derivatives of simple 1-arylalkanecarboxylic acids, such as **2g** or **2h**). Only in a few cases was it necessary to further purify the products by column chromatography (compounds **2o** and **2p** from ketoprofen and zaltoprofen; see SI).

**Scheme 2:**
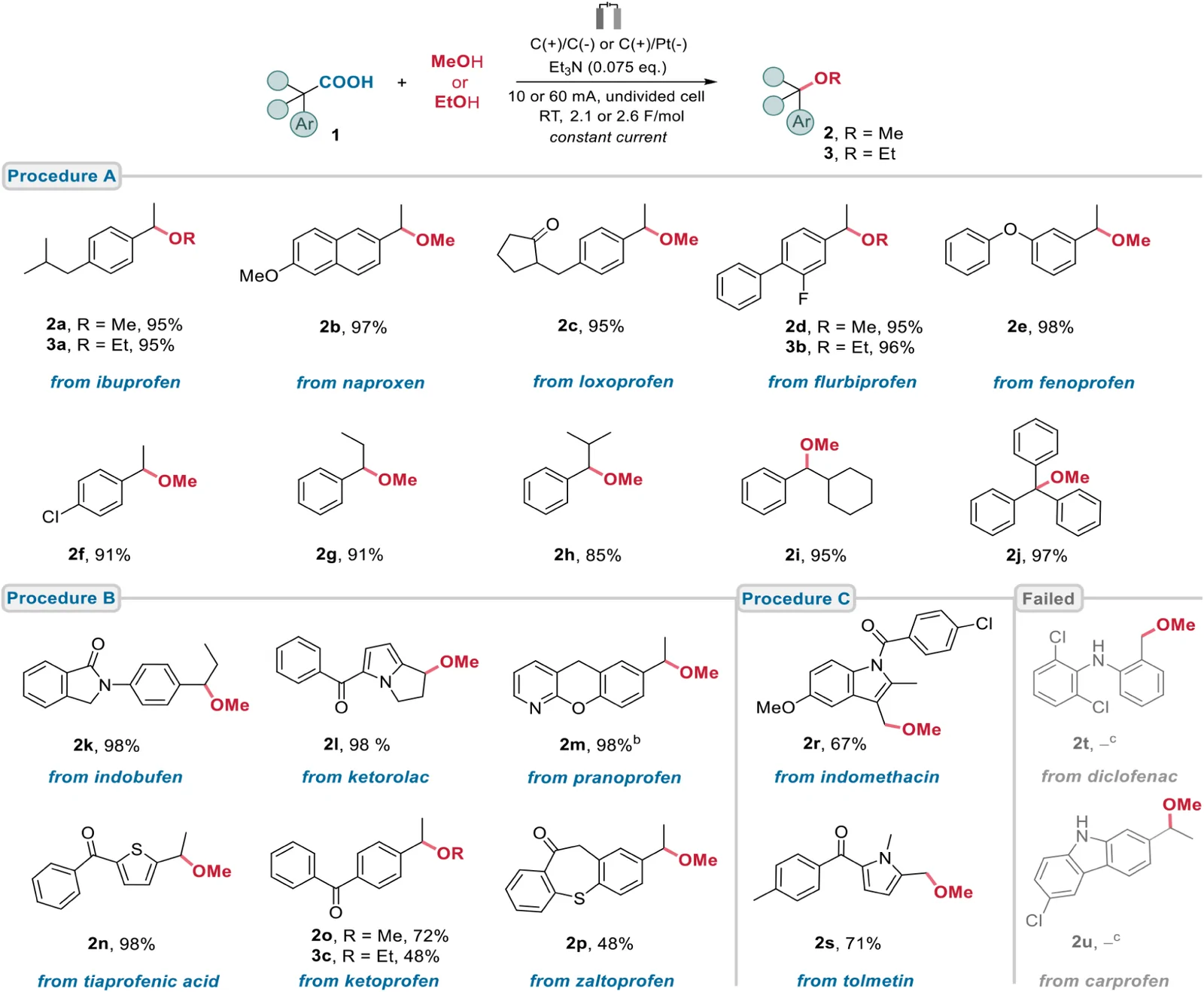
Substrate Scope for Decarboxylative Alkoxylation of Nonsteroidal Anti-Inflammatory Drugs and Simple 1-Arylalkanecarboxylic Acids^a^ ^b^ ^c^

a Reaction conditions: Procedure A: graphite SK-50 plate as anode and cathode, constant current = 60 mA, charge 2.1 or 2.6 F/mol, substrate **1** (0.4 mmol), Et_3_N (7.5 mol%), MeOH or EtOH (4 mL) in a 5 mL vial, at room temperature, yield of isolated product. Procedure B: graphite SK-50 plate as anode and Pt plate as cathode, constant current = 10 mA, charge 2.1 F/mol, substrate **1** (0.4 mmol), Et_3_N (7.5 mol%), MeOH or EtOH (4 mL) in a 5 mL vial, at room temperature, yield of isolated product.
b The reaction was performed with substrate **1m** (0.4 mmol) and Et_3_N (22.5 mol%) in MeOH (4 mL) in a 5 mL vial. Procedure C: graphite SK-50 plate as anode and Pt plate as cathode, constant current = 10 mA, charge 2.1 F/mol, **1r** or **1s** (0.4 mmol), Et_3_N (0.4 mmol) in MeOH (16 mL) in a 20 mL vial at room temperature, yield of isolated product.
c Complex reaction mixture not containing the expected product (procedures A–C failed).

These observations were also confirmed for decarboxylative ethoxylation reactions that were carried out at a current of 10 mA on ibuprofen (2.6 F/mol of the charge is required), flurbiprofen (2.6 F/mol of the charge is required), and ketoprofen (2.1 F/mol of the charge is required), with yields of 95% (**3a**), 96% (**3b**), and 48% (**3c**), respectively.

However, the most challenging was the decarboxylation of nonsteroidal anti-inflammatory drugs derived from acetic acid, such as indomethacin and tolmetin (compounds **2r** and **2s**, Scheme 2, Procedure C). Both procedures A and B led to the formation of a complex reaction mixture in which we observed (^1^H NMR) the expected products; however, with unsatisfactory yields and conversions. To avoid the formation of byproducts (according to literature reports, we expected, e.g., Kolbe dimerization^21,22^ ), we use a lower substrate concentration, which should hinder the recombination of radicals formed during the Kolbe reaction. It turned out to be a good solution; nevertheless, chromatography was necessary to purify the main reaction products (see the SI). Unfortunately, we have not yet been able to select electrolysis conditions for compounds containing a free amino group, such as diclofenac and carprofen (compounds **2t** and **2u**, Scheme 2, Failed). As a result of electrolysis carried out under all tested conditions, reaction mixtures were obtained in which we did not observe (NMR) the presence of the expected products.

## 3 Conclusions

We have developed and described an efficient, metal- and additive-free method for the inexpensive electrochemical decarboxylation of 1-arylalkanecarboxylic acids, including the most important nonsteroidal anti-inflammatory drugs. The advantages of the discussed protocols are good to very good reaction yields, high selectivity, and mild process conditions. The procedure is characterized by short reaction times and high current efficiency (only 2.1 F/mol charge is needed for methoxylation). It relies on readily available substrates such as nonsteroidal anti-inflammatory drugs, enabling the production of a wide library of compounds with diverse structures. The fact that the method does not require the use of additional reagents significantly simplifies the product-isolation procedure. In general, evaporation of the volatile substances (solvent and triethylamine) is sufficient. Therefore, in most cases, product purification does not require chromatography. The process is easily scalable (it was tested in the range from 0.4 to 6.4 mmol of substrate; gram scale is available) and can be used both for obtaining final alkoxylated products (late-stage functionalization) and as a stage for modifying the molecular skeleton (scaffold diversification) in more complex synthetic pathways.

## Supplementary Material



## Data Availability

The data underlying this study are available in the published article and its Supporting Information.

## Notes

Experimental procedures, compound characterization data, and ^1^H and ^13^C NMR spectra (PDF).
