# Supplementary material for: Electrochemical Synthesis of Building Blocks with a Structure Inspired by Nonsteroidal Anti-Inflammatory Drugs
Source: ACS Omega. 2026 Aug 31;11(36):54650–4. doi: 10.1021/acsomega.6c07418 (PMC13584791; doi:10.1021/acsomega.6c07418)
Supplement: Supplementary file 1 [file ao6c07418_si_001.pdf]

Supplementary Information

Electrochemical synthesis of building blocks with a structure inspired by nonsteroidal anti-inflammatory drugs

Jakub Adamek* [a,b] and Agnieszka Październiok-Holewa[a,b]

[a] Silesian University of Technology, Department of Organic Chemistry and Bioorganic Chemistry and Biotechnology, Krzywoustego 4, 44-100 Gliwice, Poland

[b] Silesian University of Technology, Biotechnology Center, Krzywoustego 8, 44-100 Gliwice, Poland

*****Correspondence: jakub.adamek@polsl.pl; Tel.: +48 032-237-1724; fax: +48 032-237-2094

Table of contents:

1. General information……………………………………………………………………………S2

2. General experimental procedures for the synthesis of 2a-s and 3a-c…………………………S2

3. Characterization data of products 2a-s and 3a-c………………………………………………S5

4. ^{1}H and ^{13}C NMR spectra of products …………………………………………………S10

5. ^{1}H NMR spectra of the selected reaction mixtures…………………………………………S31

6. References ……………………………………………………………………………………S35

1. General information

All reagents and solvents were commercially available and used as received without further purification. Reaction of electrochemical decarboxylative methoxylation were carried out using the commercially available Electrasyn 2.0 kit from IKA® with no precautions to exclude air or moisture.

Kieselgel 60 (Merck, 0.040-0.063 mm) was used for column chromatography. For TLC analysis, Merck TLC silica gel 60 F_{254} plates were used. The plates were visualized by UV light (254 nm) and dipped in a solution of cerium sulfate and tetrahydrate of ammonium heptamolybdate in H_2SO_{4aq} and heated.

^{1}H NMR spectra were acquired on an Agilent Magnet 400 MHz spectrometer. Data were recorded as follows: chemical shift in ppm with tetramethylsilane (TMS) as the internal standard, multiplicity (s = singlet; d = doublet; t = triplet; q = quartet; oct = octet, br = broad; m = multiplet), coupling constant (Hz), integration, and assignment. ^{13}C NMR spectra were measured an Agilent Magnet 400 at 101 MHz. Chemical shifts were reported in ppm from the solvent resonance employed as the internal standard ($CDCl_3$ at 77.16 ppm). Infrared spectra (IR) were recorded on a Nicolet 6700 FT-IR spectrophotometer (ATR method). Melting points were determined in capillaries, in a Stuart Scientific SMP3 melting point apparatus and were uncorrected. The high-resolution mass spectra (HRMS) were obtained by electrospray ionization (ESI), using a Waters Corporation Xevo G2 QTOF instrument with time-of-flight (TOF) analyzer.

2. General experimental procedures for the synthesis of 2a-s and 3a-c

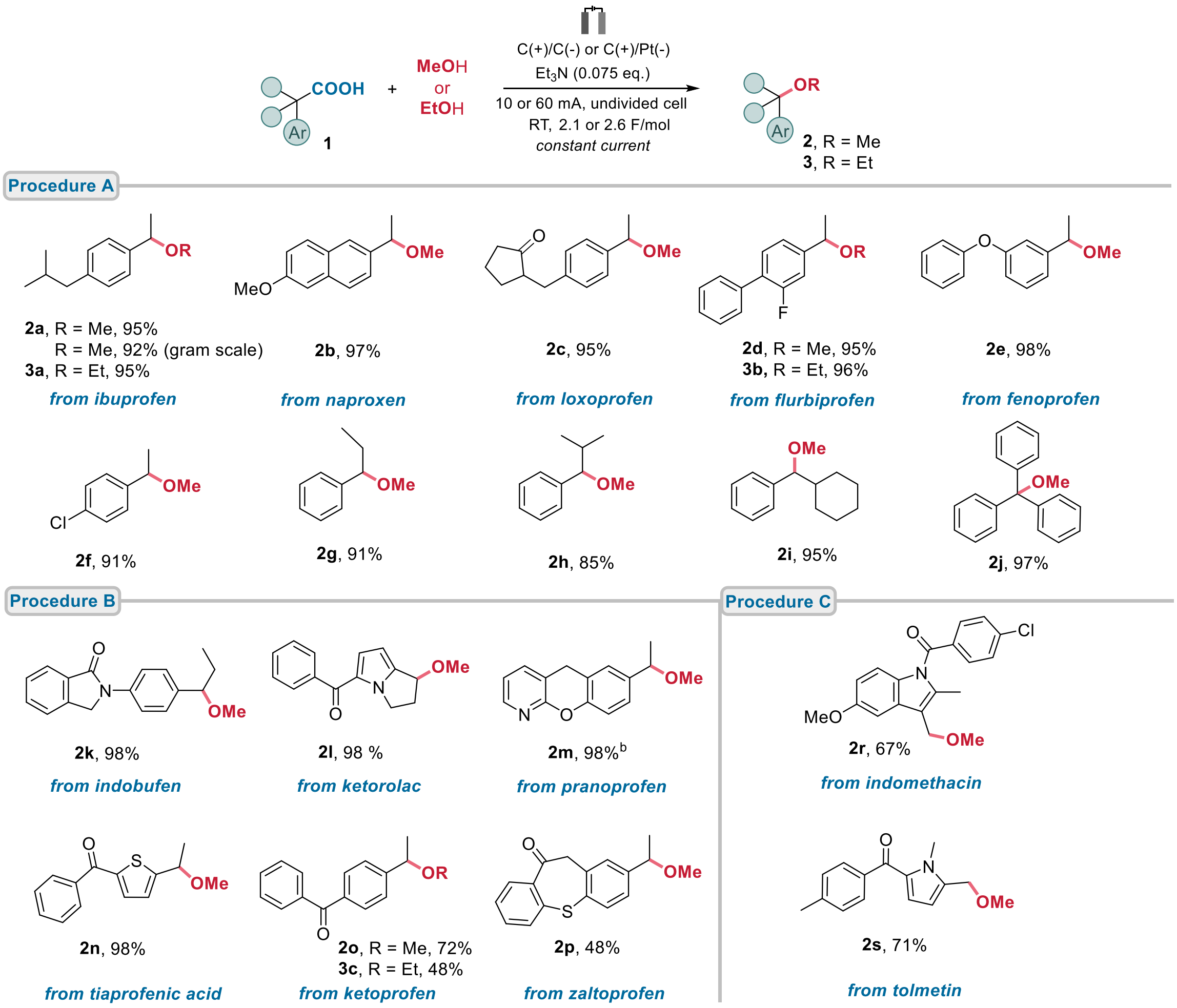

Scheme S1. Decarboxylative alkoxylation of nonsteroidal anti-inflammatory drugs and simple 1-arylalknecarboxylic acids.

General Procedure A for the synthesis of compounds 2a-2j and 3a,b

ElectraSyn 2.0 vial (5mL), equipped with a magnetic stir bar, was charged sequentially with carboxylic acid **1a-j** (0.4 mmol, 1 eq.), MeOH or EtOH (4 mL), and Et_3N (4.2 µL, 0.03 mmol, 0.075 eq.) for **1a-d**, **f-j** or (8.4 µL, 0.06 mmol, 0.15 eq.) for **1e**. Then the vial cap equipped with two Graphite SK-50 electrodes was inserted into the mixture, and the mixture was stirred at 500 rpm for 4 minutes. Next, the vial was put into the ElectraSyn 2.0 setup, and electrolysis was carried out at room temperature. The mixture was stirred at 900 rpm in constant-current mode (60 mA) until 2.1 F/mol of charge was passed (time: 22 min 29 sec) for reaction in MeOH or in constant-current mode (10 mA) until 2.6 F/mol of charge was passed (time: 167 min) for reaction in EtOH. After completion of the reaction, the reaction mixture was quantitatively transferred to a round-bottom flask, and the electrodes were rinsed with methanol or ethanol (2 x 4 mL), which was combined with the mixture. The volatiles were removed using a rotary evaporator. Finally, the residue was carefully dried under reduced pressure (10-15mmHg) to yield the desired product **2a-j** and **3a,b**.

General Procedure B for the synthesis of compounds 2k-2p and 3c

ElectraSyn 2.0 vial (5mL), equipped with a magnetic stir bar, was charged sequentially with carboxylic acid **1k-p** (0.4 mmol, 1 eq.), MeOH or EtOH (4 mL), and Et_3N (4.2 µL, 0.03 mmol, 0.075 eq.) for **1k,l,n-p** or Et_3N (12.6 µL, 0.09 mmol, 0.225 eq.) for **1m**. Then the vial cap equipped with Graphite SK-50 anode and a platinum-coated cathode was inserted into the mixture, and the mixture was stirred at 500 rpm for 4 minutes. Next, the vial was put into the ElectraSyn 2.0 setup. The mixture was stirred at 900 rpm and electrolyzed at room temperature with a constant current of 10 mA until 2.1 F/mol of charge was passed (time: 135 min). After completion of the reaction, the reaction mixture was quantitatively transferred to a round-bottom flask, and the electrodes were rinsed with methanol or ethanol (2 x 4 mL), which was combined with the mixture. The volatiles were removed using a rotary evaporator. Finally, the residue was carefully dried under reduced pressure (10-15mmHg) to yield the desired product **2k-n**. In the case of products **2o** , **2p** and **3c**, the residues were further purified by column chromatography using a hexane/ethyl acetate as eluent system to obtain the pure products.

General Procedure C for the synthesis of compounds 2r and 2s

ElectraSyn 2.0 vial (20 mL), equipped with a magnetic stir bar, was charged sequentially with carboxylic acid **2r** or **2s** (0.4 mmol, 1 eq.), MeOH (16 mL), and Et_3N (56 µL, 0.4 mmol, 1 eq.). Then the vial cap equipped with Graphite SK-50 anode and a platinum-coated cathode was inserted into the mixture, and the mixture was stirred at 500 rpm for 4 minutes. Next, the vial was put into the ElectraSyn 2.0 setup. The mixture was stirred at 900 rpm and electrolyzed at room temperature with a constant current of 10 mA until 2.1 F/mol of charge was passed (time: 135 min). After completion of the reaction, the reaction mixture was quantitatively transferred to a round-bottom flask, and the electrodes were rinsed with methanol (2 x 10 mL), which was combined with the mixture. The volatiles were removed under reduced pressure and obtained residue was further purified by column chromatography using dichloromethane or hexane/ethyl acetate as eluent system to obtain the pure product.

Procedure for the synthesis of 1-isobutyl-4-(1-methoxyethyl)benzene [*Ibuprofen derivative*] (2a) in a gram scale

To a ElectraSyn 2.0 vial (20 mL), equipped with a magnetic stir bar, ibuprofen (1.32 g, 6.4 mmol, 1 eq.), MeOH (16 mL), and Et_3N (67.2 µL, 0,48 mmol, 0,075 eq.) were added. Then the vial cap equipped with Graphite SK-50 anode and a platinum-coated cathode was inserted into the mixture, and the mixture was stirred at 500 rpm for 4 minutes. Next, the vial was put into the ElectraSyn 2.0 setup. The mixture was stirred at 900 rpm and electrolyzed at room temperature with a constant current of 60 mA until 2.1 F/mol of charge was passed (time: 6 hours). After completion of the reaction, the reaction mixture was quantitatively transferred to a round-bottom flask, and the electrodes were rinsed with methanol (2 x 15 mL), which was combined with the mixture. Next, the volatiles were removed using a rotary evaporator. Finally, the residue was carefully dried under reduced pressure (10-15 mmHg) to obtain 1.13 g of product **2a** as a slightly yellowish, transparent liquid in 92% yield.

3. Characterization data of products 2a-s

1-isobutyl-4-(1-methoxyethyl)benzene [*Ibuprofen derivative*] (2a):[1]

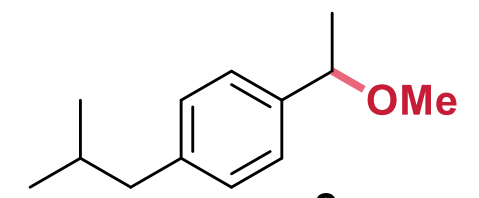

2a

Following the general procedure **A**, the title compound was isolated as a slightly yellowish, transparent liquid with 95% yield (73.1 mg). ^{1}H NMR (400 MHz, $CDCl_3$) δ 7.22-7.19 (m, 2H), 7.13-7.10 (m, 2H), 4.27 (q, *J* = 6.5 Hz, 1H), 3.21 (s, 3H), 2.46 (d, *J* = 7.2 Hz, 2H), 1.93-1.79 (m, 1H), 1.43 (d, *J* = 6.5 Hz, 3H), 0.90 (d, *J* = 6.6 Hz, 6H). ^{13}C{^{1}H} NMR (101 MHz, $CDCl_3$) δ 141.04, 140.80, 129.26, 126.11, 79.60, 56.46, 45.28, 30.36, 23.86, 22.53. IR (ATR) 3048, 2954, 1512, 1465, 1367, 1109, 1091, 845, 798 cm^{-1}.

2-methoxy-6-(1-methoxyethyl)naphthalene [*Naproxen derivative*] (2b):[1]

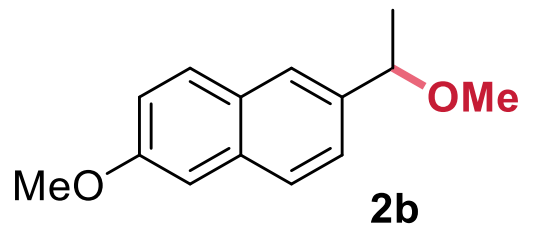

2b

Following the general procedure **A**, the title compound was isolated as a beige wax with 97% yield (83.9 mg). ^{1}H NMR (400 MHz, $CDCl_3$) δ 7.75-7.70 (m, 2H), 7.65 (br s, 1H), 7.42 (dd, *J* = 8.5, 1.8 Hz, 1H), 7.16 (d, *J* = 2.6 Hz, 1H), 7.13 (br s, 1H), 4.42 (q, *J* = 6.5 Hz, 1H), 3.91 (s, 3H), 3.24 (s, 3H), 1.50 (d, *J* = 6.5 Hz, 3H). ^{13}C{^{1}H} NMR (101 MHz, $CDCl_3$) δ 157.76, 138.73, 134.29, 129.43, 128.86, 127.33, 125.21, 124.84, 118.99, 105.88, 79.85, 56.55, 55.44, 23.93. IR (ATR) 3061, 2975, 1604, 1482, 1263, 1109, 1088, 1026, 855, 816 cm^{-1}.

2-(4-(1-methoxyethyl)benzyl)cyclopentan-1-one [*Loxoprofen derivative*] (2c):[2]

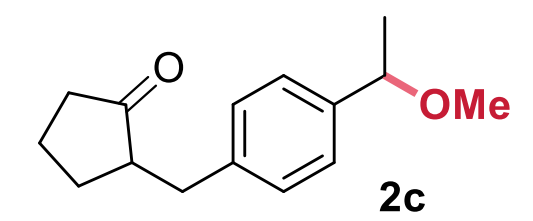

2c

Following the general procedure **A**, the title compound was isolated as a colourless oil with 95% yield (88.3 mg). ^{1}H NMR (400 MHz, $CDCl_3$) δ 7.23-7.20 (m, 2H), 7.16-7.13 (m, 2H), 4.47 (q, *J* = 6.4 Hz, 1H), 3.22 (s, 3H), 3.14 (dd, *J* = 13.8, 4.2 Hz, 1H), 2.53 (dd, *J* = 13.9, 9.5 Hz, 1H), 2.39-2.30 (m, 2H), 2.16-2.06 (m, 2H), 2.00-1.92 (m, 1H), 1.79-1.67 (m, 1H), 1.61-1.49 (m, 1H), 1.42 (d, *J* = 6.5 Hz, 3H). ^{13}C{^{1}H} NMR (101 MHz, $CDCl_3$) δ 220.37, 141.46, 139.32, 129.06, 126.43, 79.49, 56.50, 51.14, 38.32, 35.41, 29.35, 23.81, 20.67. IR (ATR) 3049, 2971, 1735, 1512, 1451, 1153, 1109, 1059, 858, 822 cm^{-1}.

2-fluoro-4-(1-methoxyethyl)-1,1'-biphenyl [*Flurbiprofen derivative*] (2d):[3]

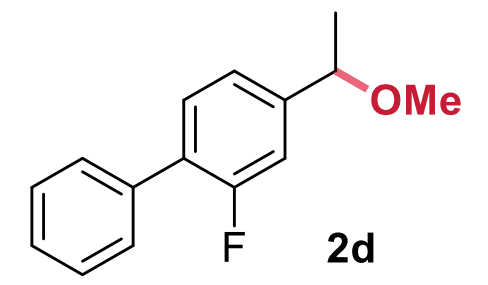

2d

Following the general procedure **A**, the title compound was isolated as a slightly yellowish, transparent oil with 95% yield (87.6 mg). ^{1}H NMR (400 MHz, $CDCl_3$) δ 7.56-7.53 (m, 2H), 7.46-7.34 (m, 4H), 7.16-7.11 (m, 2H), 4.32 (q, *J* = 6.5 Hz, 1H), 3.28 (s, 3H), 1.46 (d, *J* = 6.4 Hz, 3H). ^{13}C{^{1}H} NMR (101 MHz, $CDCl_3$) δ 160.01 (d, *J* = 248.3 Hz), 145.54 (d, *J* = 6.9 Hz), 135.82 (d, *J* = 1.2 Hz), 130.88 (d, *J* = 3.8 Hz), 129.11 (d, *J* = 3.0 Hz), 128.57, 128.15 (d, *J* = 13.8 Hz), 127.74, 122.21 (d, *J* = 3.3 Hz), 113.83 (d, *J* = 23.3 Hz), 79.03 (d, *J* = 1.4 Hz), 56.78, 23.85. IR (ATR) 3032, 2976, 1582, 1483, 1413, 1268, 1110, 1086, 766, 696 cm^{-1}.

1-(1-methoxyethyl)-3-phenoxybenzene [*Fenoprofen derivative*] (2e):

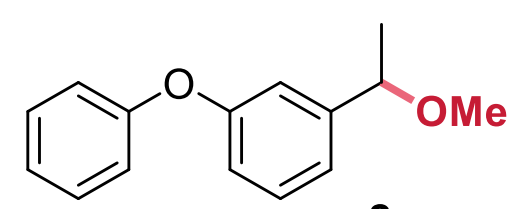

2e

Following the general procedure **A**, the title compound was isolated as a slightly yellowish, transparent oil with 98% yield (89.4 mg). ^{1}H NMR (400 MHz, $CDCl_3$) δ 7.36-7.25 (m, 3H), 7.14-6.96 (m, 5H), 6.90 (ddd, *J* = 8.1, 2.5, 1.0 Hz, 1H), 4.26 (q, *J* = 6.5 Hz, 1H), 3.23 (s, 3H), 1.41 (d, *J* = 6.5 Hz, 3H). ^{13}C{^{1}H} NMR (101 MHz, $CDCl_3$) δ 157.59, 157.31, 145.96, 129.87, 123.36, 121.13, 118.99, 117.84, 116.71, 79.43,

56.62, 23.91. IR (ATR) 3039, 2975, 1582, 1483, 1443, 1239, 1213, 1113, 1095, 754, 691 cm^{-1}. HRMS (ESI) m/z: calcd for $C_{15}H_{16}O_2Na$ [M + Na]$^+$ 251.1048, found 251.1054.

1-chloro-4-(1-methoxyethyl)benzene (2f):[4]

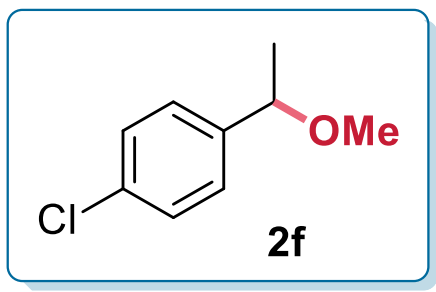

Following the general procedure **A**, the title compound was isolated as a colourless oil with 91% yield (62.0 mg). ^{1}H NMR (400 MHz, $CDCl_3$) δ 7.33-7.30 (m, 2H), 7.27-7.22 (m, 2H), 4.27 (q, J = 6.5 Hz, 1H), 3.21 (s, 3H), 1.41 (d, J = 6.5 Hz, 3H). ^{13}C{^{1}H} NMR (101 MHz, $CDCl_3$) 142.22, 133.22, 128.75, 127.69, 79.11, 56.59, 23.93. IR (ATR) 3045, 2978, 1735, 1489, 1089, 1060, 1014, 826 cm^{-1}.

(1-methoxypropyl)benzene (2g):[5]

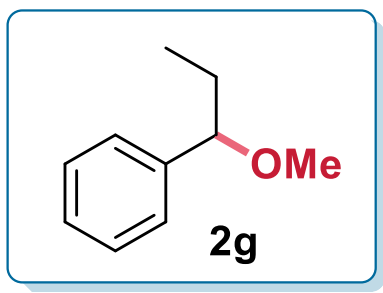

Following the general procedure **A**, the title compound was isolated as a colourless liquid with 91% yield (52.0 mg). ^{1}H NMR (400 MHz, $CDCl_3$) δ 7.36-7.32 (m, 2H), 7.30-7.23 (m, 3H), 4.01 (t, J = 6.6 Hz, 1H), 3.21 (s, 3H), 1.87-1.78 (m, 1H), 1.87-1.78 (m, 1H), 0.87 (t, J = 7.4 Hz, 3H).^{13}C{^{1}H} NMR (101 MHz, $CDCl_3$) 142.33, 128.41, 127.55, 126.91, 85.67, 56.75, 31.02, 10.30. IR (ATR) 3029, 2932, 1493, 1452, 1267, 1095, 1082, 761, 699 cm^{-1}.

(1-methoxy-2-methylpropyl)benzene (2h):[6]

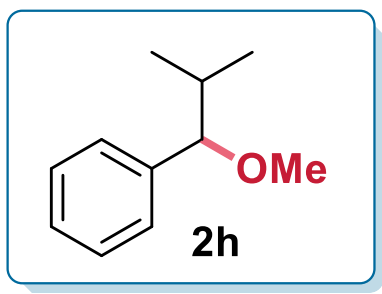

Following the general procedure **A**, the title compound was isolated as a colourless oil with 85% yield (56.0 mg). ^{1}H NMR (400 MHz, $CDCl_3$) δ 7.35-7.30 (m, 2H), 7.29-7.21 (m, 3H), 3.75 (d, J = 7.2 Hz, 1H), 3.19 (s, 3H), 1.91 (oct, J = 6.8 Hz, 1H), 0.99 (d, J = 6.7 Hz, 3H), 0.73 (d, J = 6.8 Hz, 3H). ^{13}C{^{1}H} NMR (101 MHz, $CDCl_3$) 141.21, 128.16, 127.60, 127.45, 89.92, 57.09, 34.86, 19.08. IR (ATR) 3028, 2956, 1470, 1453, 1093, 757, 700 cm^{-1}.

(cyclohexyl(methoxy)methyl)benzene (2i):[7]

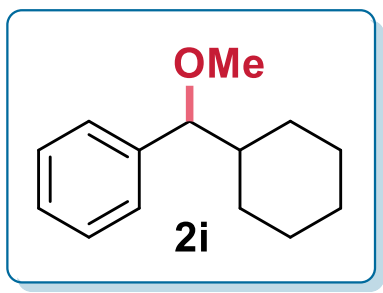

Following the general procedure **A**, the title compound was isolated as a colourless oil with 95% yield (77.7 mg). ^{1}H NMR (400 MHz, $CDCl_3$) δ 7.38-7.29 (m, 2H), 7.28-7.19 (m, 3H), 3.78 (d, J = 7.3 Hz, 1H), 3.18 (s, 3H), 2.08-1.96 (m, 1H), 1.78-1.50 (m, 4H), 1.40-0.81 (m, 6H). ^{13}C{^{1}H} NMR (101 MHz, $CDCl_3$) 141.20, 128.15, 127.61, 127.43, 89.18, 57.04, 44.54, 29.65, 29.42, 26.68, 26.28, 26.22. IR (ATR) 3027, 2921, 2850, 1491, 1450, 1104, 1086, 757, 700 cm^{-1}.

methoxytriphenylmethane (2j):[8]

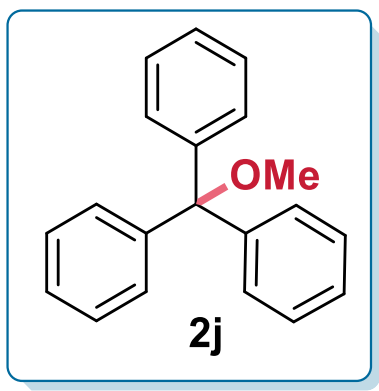

Following the general procedure **A**, the title compound was isolated as a white solid with 97% yield (106.8 mg). mp: 84.5 – 86.0 °C. ^{1}H NMR (400 MHz, $CDCl_3$) δ 7.50-7.40 (m, 6H), 7.33-7.20 (m, 9H), 3.06 (s, 3H). ^{13}C{^{1}H} NMR (101 MHz, $CDCl_3$) 144.12, 128.88, 127.90, 127.05, 87.21, 52.23. IR (ATR) 3054, 2827, 1489, 1447, 1071, 753, 698, 647 cm^{-1}.

2-(4-(1-methoxypropyl)phenyl)isoindolin-1-one [*Indobufen derivative*] (2k):

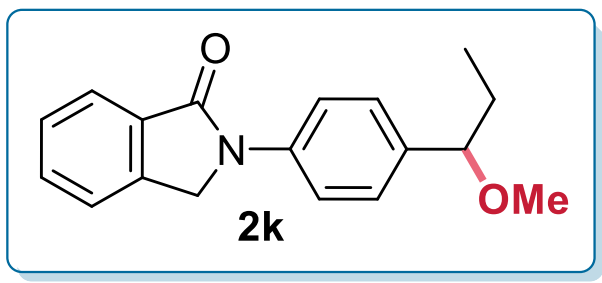

Following the general procedure **B**, the title compound was isolated as a beige solid with 98% yield (110.3 mg). mp: 99.0 – 101.0 °C. ^{1}H NMR (400 MHz, $CDCl_3$) δ 7.96-7.90 (m, 1H), 7.88-7.82 (m, 2H), 7.62-7.57 (m, 1H), 7.54-7.48 (m, 2H), 7.37-7.32 (m, 2H), 4.87 (s, 2H), 4.03 (t, *J* = 6.6 Hz, 1H), 3.23 (s, 3H), 1.92-1.78 (m, 1H), 1.74-1.62 (m, 1H), 0.89 (t, *J* = 7.4 Hz, 3H). ^{13}C{^{1}H} NMR (101 MHz, $CDCl_3$) 167.60, 140.24, 138.88, 138.50, 133.38, 132.19, 128.53, 127.72, 124.30, 122.75, 119.52, 85.18, 56.77, 50.91, 30.96, 10.30. IR (ATR) 3045, 2928, 1682, 1609, 1511, 1466, 1443, 1379, 1084, 819, 728 cm^{-1}. HRMS (ESI) m/z: calcd for $C_{18}H_{20}NO_2$ $[M + H]^+$ 282.1494, found 282.1494.

(1-methoxy-2,3-dihydro-1*H*-pyrrolizin-5-yl)(phenyl)methanone [*Ketorolac derivative*] (2l):

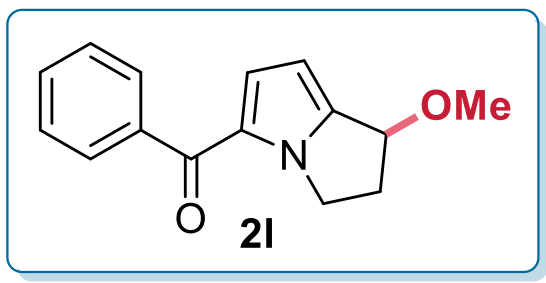

Following the general procedure **B**, the title compound was isolated as a white solid with 98 % yield (94.6 mg). mp: 91.0 – 92.0 °C. ^{1}H NMR (400 MHz, $CDCl_3$) δ 7.84-7.81 (m, 2H), 7.55-7.50 (m, 1H), 7.48-7.43 (m, 2H), 6.84 (br d, *J* = 3.9 Hz, 1H), 6.19 (d, *J* = 4.0 Hz, 1H), 4.75 (dd, *J* = 6.8, 2.2 Hz, 1H), 4.52-4.44 (m, 2H), 3.40 (s, 3H), 2.84-2.74 (m, 1H), 2.60-2.53 (m, 1H). ^{13}C{^{1}H} NMR (101 MHz, $CDCl_3$) 185.49, 144.12, 139.43, 131.55, 129.05, 128.28, 127.25, 124.60, 103.75, 75.82, 56.26, 47.00, 35.25. IR (ATR) 3006, 2923, 2823, 1614, 1574, 1462, 1435, 1272, 1087, 888, 723 cm^{-1}. HRMS (ESI) m/z: calcd for $C_{15}H_{16}NO_2$ $[M + H]^+$ 242.1181, found 242.1191.

7-(1-methoxyethyl)-5*H*-chromeno[2,3-*b*]pyridine [*Pranoprofen derivative*] (2m):

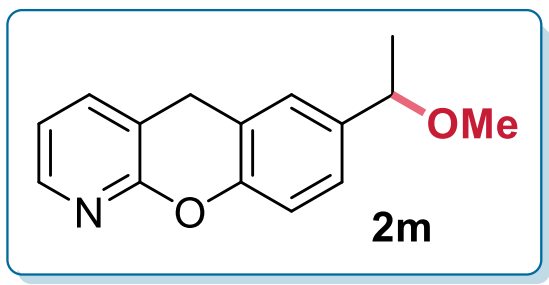

Following the general procedure **B**, with Et_3N (12.6 µL, 0.09 mmol, 0.225 eq.), the title compound was isolated as a white solid with 98% yield (94.4 mg). mp: 111 – 114 °C. ^{1}H NMR (400 MHz, $CDCl_3$) δ 8.18-8.16 (m, 1H), 7.55-7.52 (m, 1H), 7.15-7.12 (m,3H), 7.03 (dd, *J* = 7.3, 4.8 Hz, 1H), 4.26 (q, *J* = 6.4 Hz, 1H), 4.11 (br s, 2H), 3.23 (s, 3H), 1.43 (d, *J* = 6.5 Hz, 3H). ^{13}C{^{1}H} NMR (101 MHz, $CDCl_3$) 158.62, 151.12, 146.79, 139.09, 138.46, 126.28, 126.16, 119.89, 119.59, 117.25, 115.53, 79.15, 56.50, 28.31, 23.89. IR (ATR) 3050, 2979, 2820, 1606, 1578, 1504, 1439, 1422, 1269, 1097, 1088, 816, 773 cm^{-1}. HRMS (ESI) m/z: calcd for $C_{15}H_{16}NO_2$ $[M + H]^+$ 242.1181, found 242.1188.

(5-(1-methoxyethyl)thiophen-2-yl)(phenyl)methanone [*Tiaprofenic acid derivative*] (2n):[9]

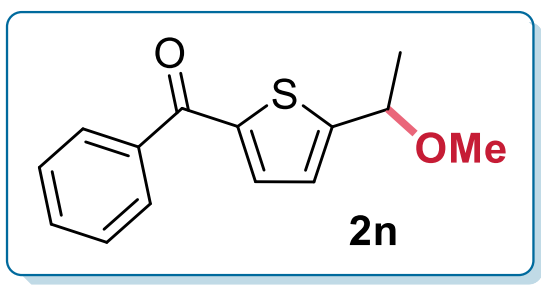

Following the general procedure **B**, the title compound was isolated as a colourless oil with 99% yield (97.4 mg). ^{1}H NMR (400 MHz, $CDCl_3$) δ 7.87-7.82 (m, 2H), 7.61-7.55 (m, 1H), 7.53 (d, *J* = 3.8 Hz, 1H), 7.51-7.46 (m, 2H), 7.01 (dd, *J* = 3.8, 0.7 Hz, 1H), 4.60 (q, *J* = 6.5 Hz, 1H), 3.35 (s, 3H), 1.57 (d, *J* = 6.5 Hz 3H). ^{13}C{^{1}H} NMR (101 MHz, $CDCl_3$) 188.30, 157.49, 142.41, 138.26, 134.90, 132.31, 129.26, 128.53, 125.04, 75.38, 56.90, 23.84. IR (ATR) 3062, 2977, 2822, 1629, 1598, 1447, 1286, 1109, 1085, 864, 699 cm^{-1}.

(4-(1-methoxyethyl)phenyl)(phenyl)methanone [*Ketoprofen derivative*] (2o):[10]

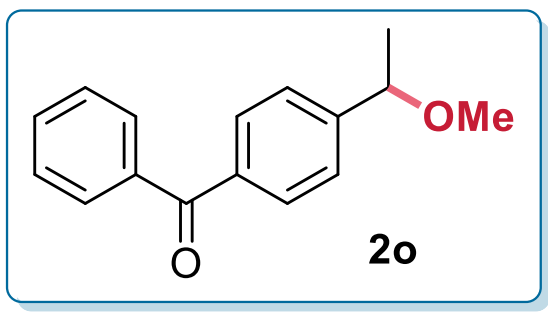

Following the general procedure **B**, the title compound was isolated as a colourless oil using silica gel column chromatography eluting with hexane and ethyl acetate (90:10), R_f = 0.22, with an isolated yield 72% (69.0 mg). ^{1}H NMR (400 MHz, $CDCl_3$) δ 7.82-7.79 (m, 2H), 7.77-7.74 (m, 1H), 7.72-7.68 (m, 1H), 7.62-7.54 (m, 2H), 7.51-7.45 (m, 3H), 4.37 (q, *J* = 6.5 Hz, 1H), 3.26 (s, 3H), 1.46 (d, *J* = 6.5 Hz 3H). ^{13}C{^{1}H} NMR (101 MHz, $CDCl_3$) 196.83, 144.16, 137.91, 137.75, 132.56, 130.24, 130.15, 129.47, 128.58, 128.42, 127.91, 79.38, 56.71, 23.93. IR (ATR) 3059, 2975, 1656, 1597, 1446, 1283, 1213, 1113, 1096, 720, 700 cm^{-1}.

2-(1-methoxyethyl)dibenzo[*b,f*]thiepin-10(11*H*)-one [*Zaltoprofen derivative*] (2p):

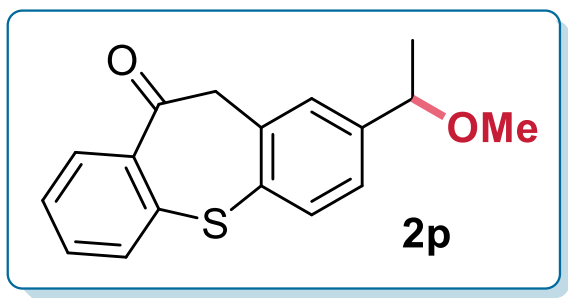

Following the general procedure **B**, the title compound was isolated as a colourless oil using silica gel column chromatography eluting with hexane and ethyl acetate (90:10), R_f = 0.18, with an isolated yield 48% (54.6 mg). ^{1}H NMR (400 MHz, $CDCl_3$) δ 8.21 (ddd, *J* = 7.9, 1.7, 0.5 Hz, 1H), 7.65-7.58 (m, 2H), 7.47-7.37 (m, 2H), 7.32 (ddd, *J* = 7.9, 7.2, 1.3 Hz, 1H), 7.16 (ddd, *J* = 7.9, 1.9, 0.5 Hz, 1H), 4.38 (s, 2H), 4.29 (q, *J* = 6.5 Hz, 1H), 3.21 (s, 3H), 1.40 (d, *J* = 6.5 Hz, 3H). ^{13}C{^{1}H} NMR (101 MHz, $CDCl_3$) 191.59, 146.20, 140.46, 137.93, 136.32, 133.48, 132.63, 131.66, 131.55, 130.98, 127.38, 126.95, 125.03, 79.18, 56.78, 51.24, 23.94. IR (ATR) 3055, 2973, 1671, 1586, 1458, 1428, 1278, 1110, 1072, 754 cm^{-1}. HRMS (ESI) m/z: calcd for $C_{17}H_{16}O_2SNa$ [M + Na]$^+$ 307.0769, found 307.0771.

(4-chlorophenyl)[5-methoxy-3-(methoxymethyl)-2-methyl-1*H*-indol-1-yl]methanone [*Indomethacin derivative*] (2r):

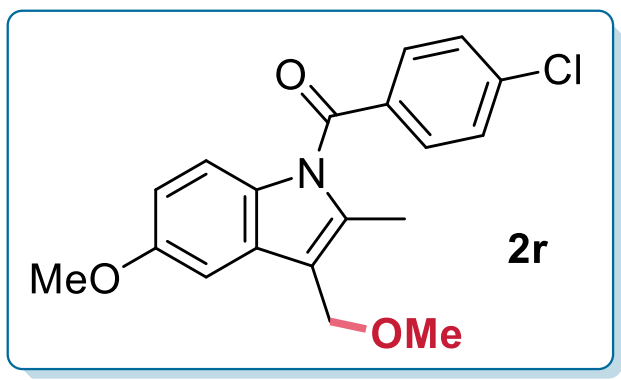

Following the general procedure **C**, the title compound was isolated as a yellow oil using silica gel column chromatography eluting with dichloromethane, R_f = 0.3, with an isolated yield 67% (92.5 mg). ^{1}H NMR (400 MHz, $CDCl_3$) δ 7.70-7.63 (m, 2H), 7.49-7.44 (m, 2H), 7.06 (d, *J* = 2.5 Hz, 1H), 6.82 (dd, *J* = 9.0, 0.5 Hz, 1H), 6.66 (dd, *J* = 9.0, 2.6 Hz, 1H), 4.59 (s, 2H), 3.84 (s, 3H), 3.42 (s, 3H), 2.43 (s, 3H). ^{13}C{^{1}H} NMR (101 MHz, $CDCl_3$) 168.56, 156.19, 139.48, 137.04, 133.96, 131.34, 131.07, 130.74, 129.26, 116.14, 114.97, 111.84, 101.73, 64.97, 58.02, 55.86, 13.29. IR (ATR) 3068 2927, 1679, 1589, 1476, 1455, 1310, 1217, 1087, 753 cm^{-1}. HRMS (ESI) m/z: calcd for $C_{19}H_{18}ClNO_3Na$ [M + Na]$^+$ 366.0873, found 366.0877.

[5-(methoxymethyl)-1-methyl-1*H*-pyrrol-2-yl](*p*-tolyl)methanone [*Tolmetin derivative*] (2s):

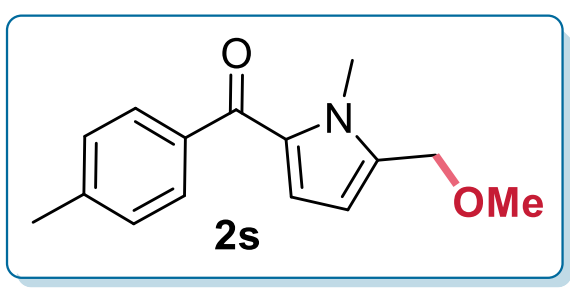

Following the general procedure **C**, the title compound was isolated as a white solid using silica gel column chromatography eluting hexane and ethyl acetate (90:10, v/v), R_f = 0.13, with an isolated yield 71% (70.0 mg). mp: 93.0 – 94.0 °C. ^{1}H NMR (400 MHz, $CDCl_3$) δ 7.75-7.68 (m, 2H), 7.28-7.20 (m, 2H), 6.64 (d, *J* = 4.0 Hz, 1H), 6.16 (d, *J* = 4.0 Hz, 1H), 4.46 (s, 2H), 3.99 (s, 3H), 3.37 (s, 3H), 2.42 (s, 3H). ^{13}C{^{1}H} NMR (101 MHz, $CDCl_3$) 186.36, 142.13, 137.59, 137.40, 132.20, 129.60, 128.83, 121.47, 110.18, 66.07, 57.93, 33.34, 21.66. IR (ATR) 3032, 2921, 2826, 1624, 1606, 1485, 1461, 1340, 1168, 1091, 742 cm^{-1}. HRMS (ESI) m/z: calcd for $C_{15}H_{18}NO_2$ [M + H]$^+$ 244.1338, found 244.1340.

1-(1-ethoxyethyl)-4-isobutylbenzene [*Ibuprofen derivative*] (3a):[11]

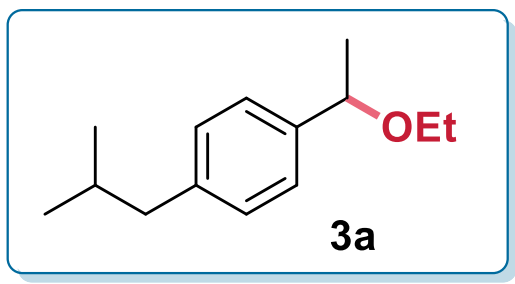

Following the general procedure **A**, the title compound was isolated as a slightly yellowish, transparent liquid with 95% yield (78.5 mg). ^{1}H NMR (400 MHz, $CDCl_3$) δ 7.22-7.19 (m, 2H), 7.12-7.09 (m, 2H), 4.38 (q, *J* = 6.5 Hz, 1H), 3.35 (q, *J* = 7.2 Hz, 2H), 2.46 (d, *J* = 7.2 Hz, 2H), 1.94-1.78 (m, 1H), 1.43 (d, *J* = 6.5 Hz, 3H), 1.18 (t, *J* = 7.0 Hz, 3H), 0.90 (d, *J* = 6.6 Hz, 6H). ^{13}C{^{1}H} NMR (101 MHz, $CDCl_3$) δ 141.53, 140.87, 129.23, 126.02, 77.72, 63.92, 45.29, 30.37, 24.29, 22.55, 15.56. IR (ATR) 3047, 2972, 1511, 1465, 1367, 1100, 1065, 846, 799 cm^{-1}.

4-(1-ethoxyethyl)-2-fluoro-1,1'-biphenyl [*Flurbiprofen derivative*] (3b):

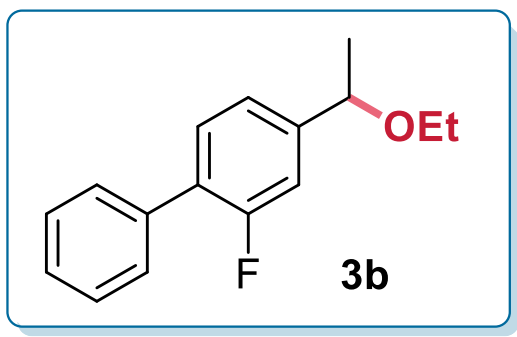

Following the general procedure **A**, the title compound was isolated as a slightly yellowish, transparent oil with 96% yield (93.8 mg). ^{1}H NMR (400 MHz, $CDCl_3$) δ 7.56-7.53 (m, 2H), 7.45-7.343(m, 4H), 7.16-7.11 (m, 2H), 4.43 (q, *J* = 6.5 Hz, 1H), 3.42 (qd, *J* = 7.0, 1.3 Hz, 2H), 1.46 (d, *J* = 6.5 Hz, 3H), 1.22 (t, *J* = 7.0 Hz, 3H). ^{13}C{^{1}H} NMR (101 MHz, $CDCl_3$) δ 160.02 (d, *J* = 248.0 Hz), 146.29 (d, *J* = 6.8 Hz), 135.88 (d, *J* = 1.2 Hz), 130.83 (d, *J* = 3.8 Hz), 129.11 (d, *J* = 2.8 Hz), 128.56, 128.01 (d, *J* = 13.5 Hz), 127.71, 122.21 (d, *J* = 3.4 Hz), 113.75 (d, *J* = 23.2 Hz), 77.14, 64.32, 24.21, 15.55. IR (ATR) 3033, 2974, 1582, 1483, 1415, 1267, 1099, 1010, 765, 696 cm^{-1}. HRMS (ESI) m/z: calcd for $C_{16}H_{17}OFNa$ [M + Na]$^{+}$ 267.1161, found 267.1169.

(4-(1-ethoxyethyl)phenyl)(phenyl)methanone [*Ketoprofen derivative*] (3c):

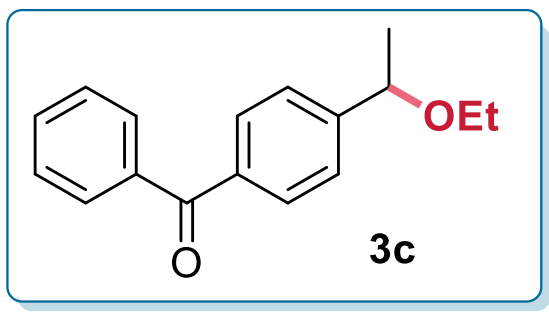

Following the general procedure **B**, the title compound was isolated as a colourless oil using silica gel column chromatography eluting with hexane and ethyl acetate (90:10), R_f = 0.22, with an isolated yield 48% (48.8 mg). ^{1}H NMR (400 MHz, $CDCl_3$) δ 7.82-7.75 (m, 2H), 7.76-7.75 (m, 1H), 7.71-7.68 (m, 1H), 7.61-7.56 (m, 2H), 7.51-7.44 (m, 3H), 4.48 (q, *J* = 6.5 Hz, 1H), 3.43-3.36 (m, 2H), 1.46 (d, *J* = 6.5 Hz 3H), 1.20 (t, *J* = 7.0 Hz 3H). ^{13}C{^{1}H} NMR (101 MHz, $CDCl_3$) 196.90, 144.85, 137.88, 137.79, 132.57, 130.22, 130.18, 129.33, 128.57, 128.42, 127.88, 77.52, 64.27, 24.33, 15.52 IR (ATR) 3060, 2973, 1658, 1597, 1446, 1284, 1207, 1106, 970, 722, 701 cm^{-1}. HRMS (ESI) m/z: calcd for $C_{17}H_{19}O_2$ [M + H]$^{+}$ 255.1385, found 255.1389.

4. ^{1}H and ^{13}C NMR spectra of products 2a-s and 3a-c

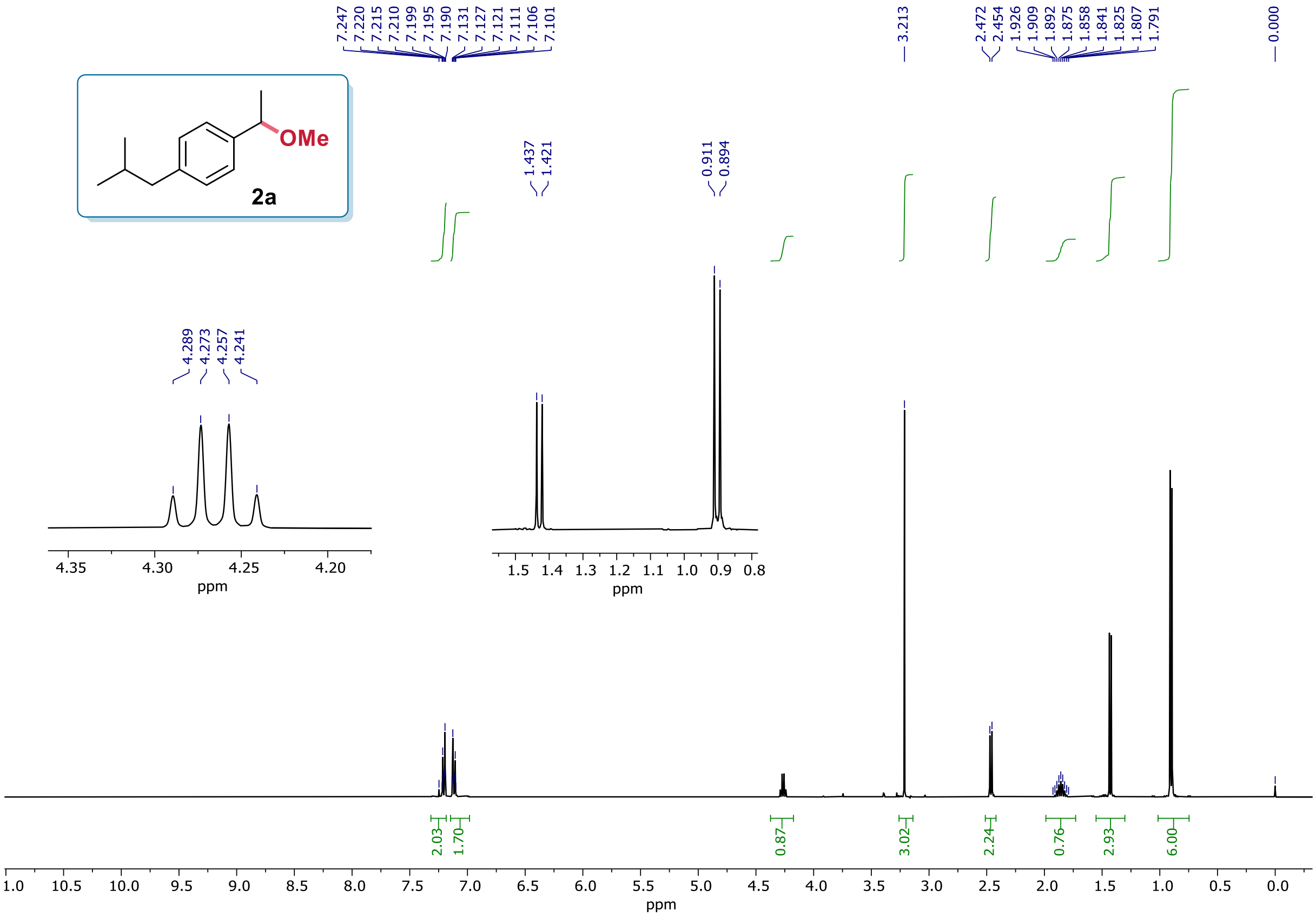

Figure. S1 ^{1}H NMR spectrum of **2a** in $CDCl_3$ (400 MHz)

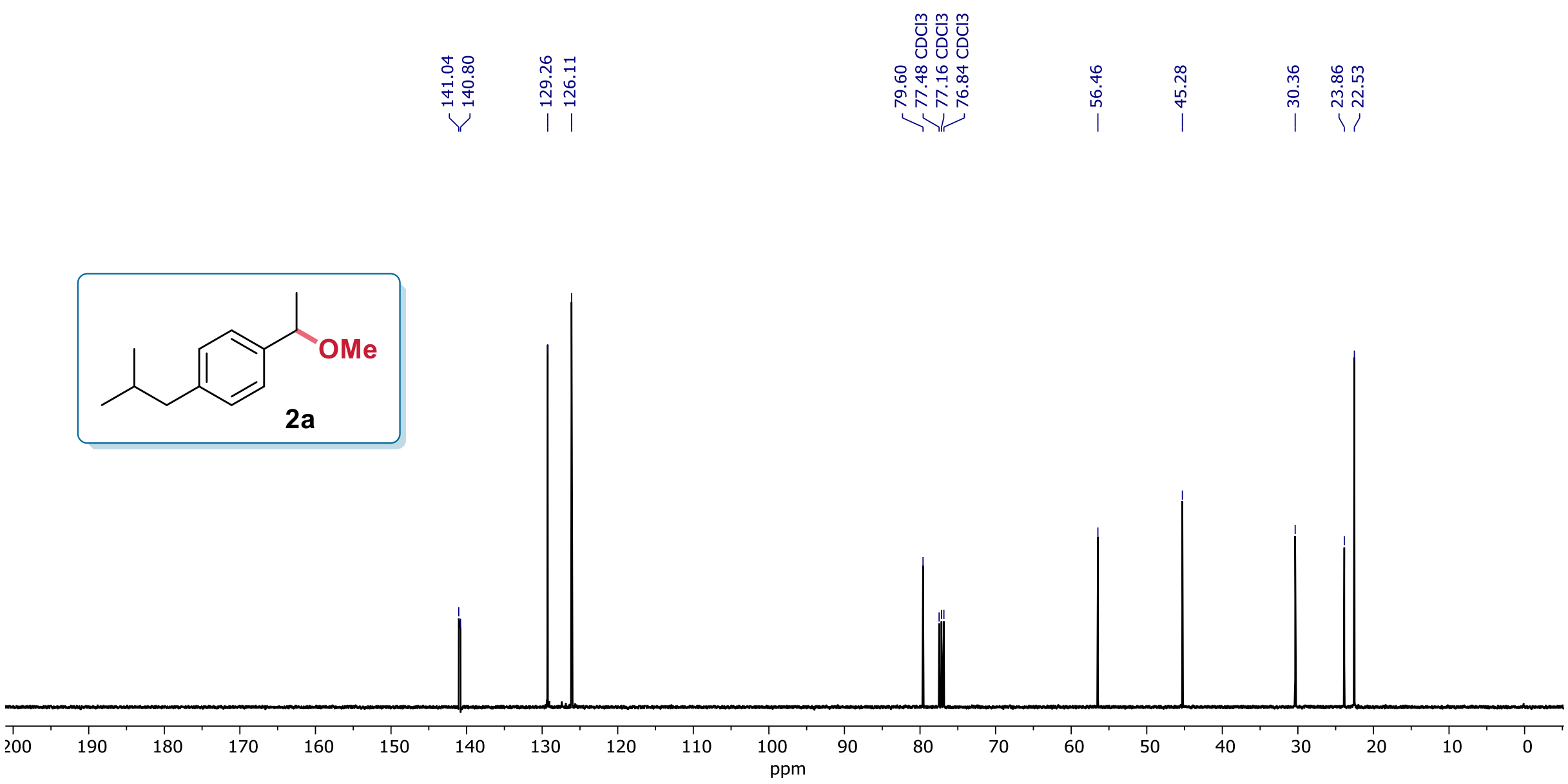

Figure. S2 $^{13}C\{^{1}H\}$ NMR spectrum of **2a** in $CDCl_3$ (101 MHz)

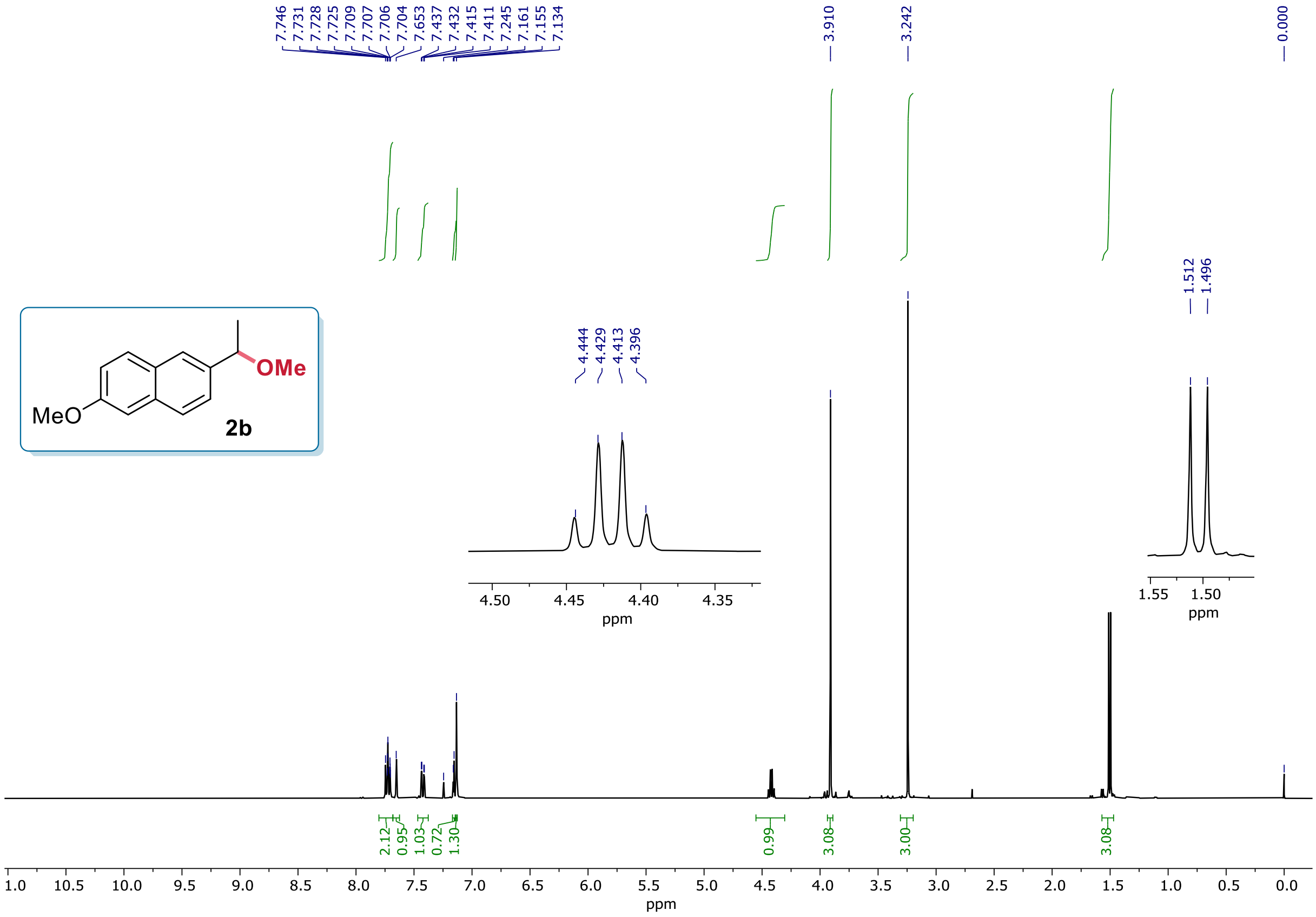

Figure. S3 ^{1}H NMR spectrum of **2b** in $CDCl_3$ (400 MHz)

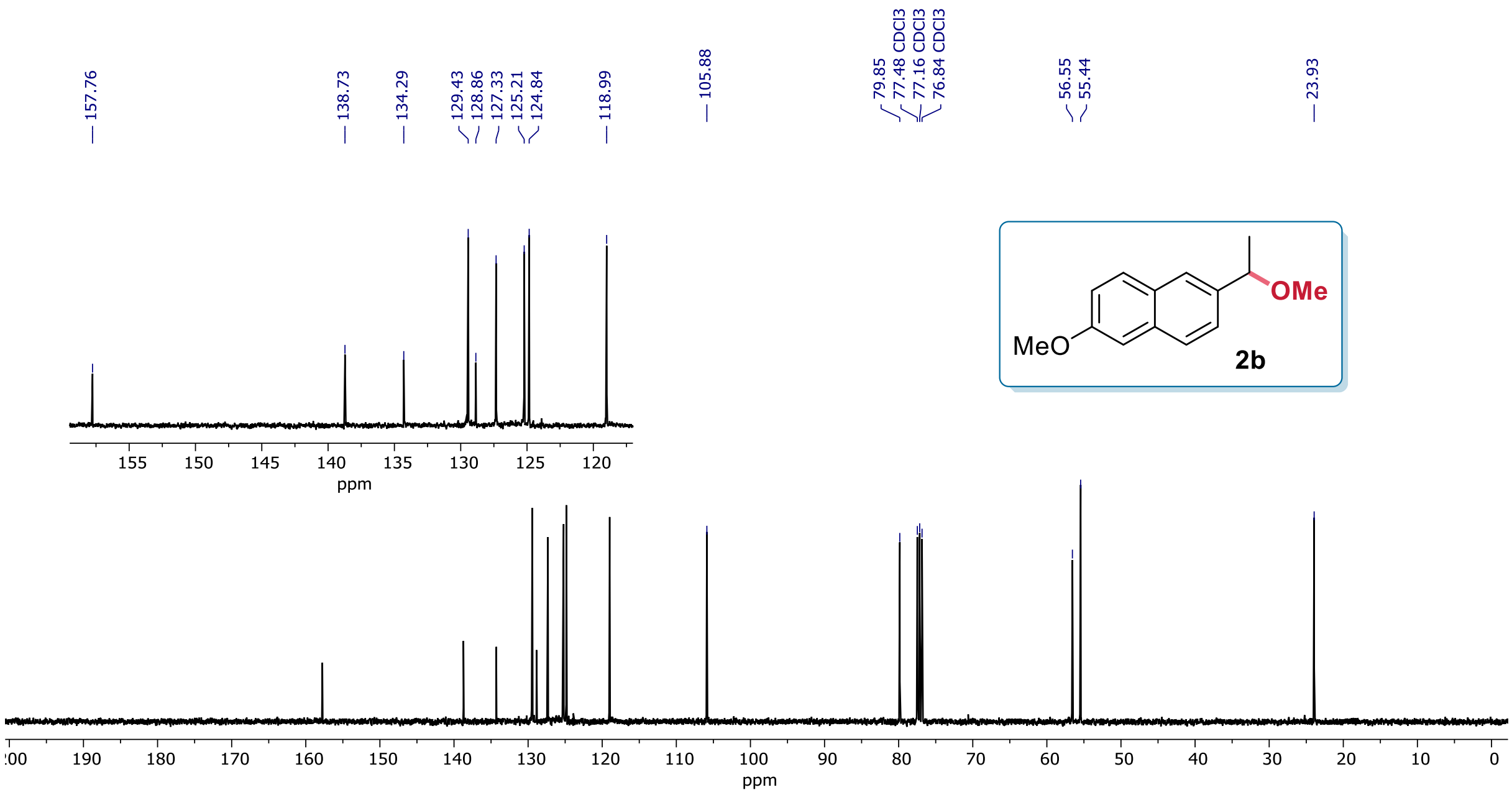

Figure. S4 $^{13}C\{^{1}H\}$ NMR spectrum of **2b** in $CDCl_3$ (101 MHz)

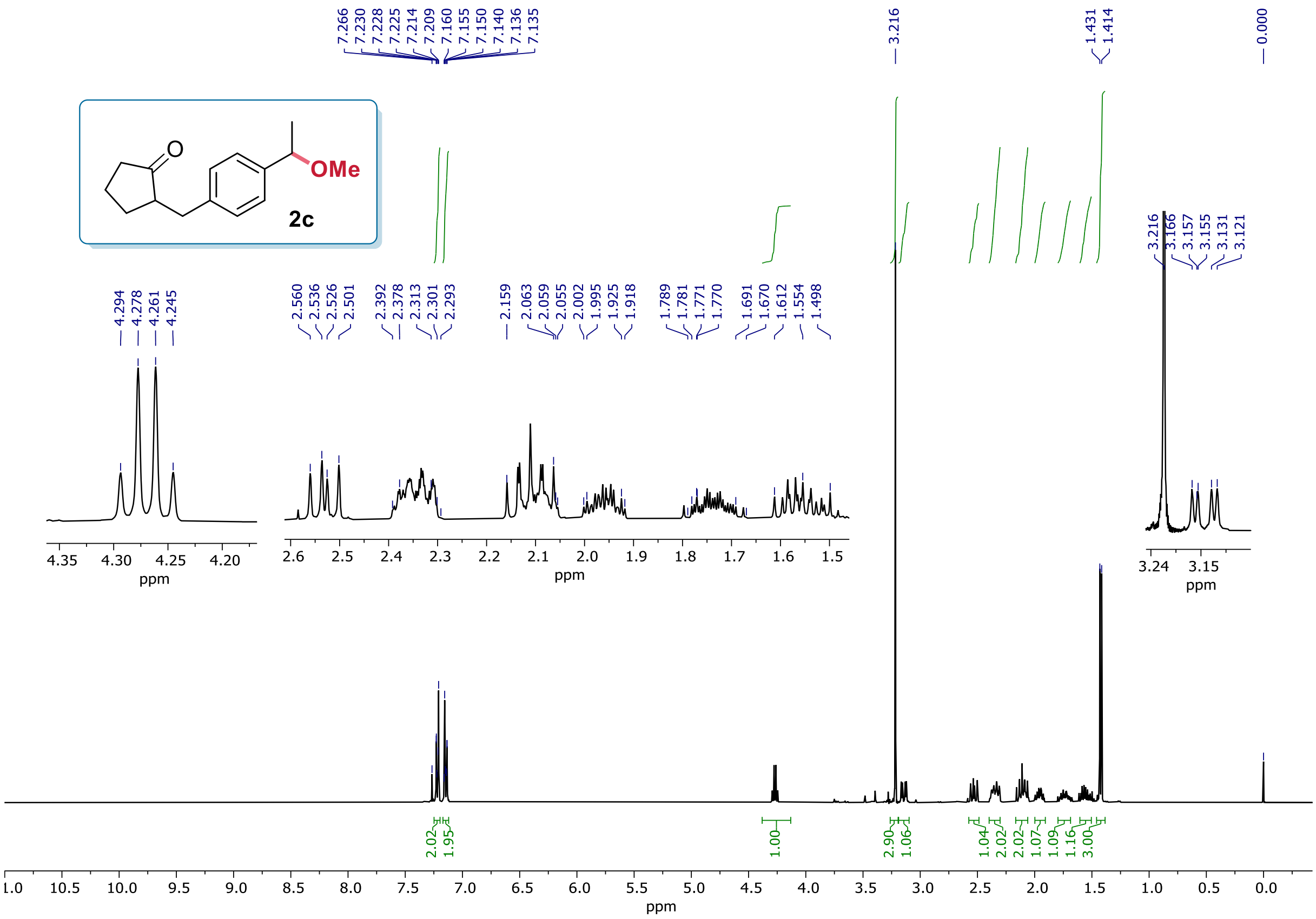

Figure. S5 ^{1}H NMR spectrum of **2c** in $CDCl_3$ (400 MHz)

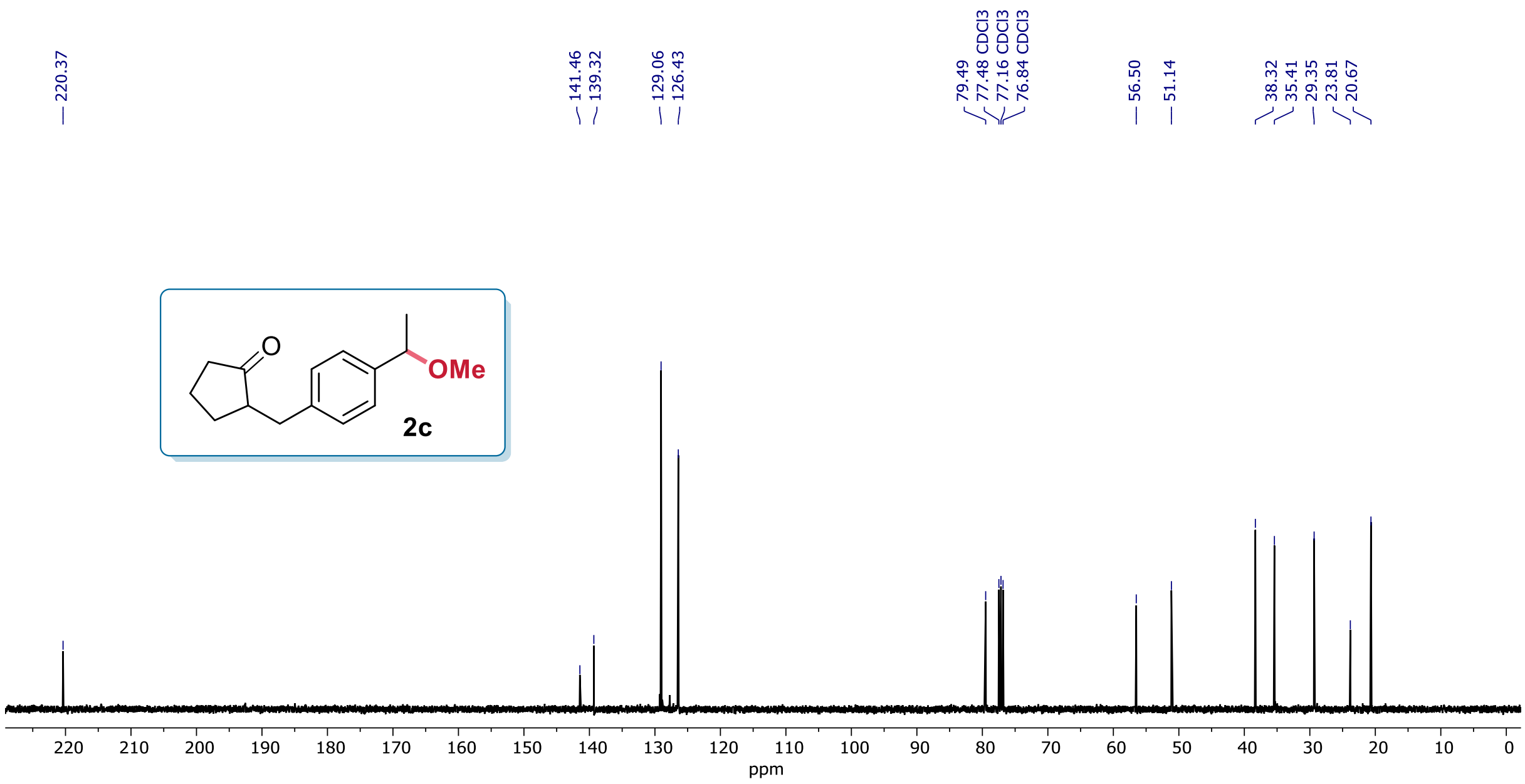

Figure. S6 $^{13}C\{^{1}H\}$ NMR spectrum of **2c** in $CDCl_3$ (101 MHz)

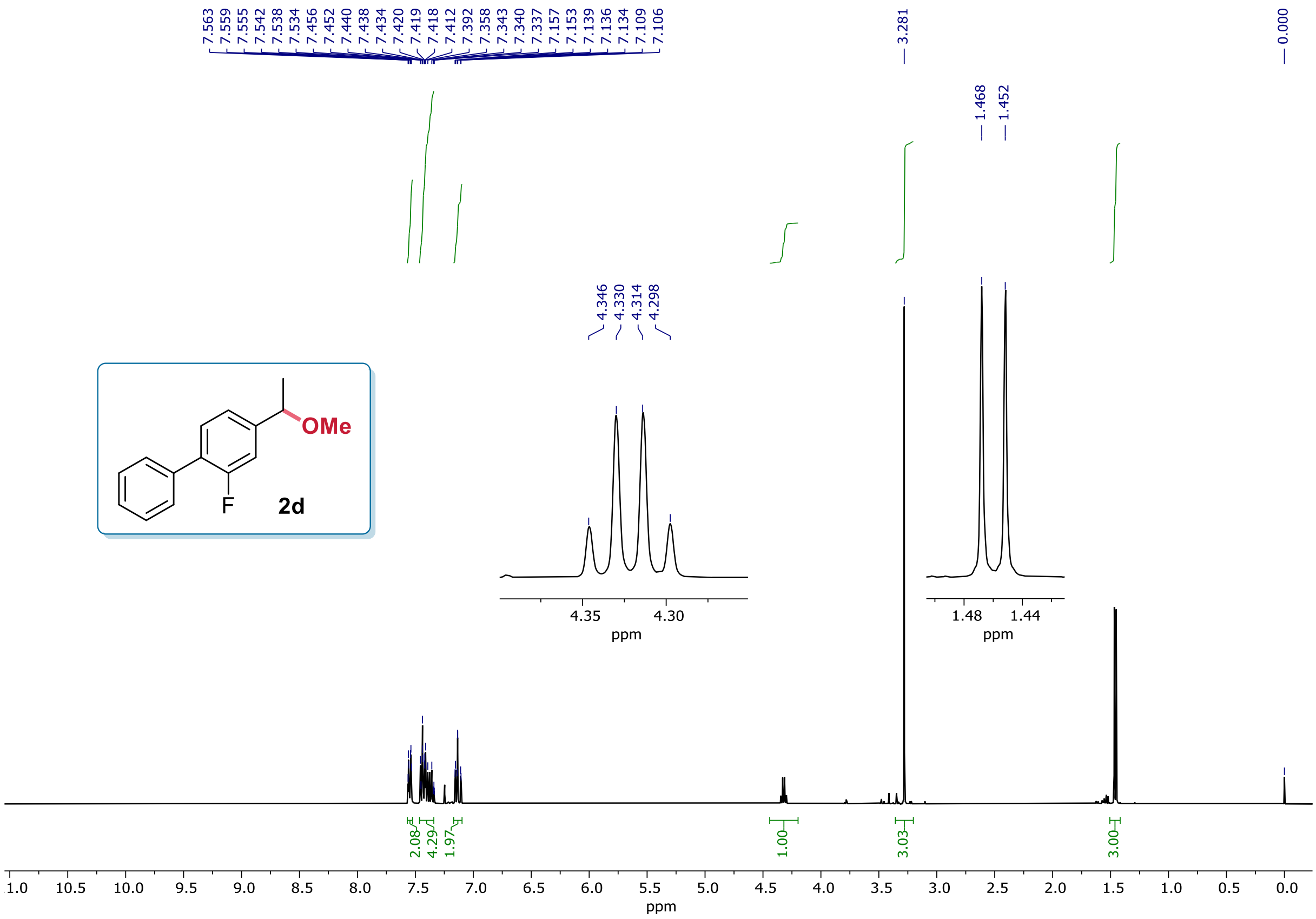

Figure. S7 ^{1}H NMR spectrum of **2d** in $CDCl_3$ (400 MHz)

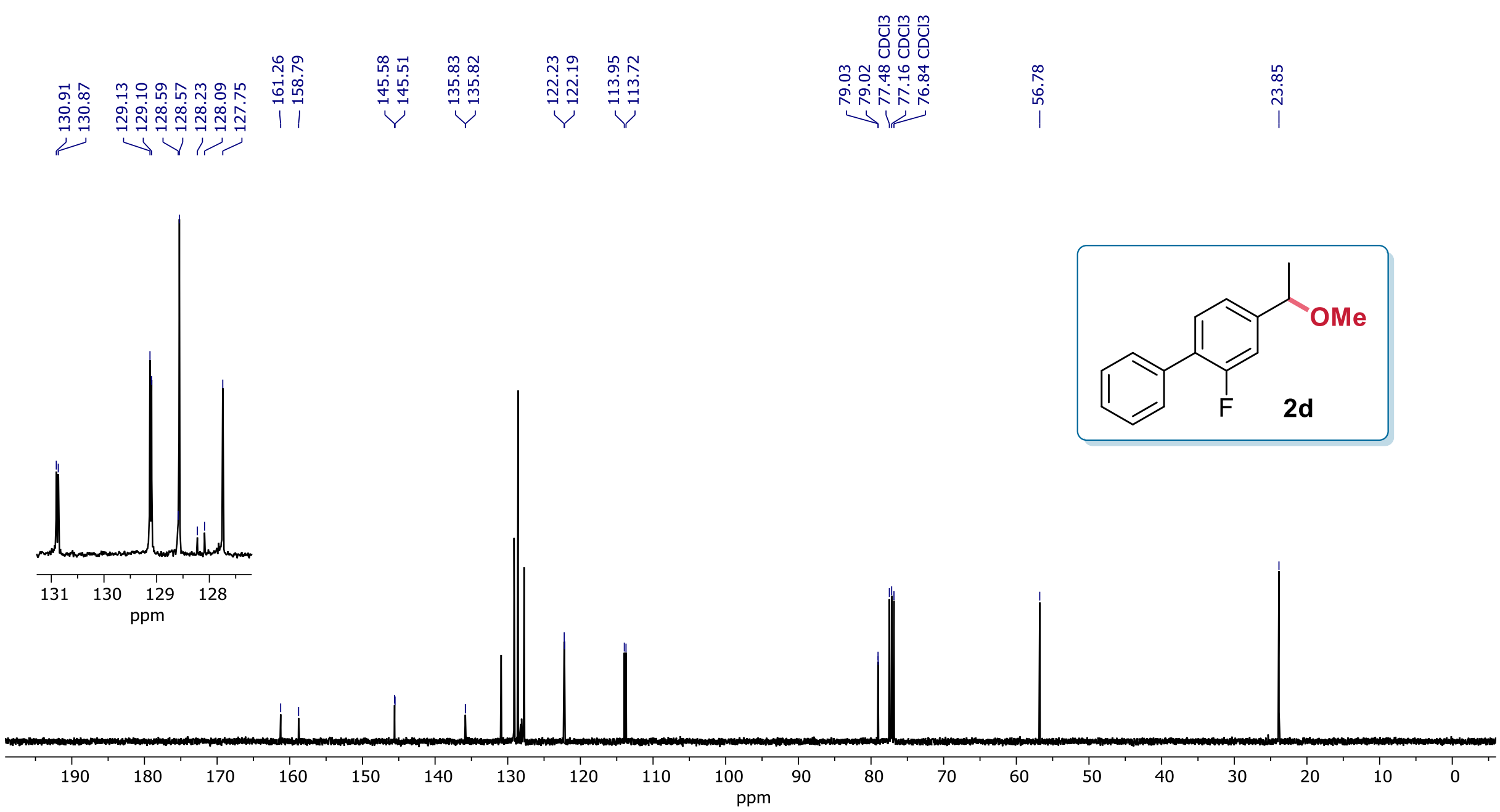

Figure. S8 $^{13}C\{^{1}H\}$ NMR spectrum of **2d** in $CDCl_3$ (101 MHz)

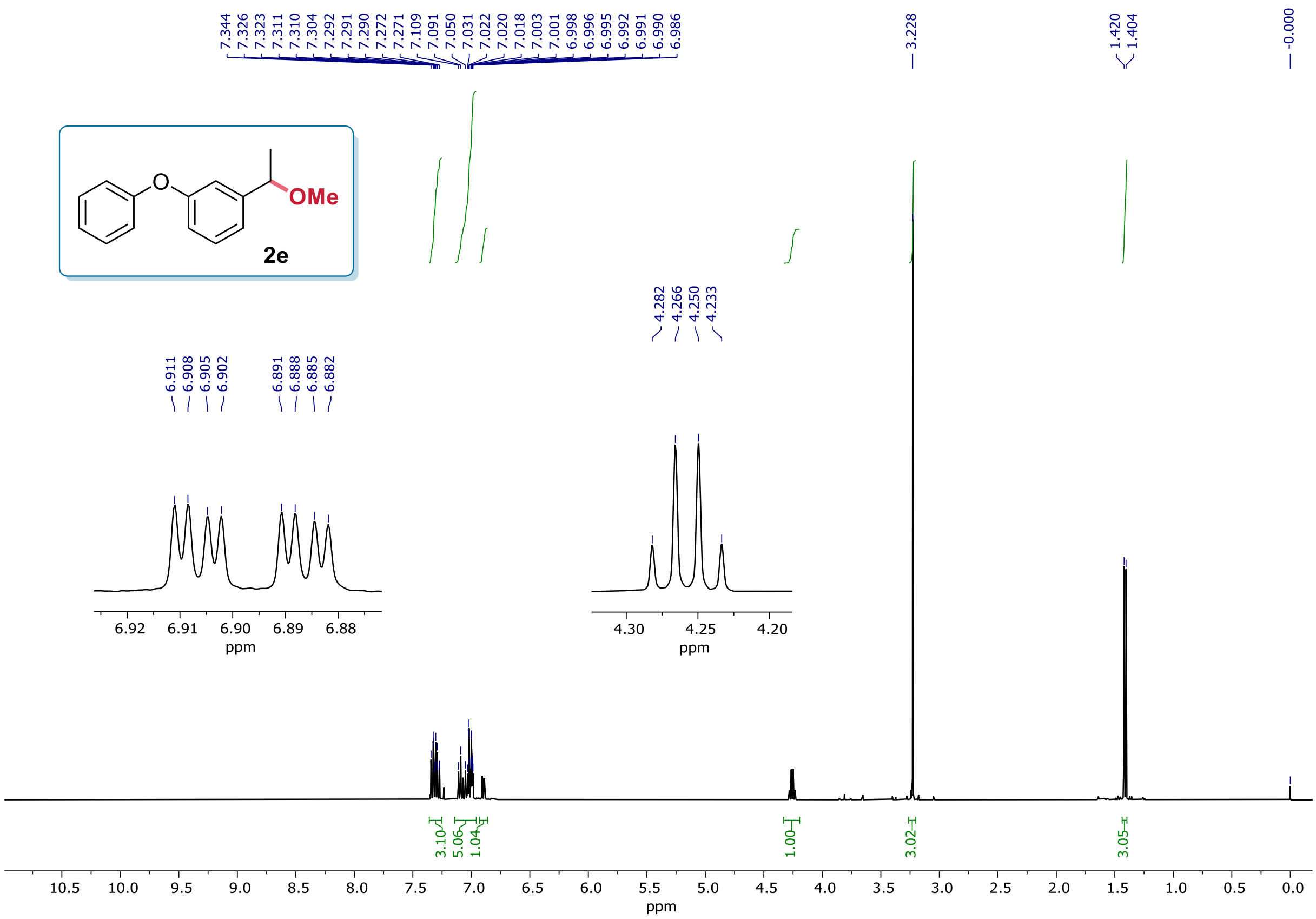

Figure. S9 ^{1}H NMR spectrum of **2e** in $CDCl_3$ (400 MHz)

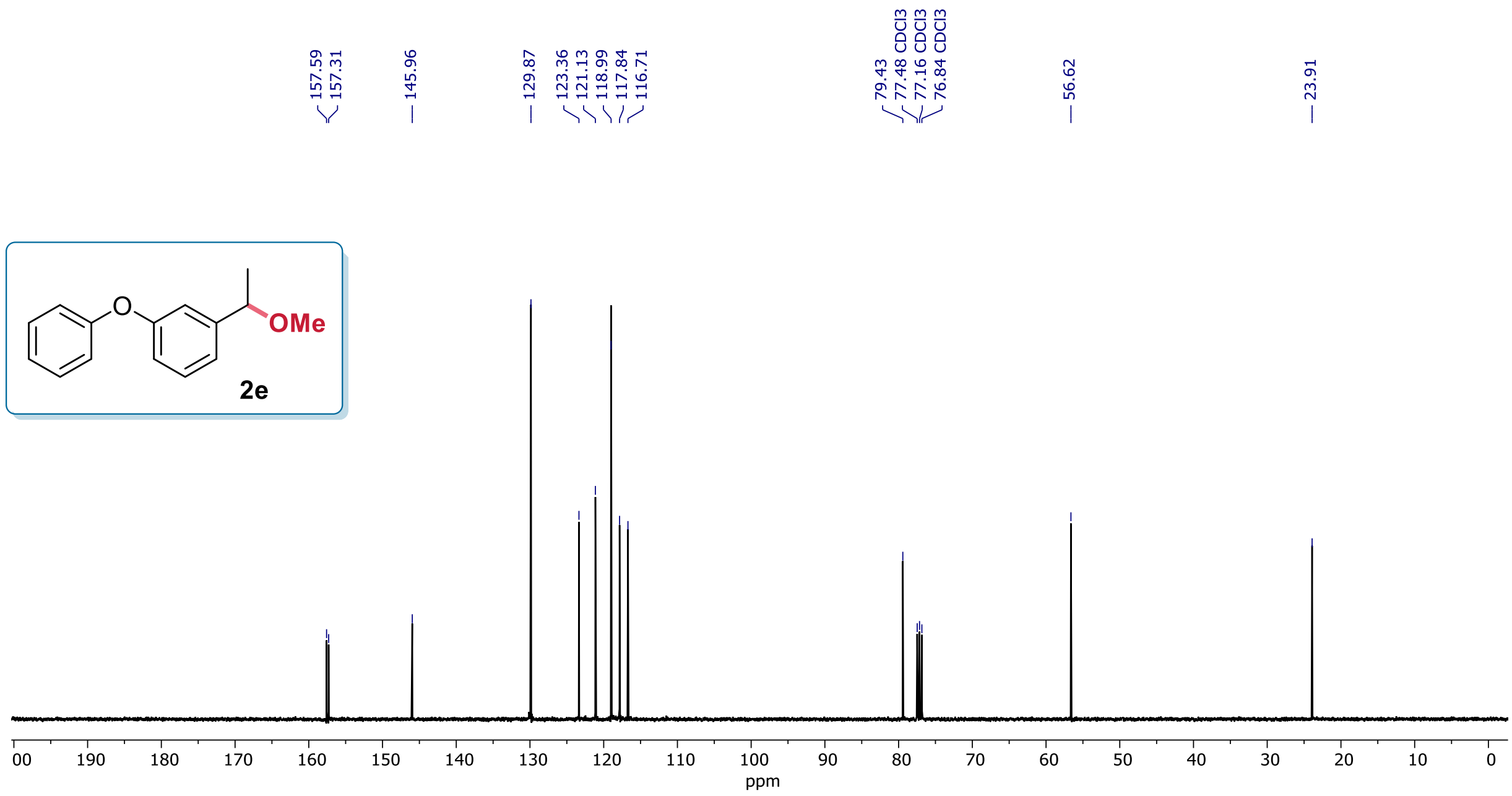

Figure. S10 $^{13}C\{^{1}H\}$ NMR spectrum of **2e** in $CDCl_3$ (101 MHz)

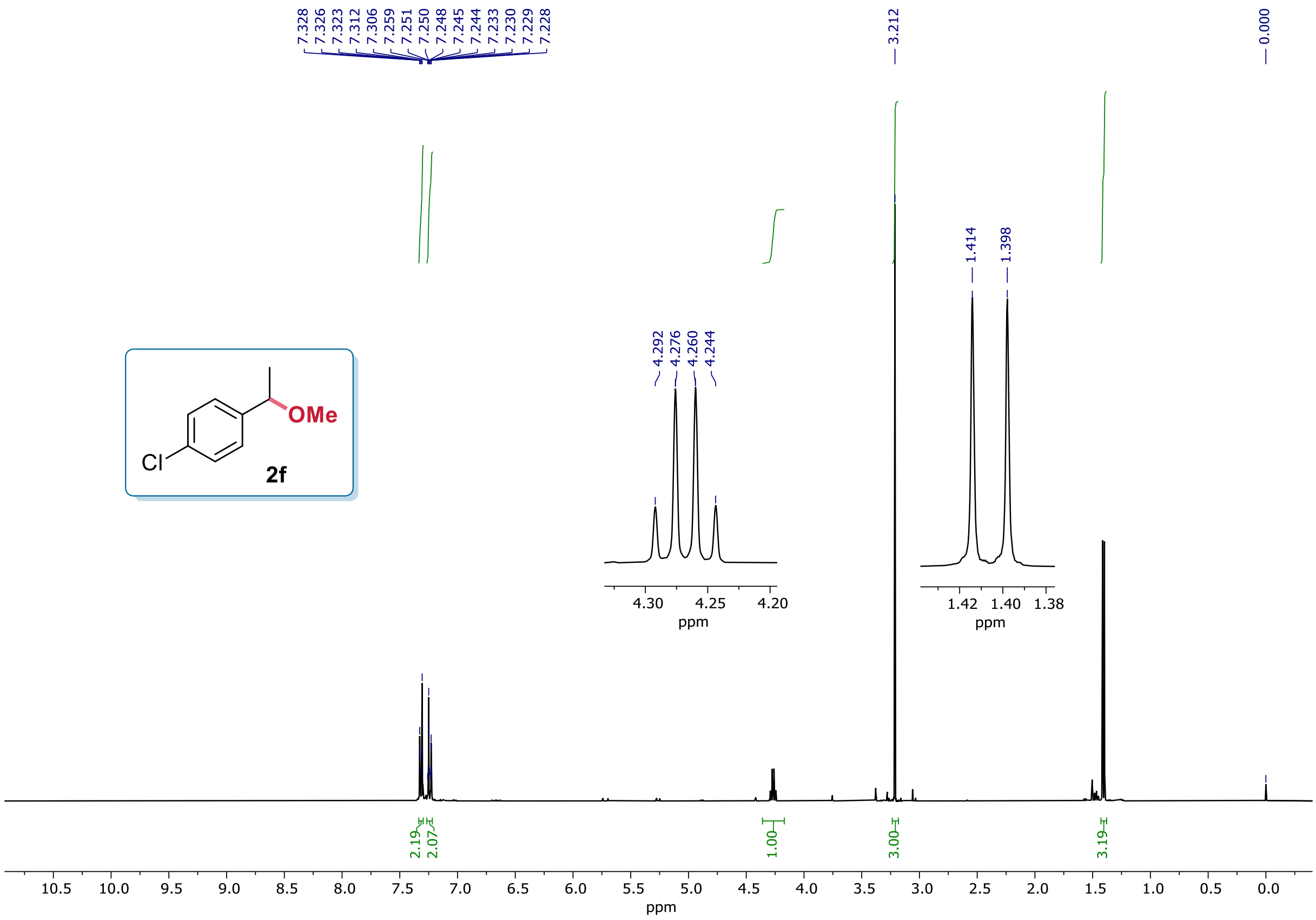

Figure. S11 ^{1}H NMR spectrum of **2f** in $CDCl_3$ (400 MHz)

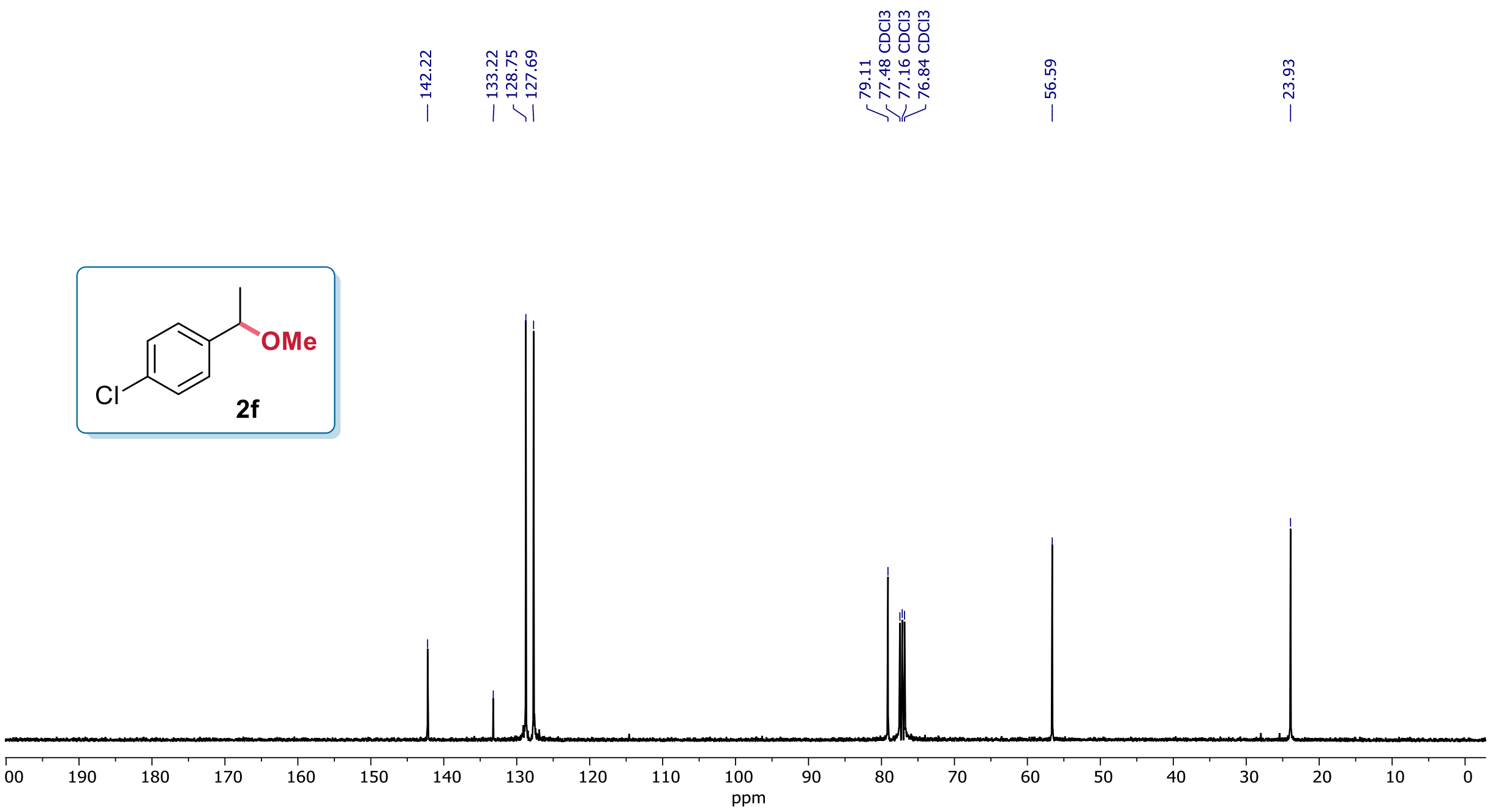

Figure. S12 $^{13}C\{^{1}H\}$ NMR spectrum of **2f** in $CDCl_3$ (101 MHz)

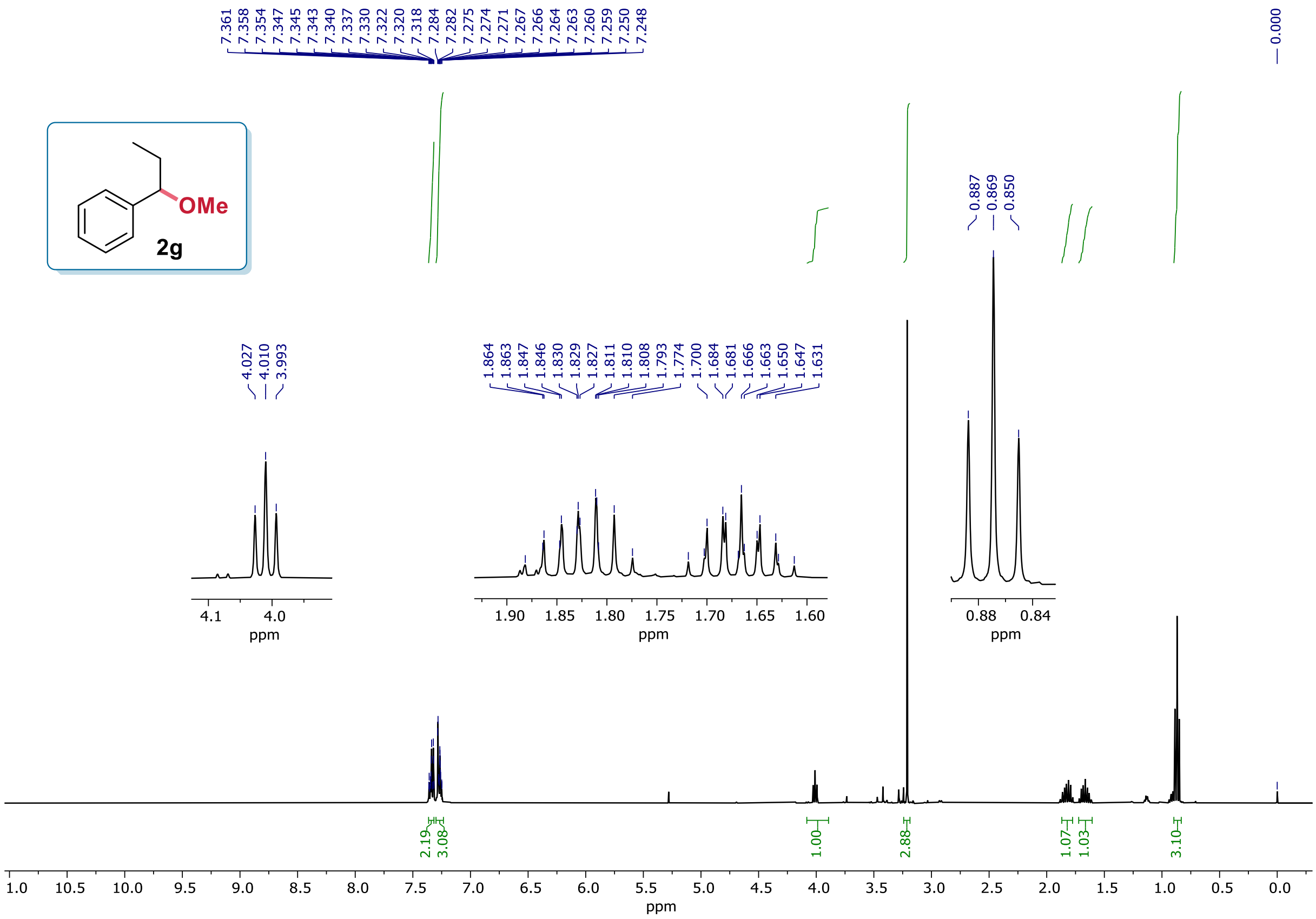

Figure. S13 ^{1}H NMR spectrum of **2g** in $CDCl_3$ (400 MHz)

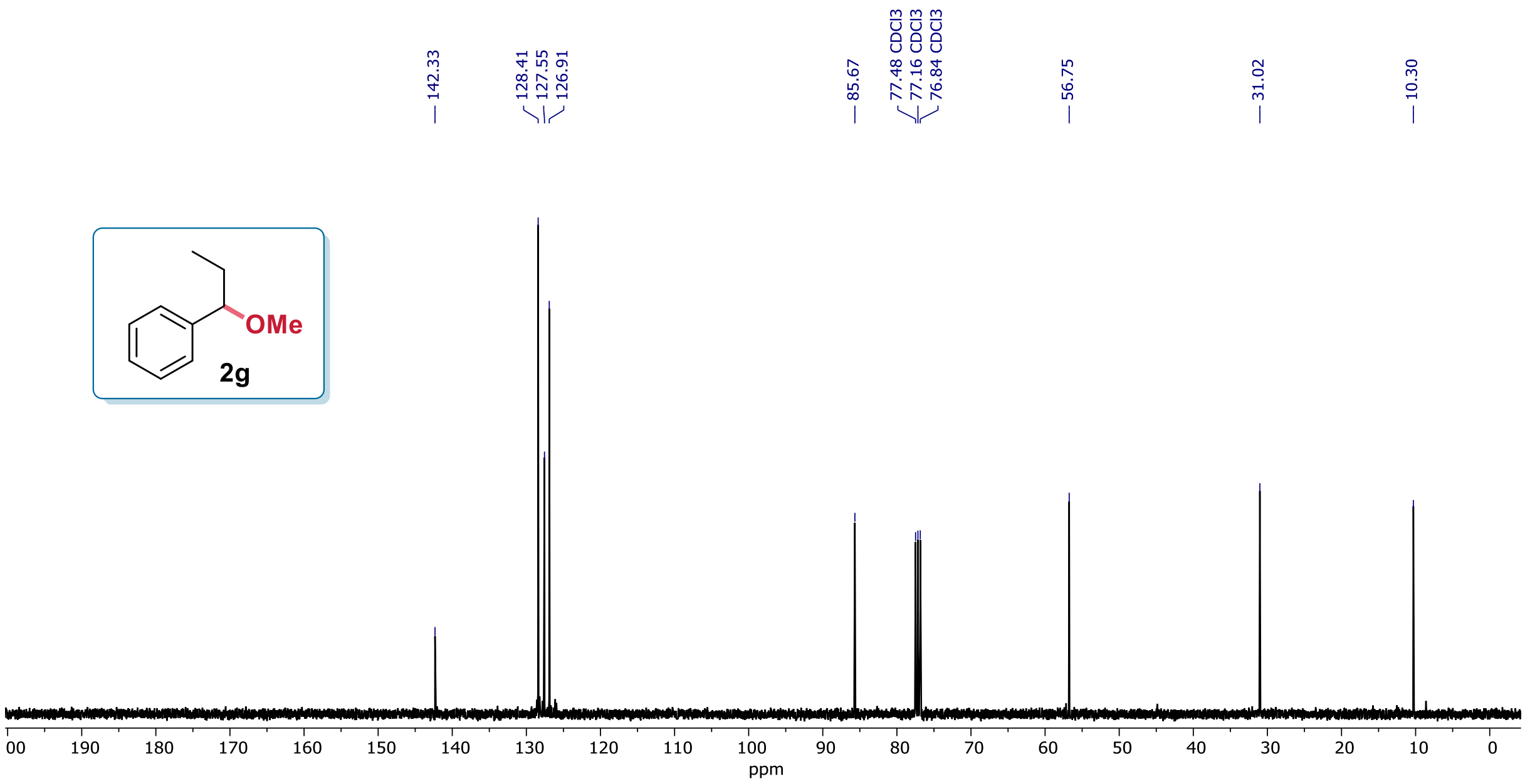

Figure. S14 $^{13}C\{^{1}H\}$ NMR spectrum of **2g** in $CDCl_3$ (101 MHz)

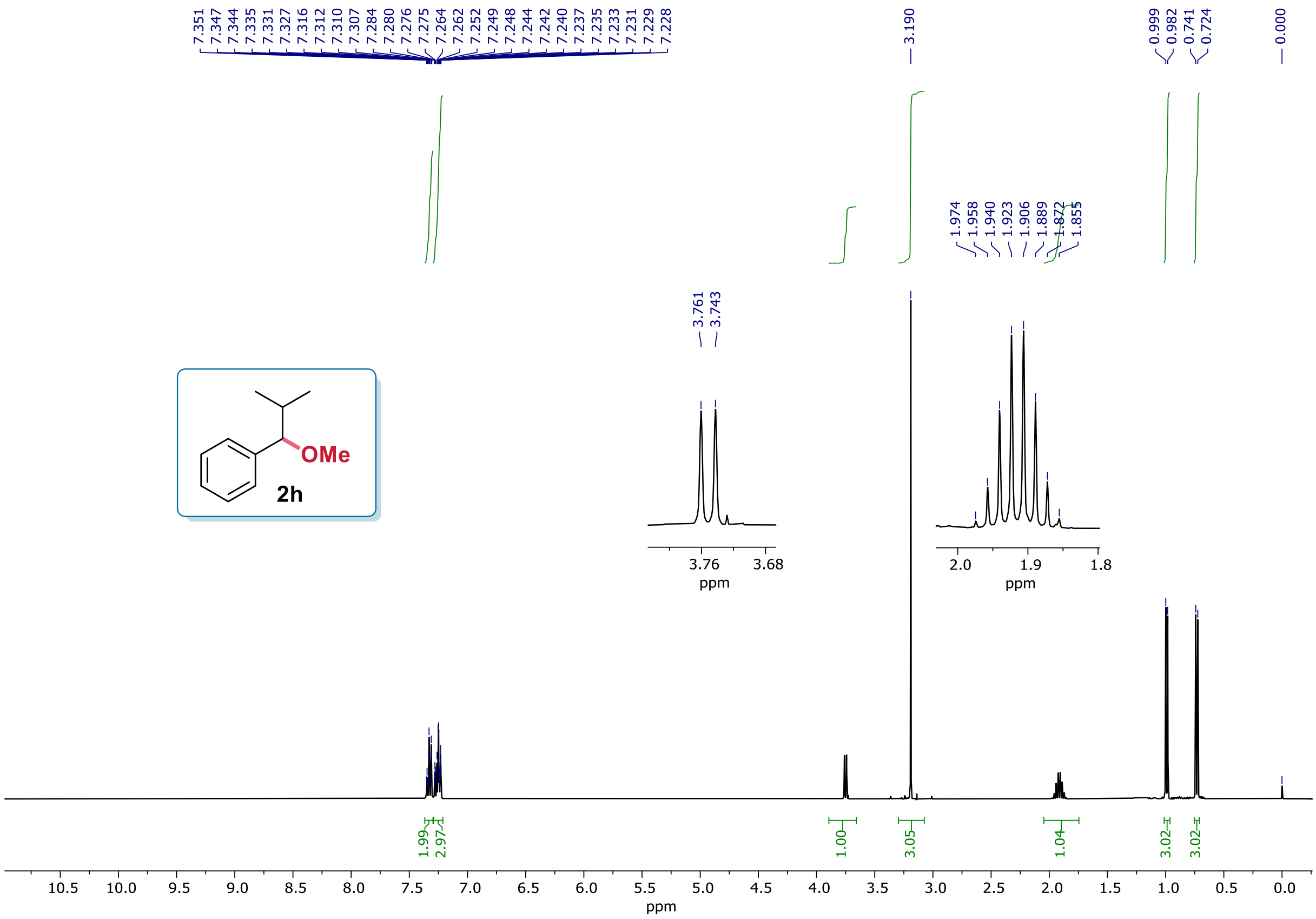

Figure. S15 ^{1}H NMR spectrum of **2h** in $CDCl_3$ (400 MHz)

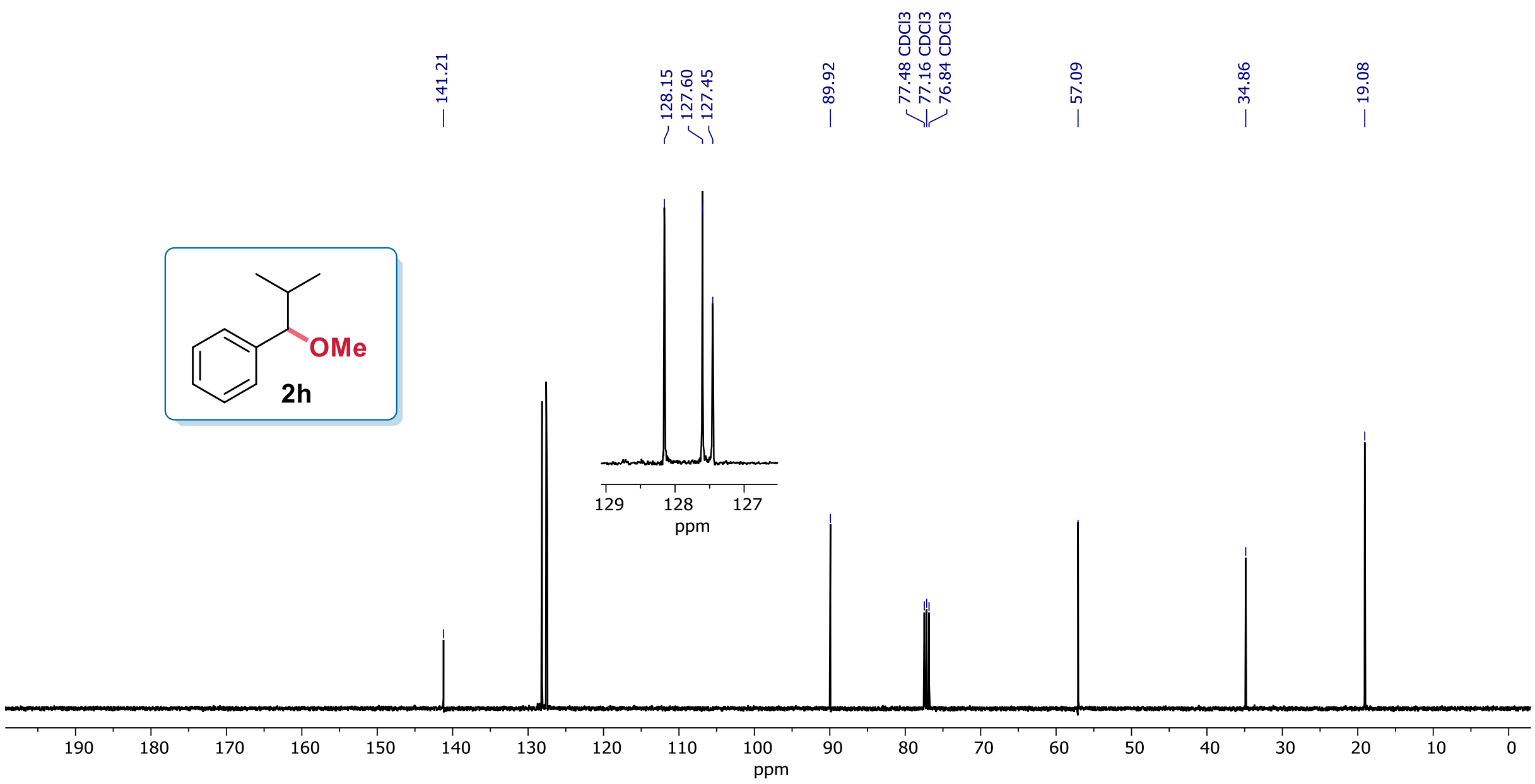

Figure. S16 $^{13}C\{^{1}H\}$ NMR spectrum of **2h** in $CDCl_3$ (101 MHz)

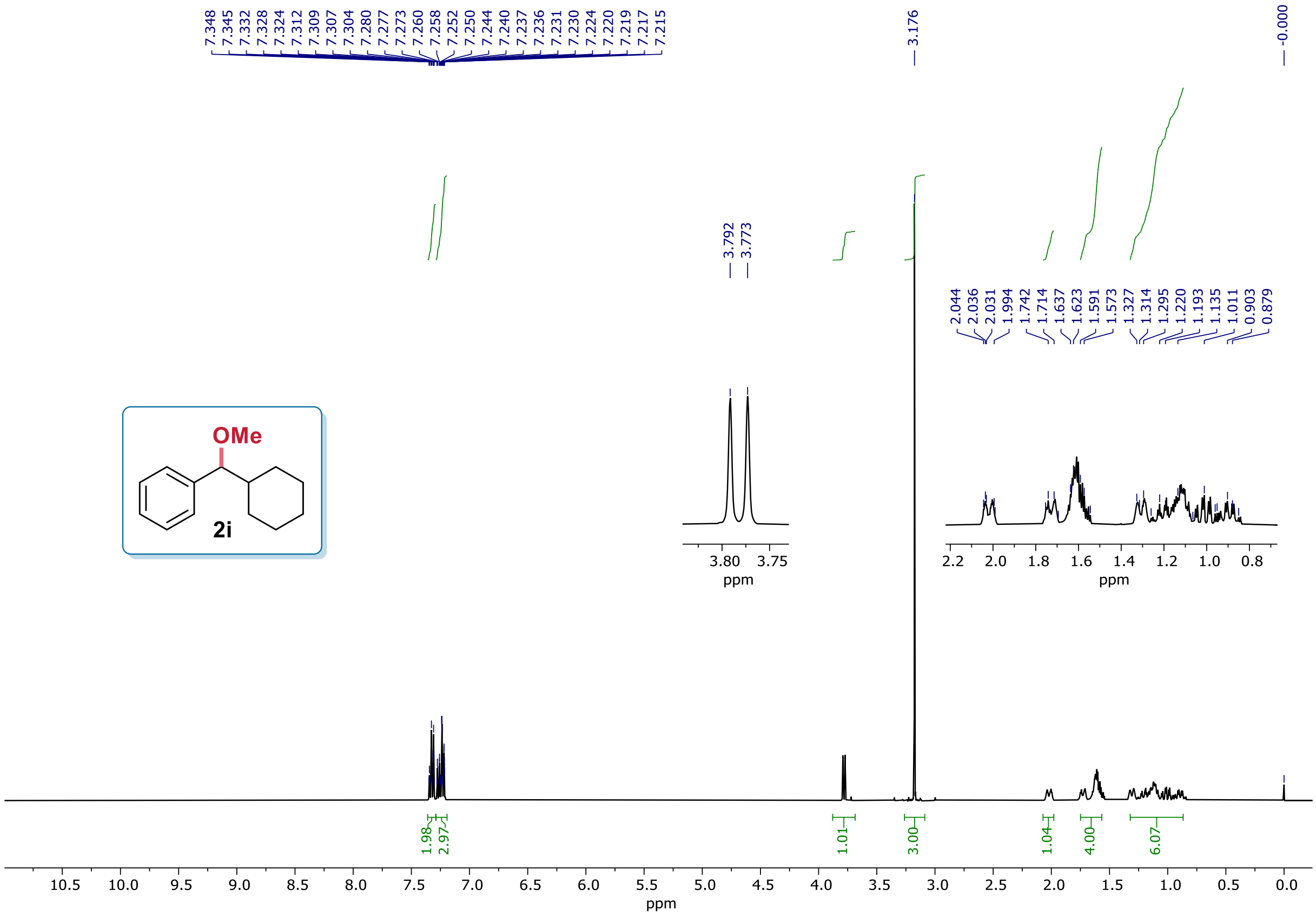

Figure. S17 ^{1}H NMR spectrum of **2i** in $CDCl_3$ (400 MHz)

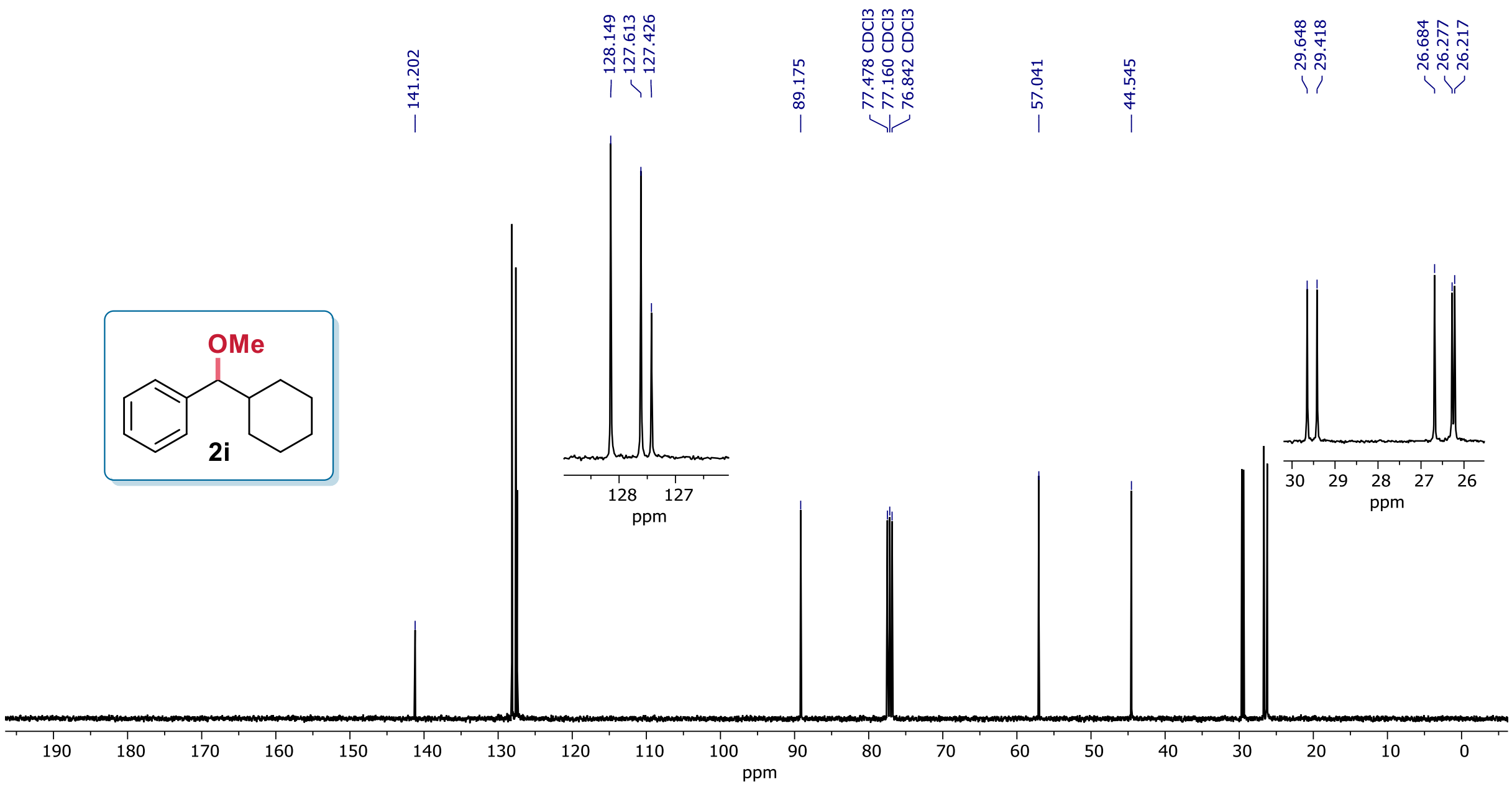

Figure. S18 $^{13}C\{^{1}H\}$ NMR spectrum of **2i** in $CDCl_3$ (101 MHz)

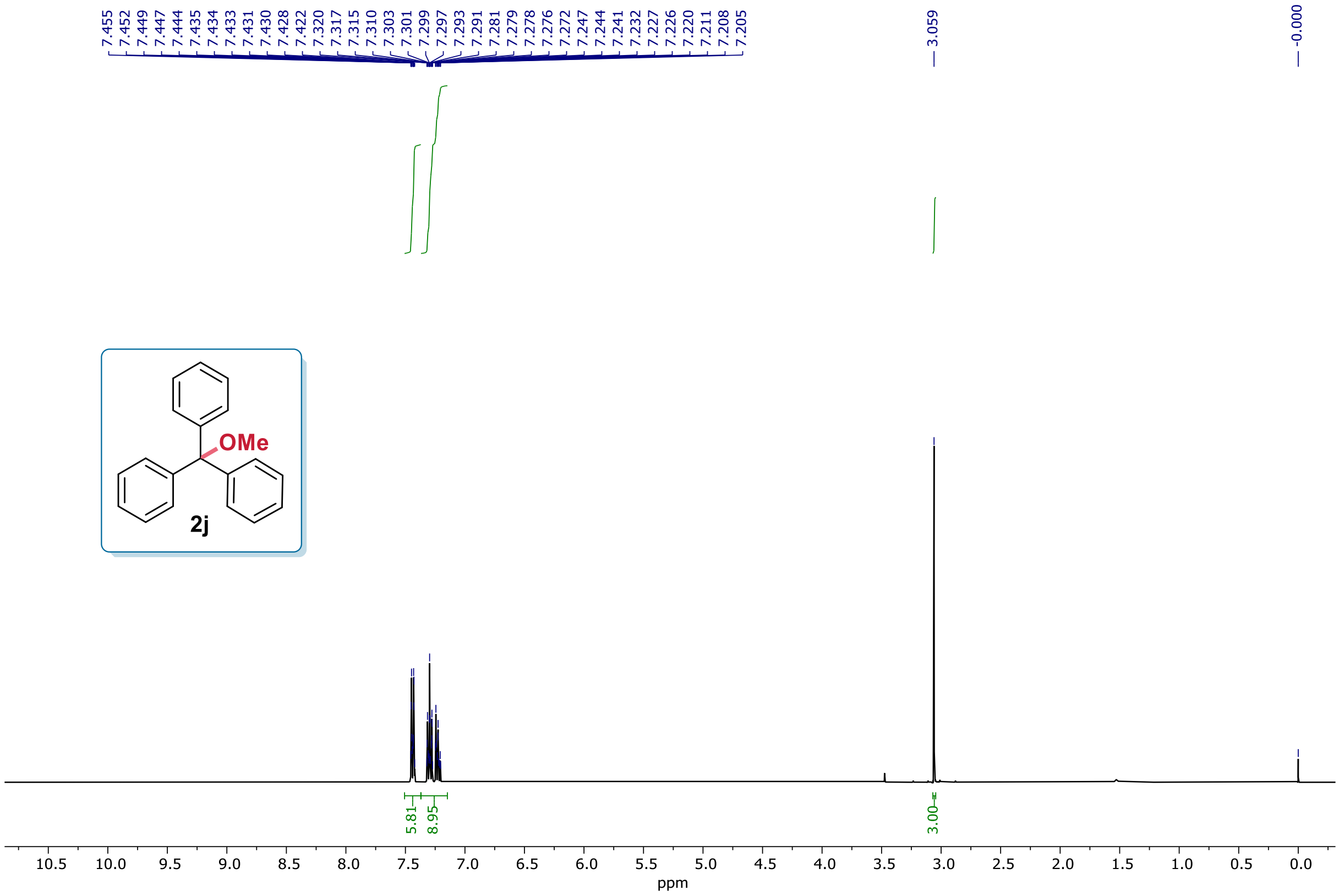

Figure. S19 ^{1}H NMR spectrum of **2j** in $CDCl_3$ (400 MHz)

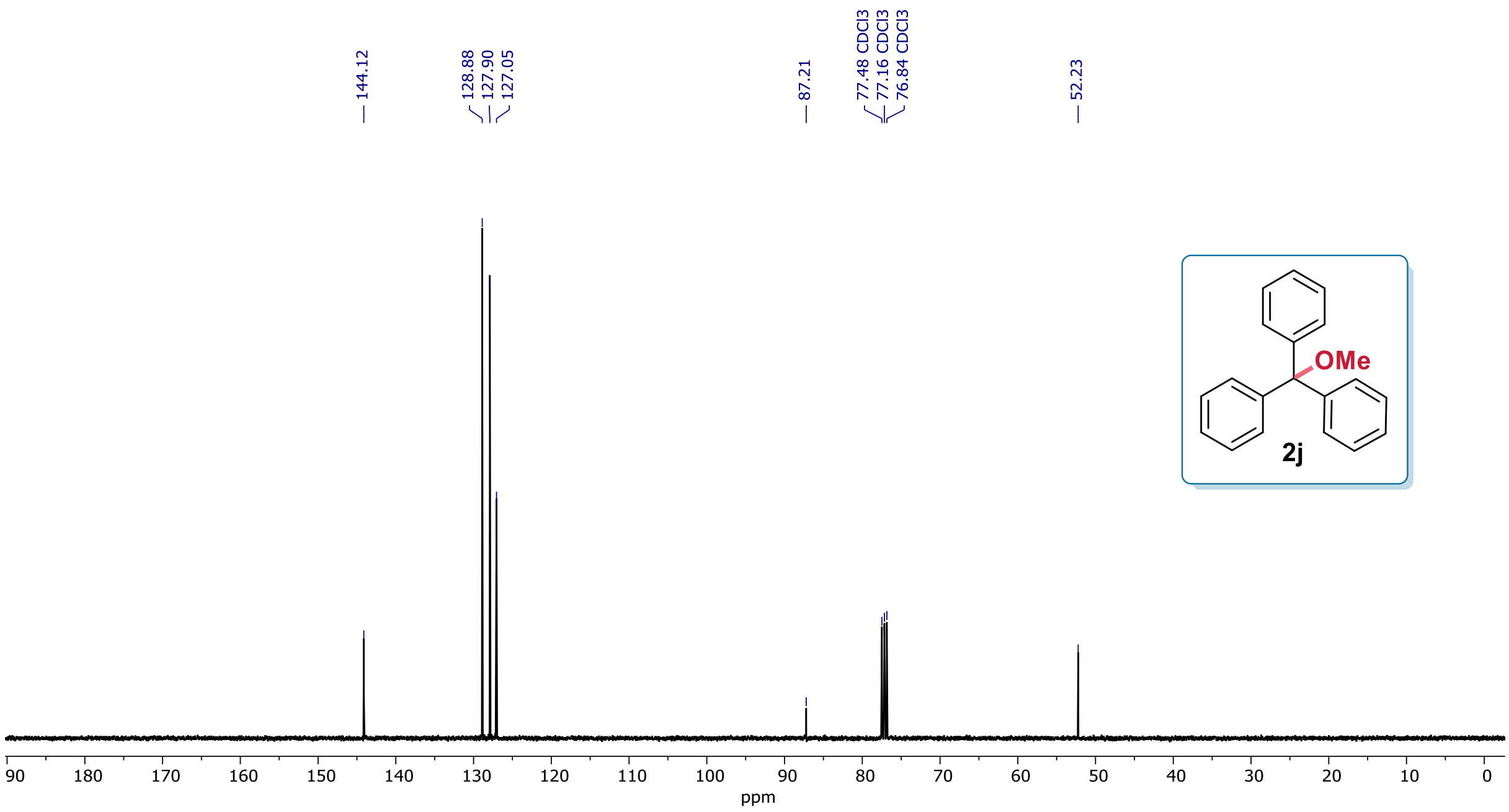

Figure. S20 $^{13}C\{^{1}H\}$ NMR spectrum of **2j** in $CDCl_3$ (101 MHz)

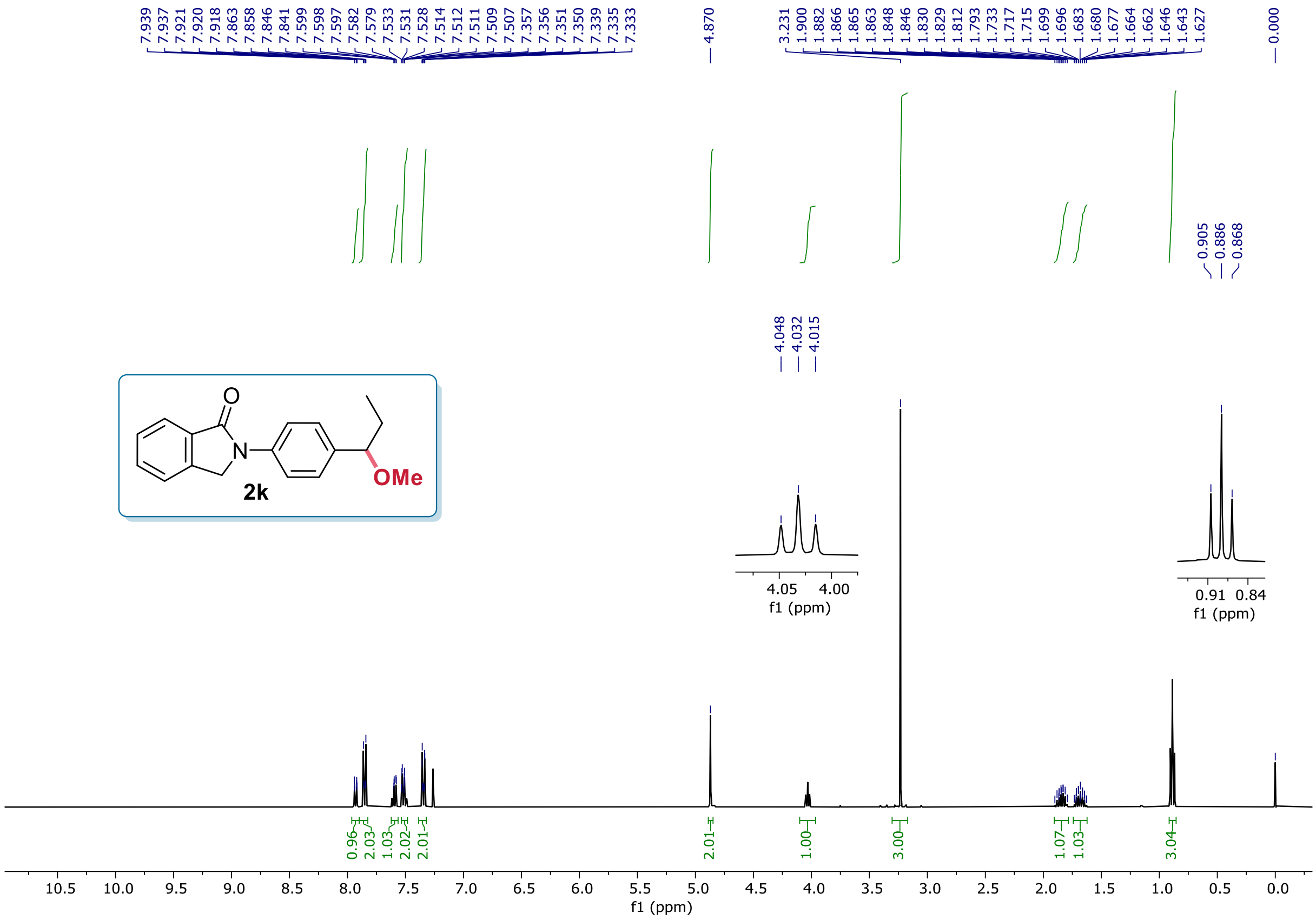

Figure. S21 ^{1}H NMR spectrum of **2k** in $CDCl_3$ (400 MHz)

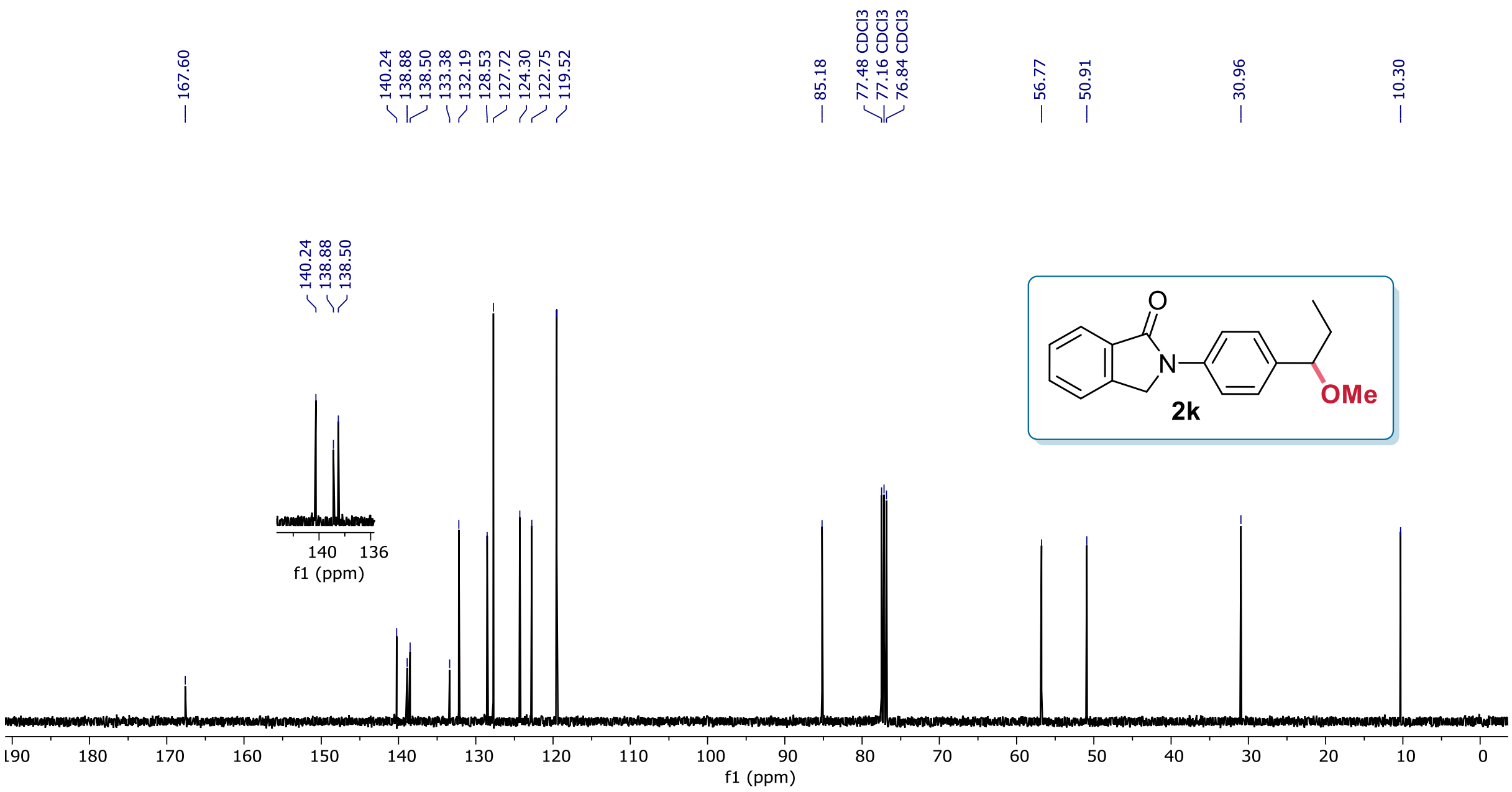

Figure. S22 $^{13}C\{^{1}H\}$ NMR spectrum of **2k** in $CDCl_3$ (101 MHz)

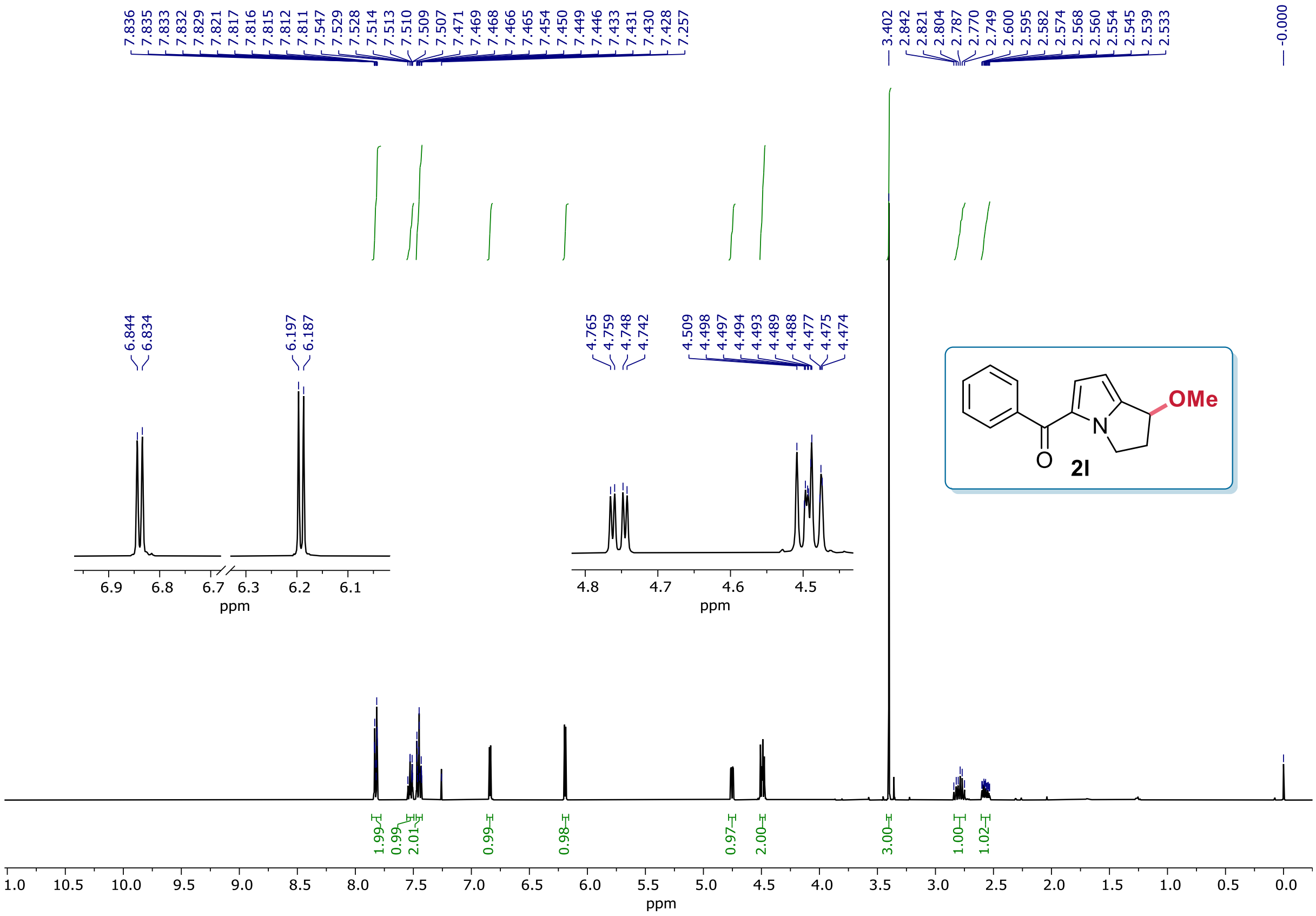

Figure. S23 ^{1}H NMR spectrum of **2l** in $CDCl_3$ (400 MHz)

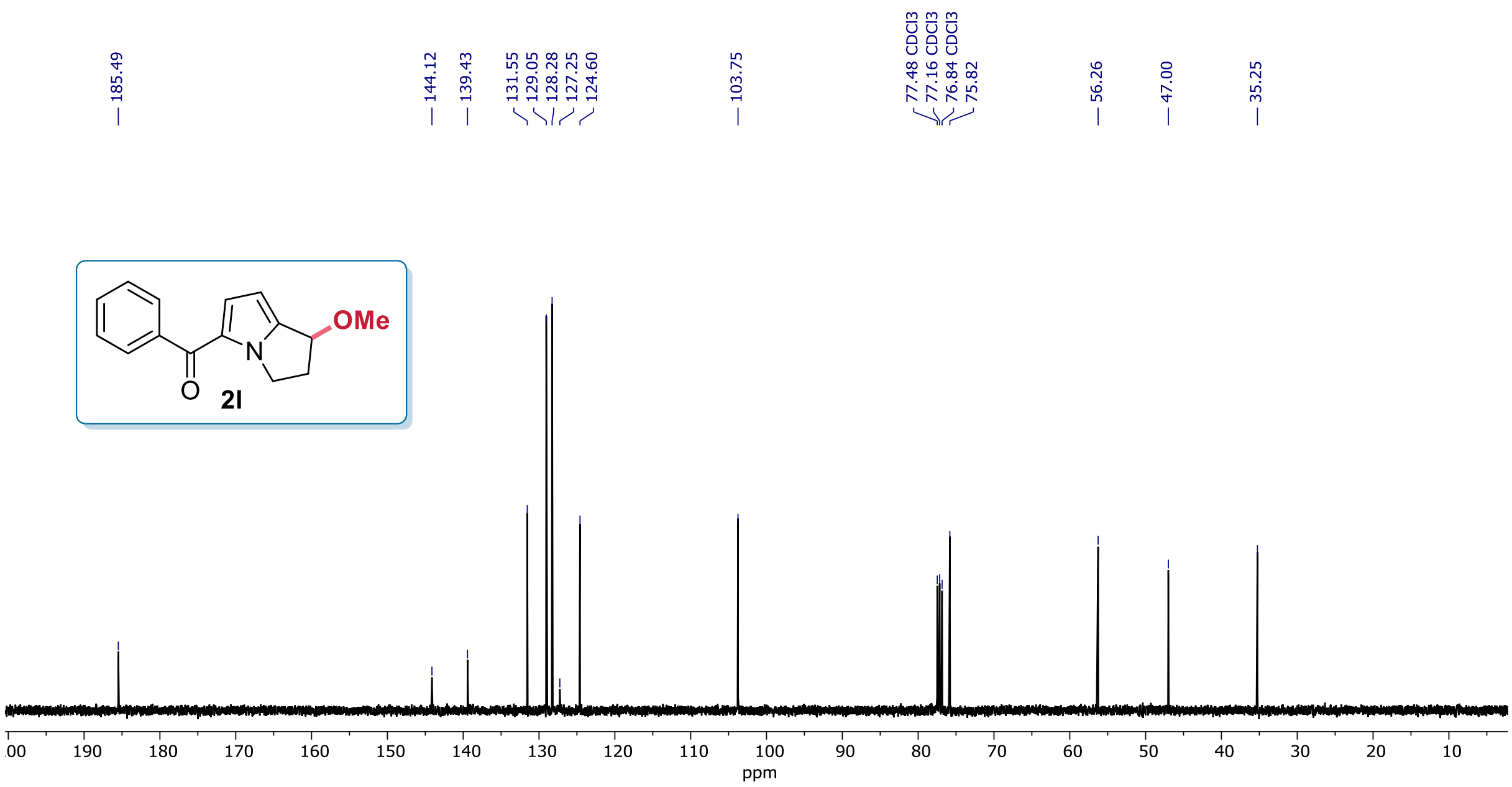

Figure. S24 $^{13}C\{^{1}H\}$ NMR spectrum of **2l** in $CDCl_3$ (101 MHz)

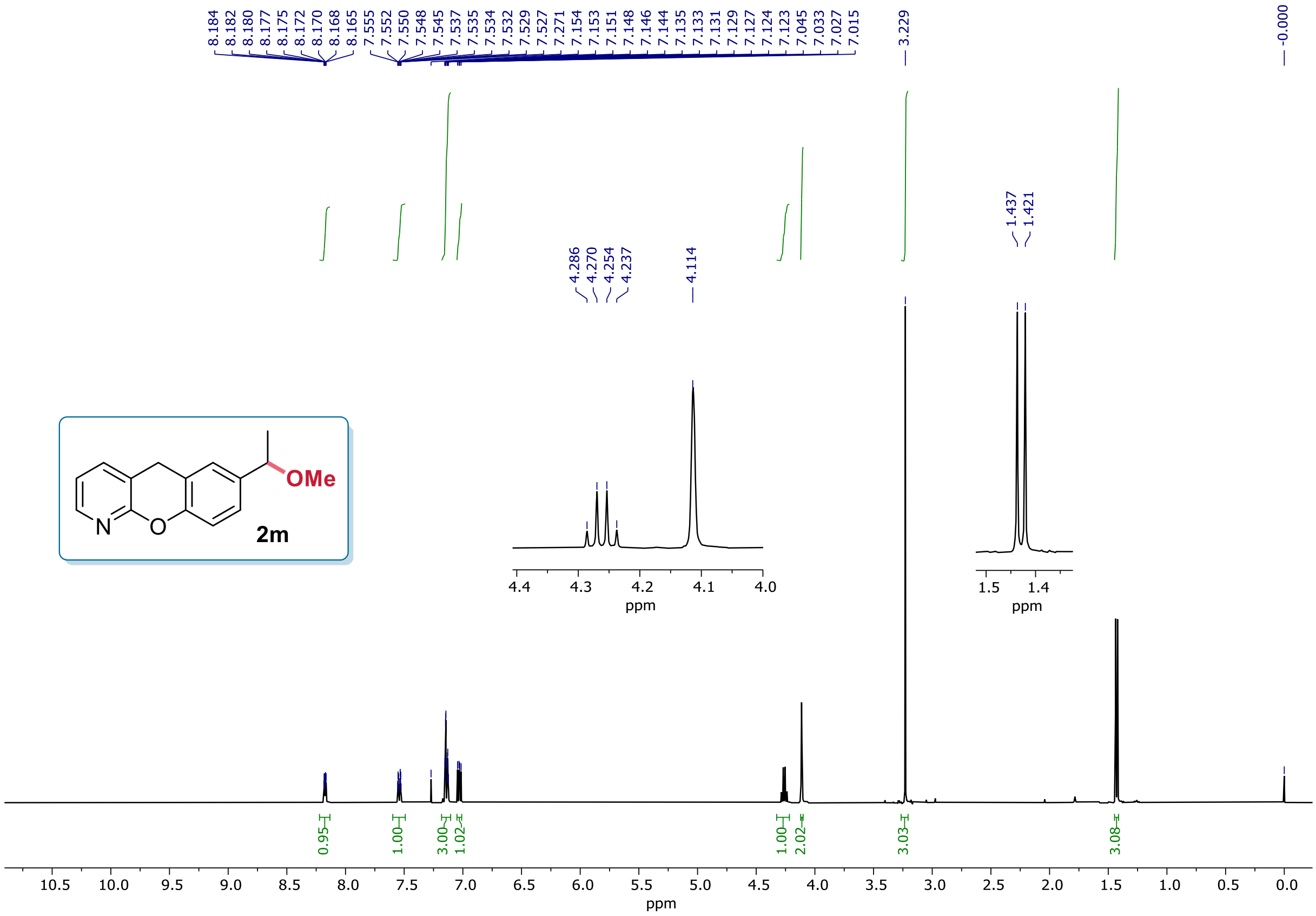

Figure. S25 ^{1}H NMR spectrum of **2m** in $CDCl_3$ (400 MHz)

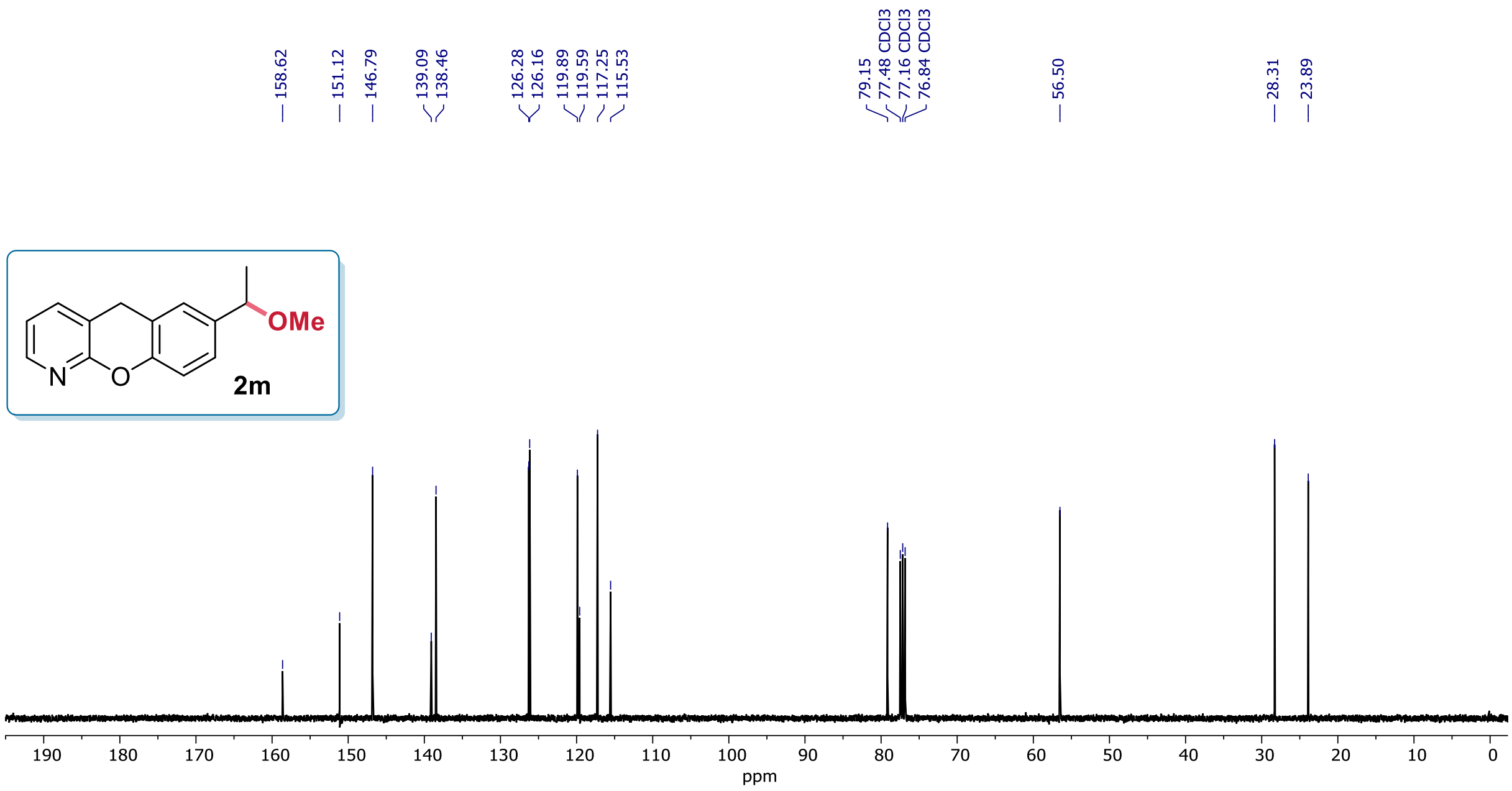

Figure. S26 $^{13}C\{^{1}H\}$ NMR spectrum of **2m** in $CDCl_3$ (101 MHz)

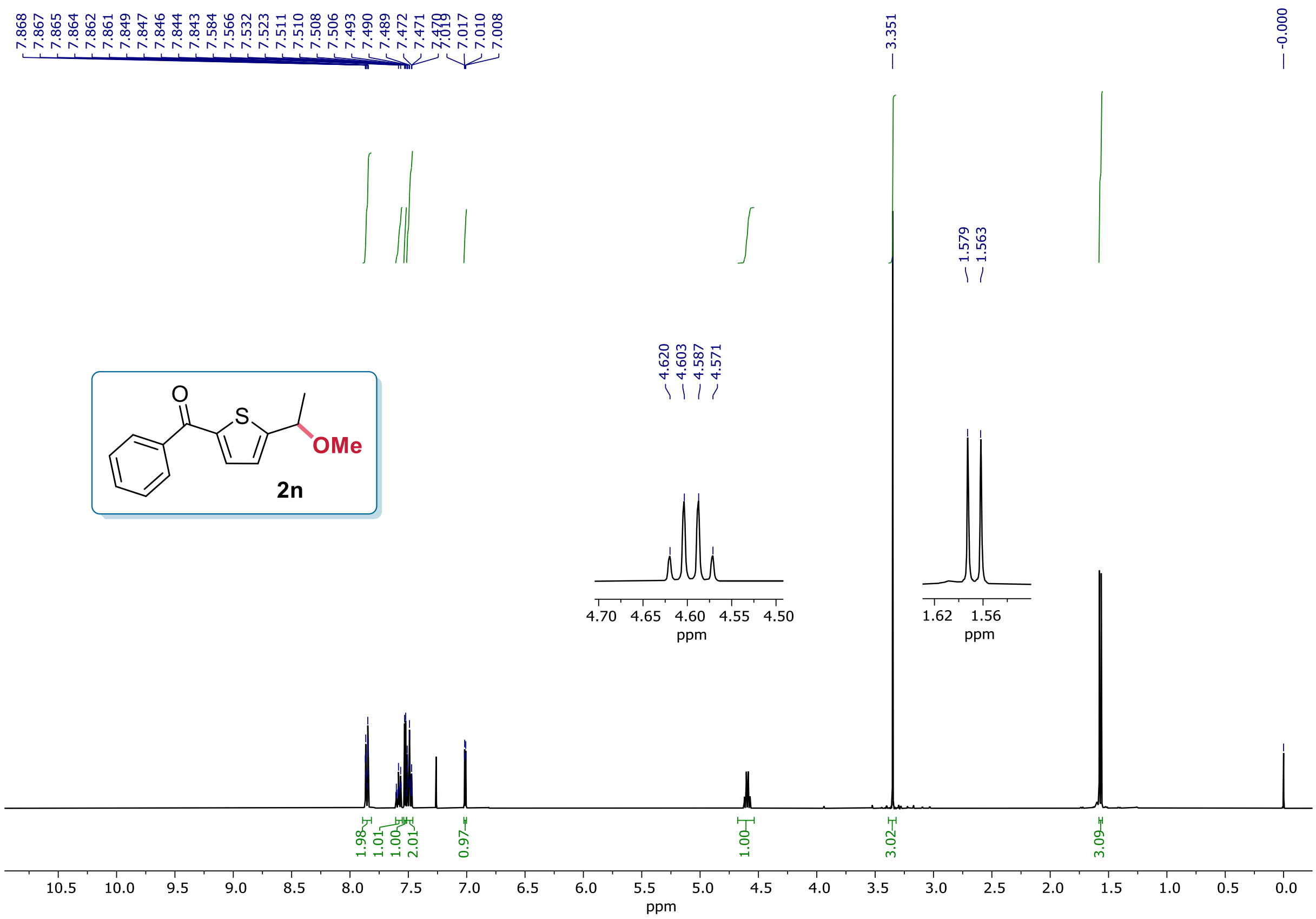

Figure. S27 ^{1}H NMR spectrum of **2n** in $CDCl_3$ (400 MHz)

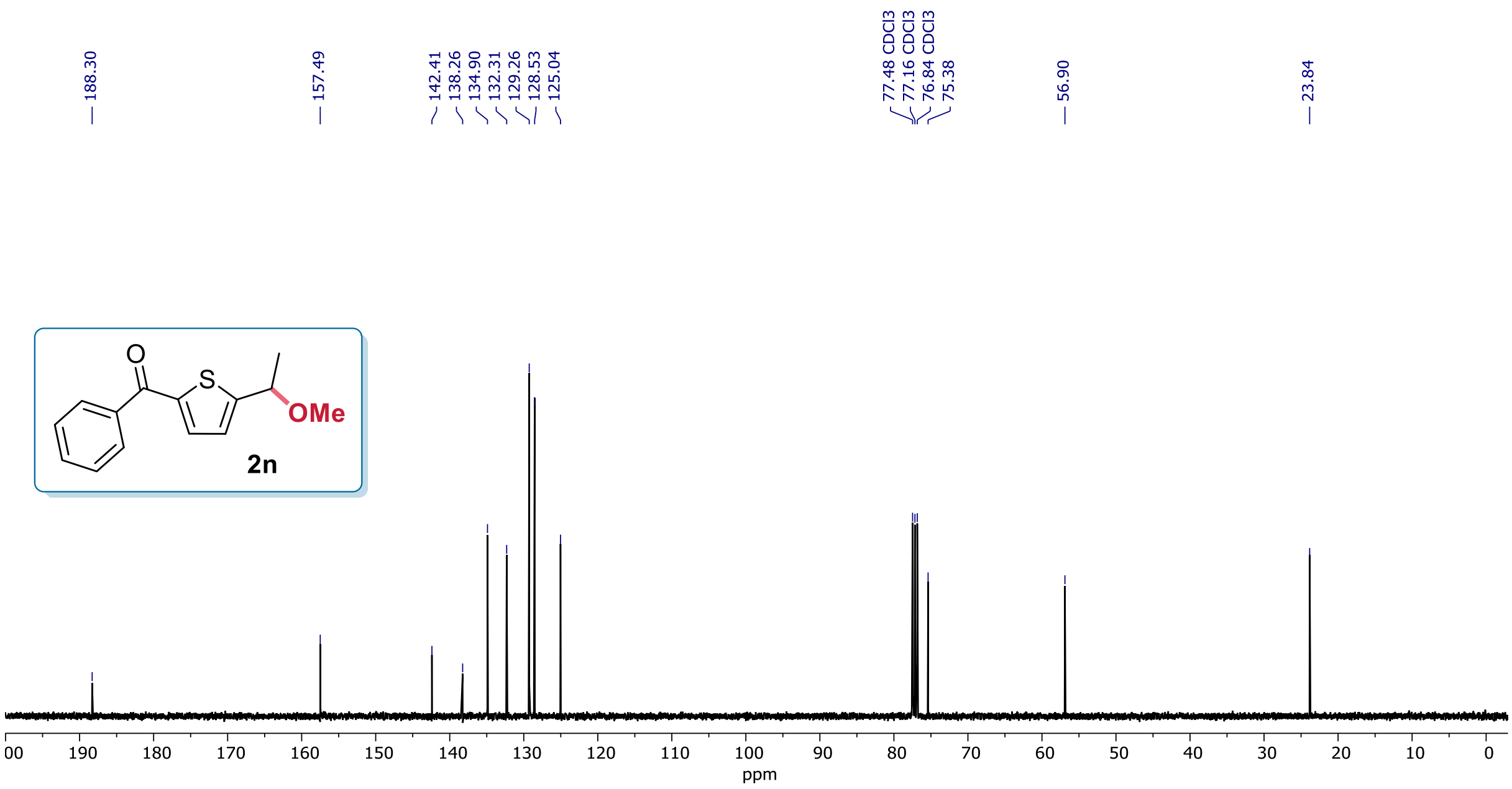

Figure. S28 $^{13}C\{^{1}H\}$ NMR spectrum of **2n** in $CDCl_3$ (101 MHz)

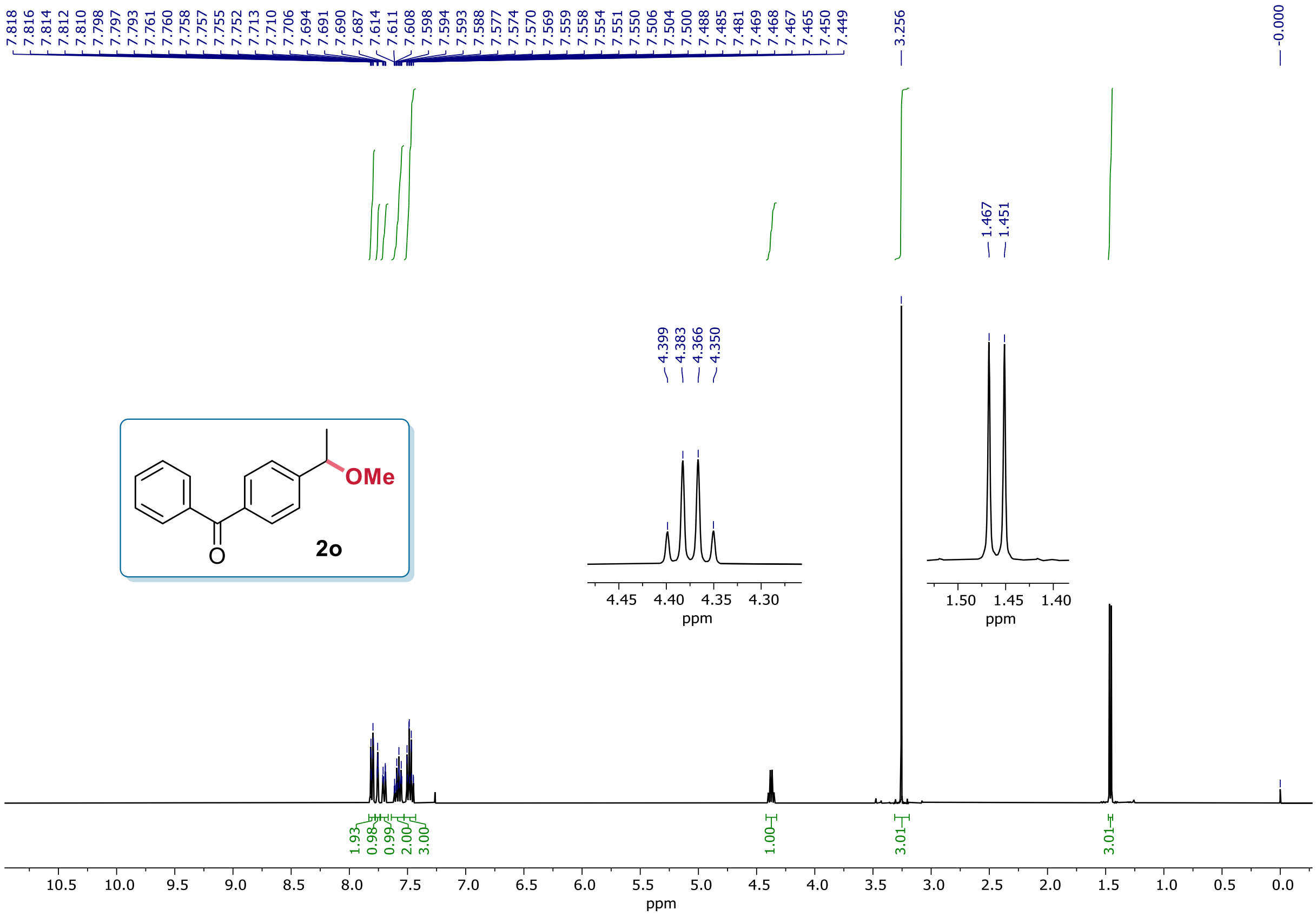

Figure. S29 ^{1}H NMR spectrum of **2o** in $CDCl_3$ (400 MHz)

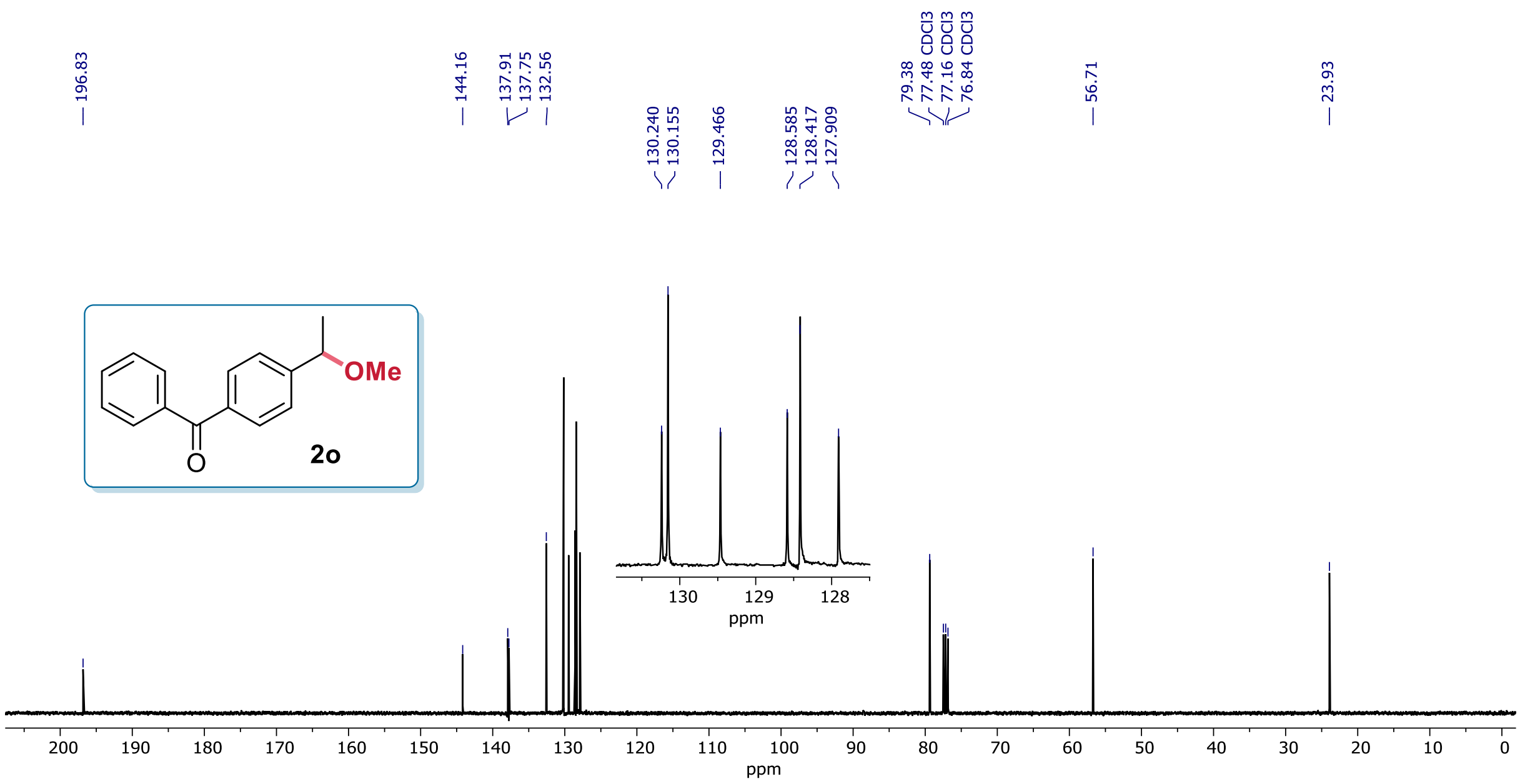

Figure. S30 $^{13}C\{^{1}H\}$ NMR spectrum of **2o** in $CDCl_3$ (101 MHz)

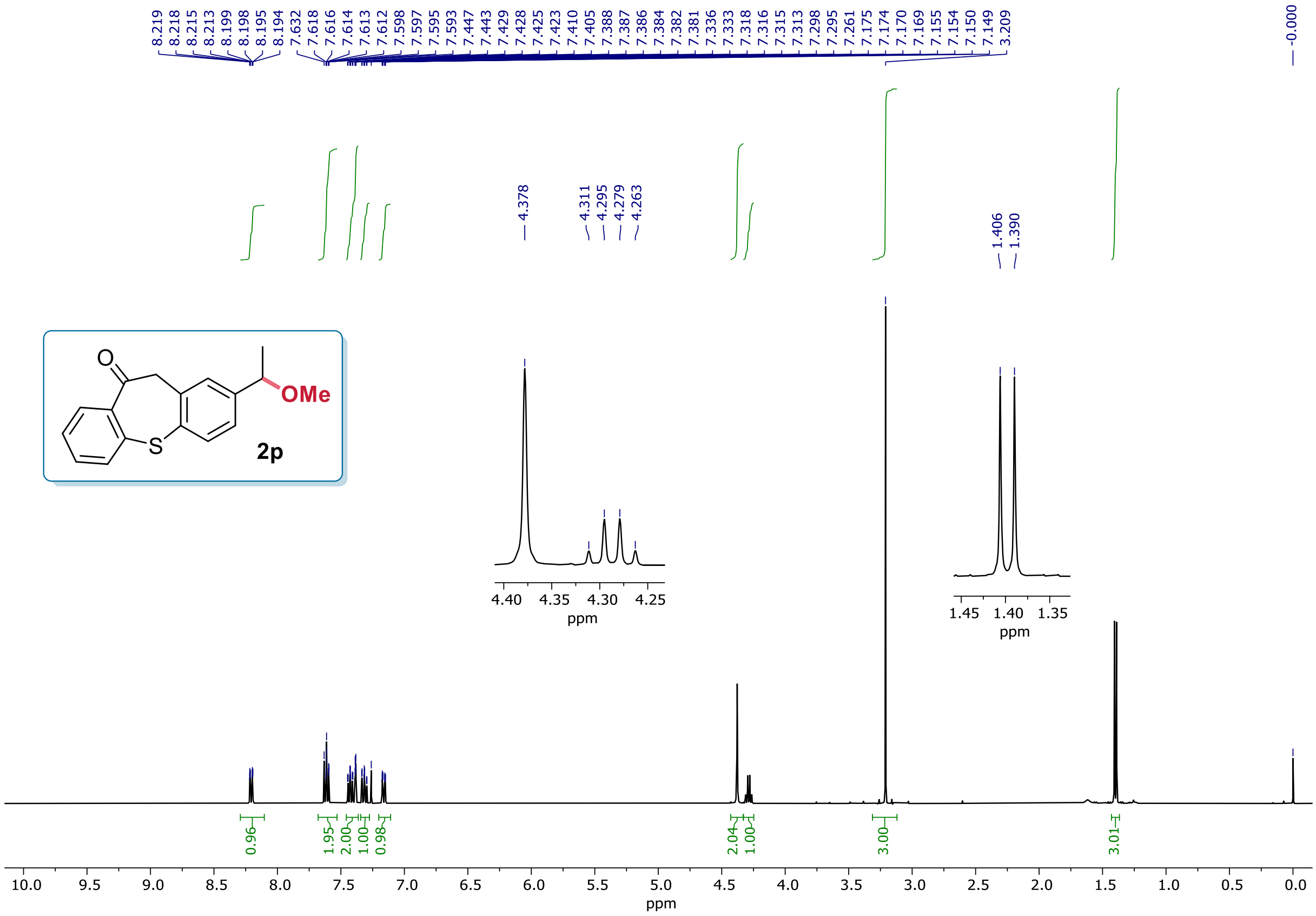

Figure. S31 ^{1}H NMR spectrum of **2p** in $CDCl_3$ (400 MHz)

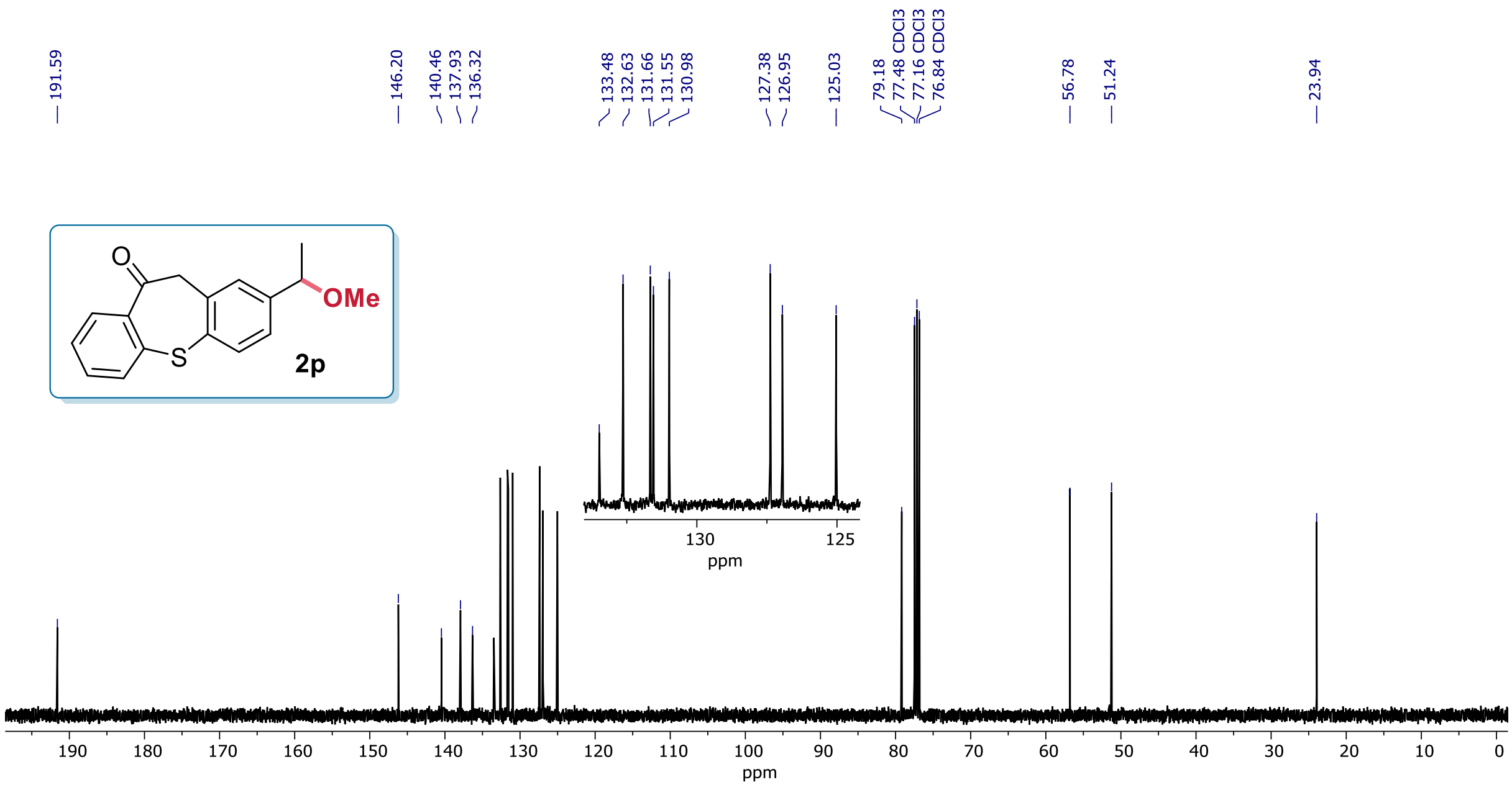

Figure. S32 ^{13}C{^{1}H} NMR spectrum of **2p** in $CDCl_3$ (101 MHz)

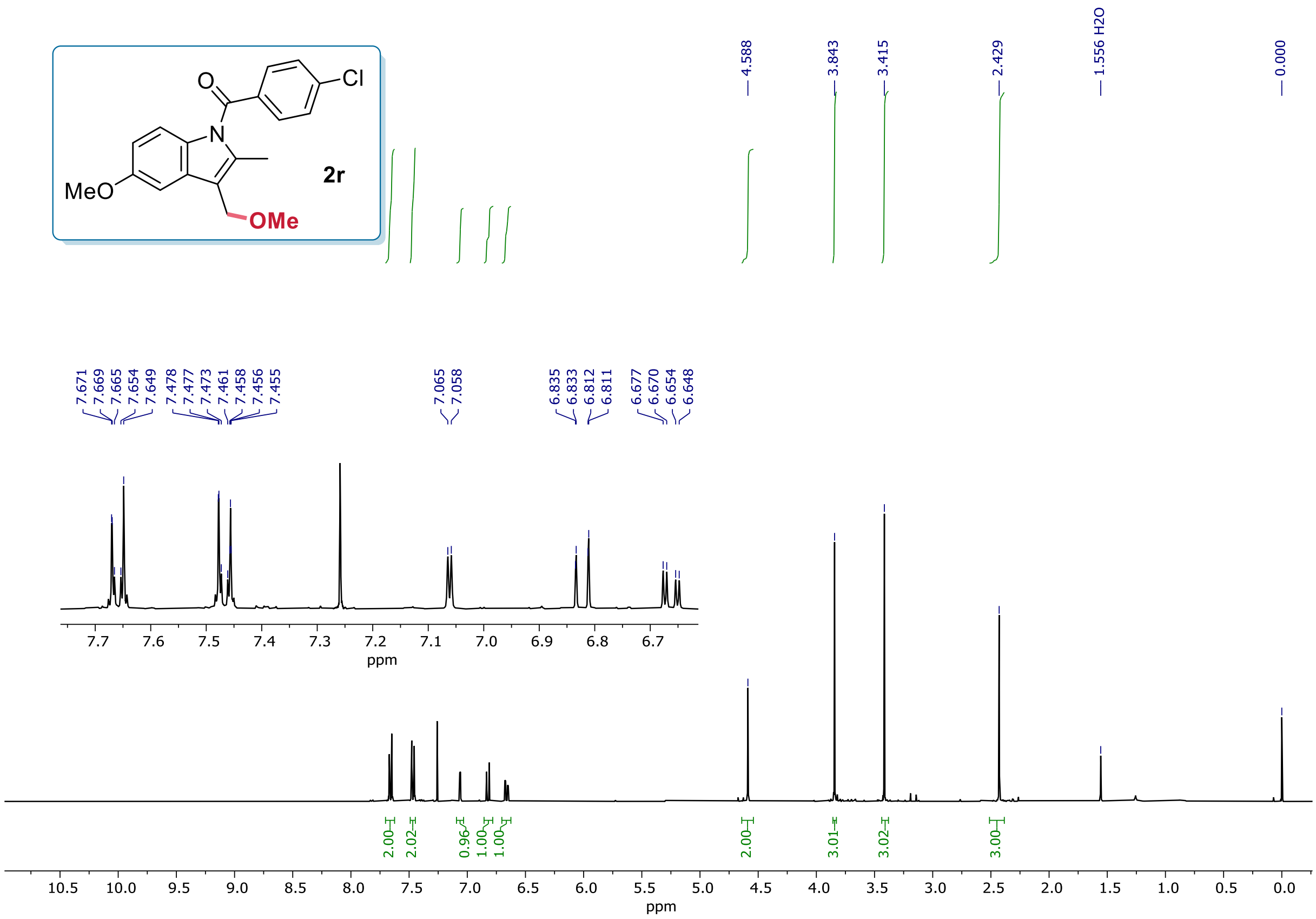

Figure. S33 ^{1}H NMR spectrum of **2r** in $CDCl_3$ (400 MHz)

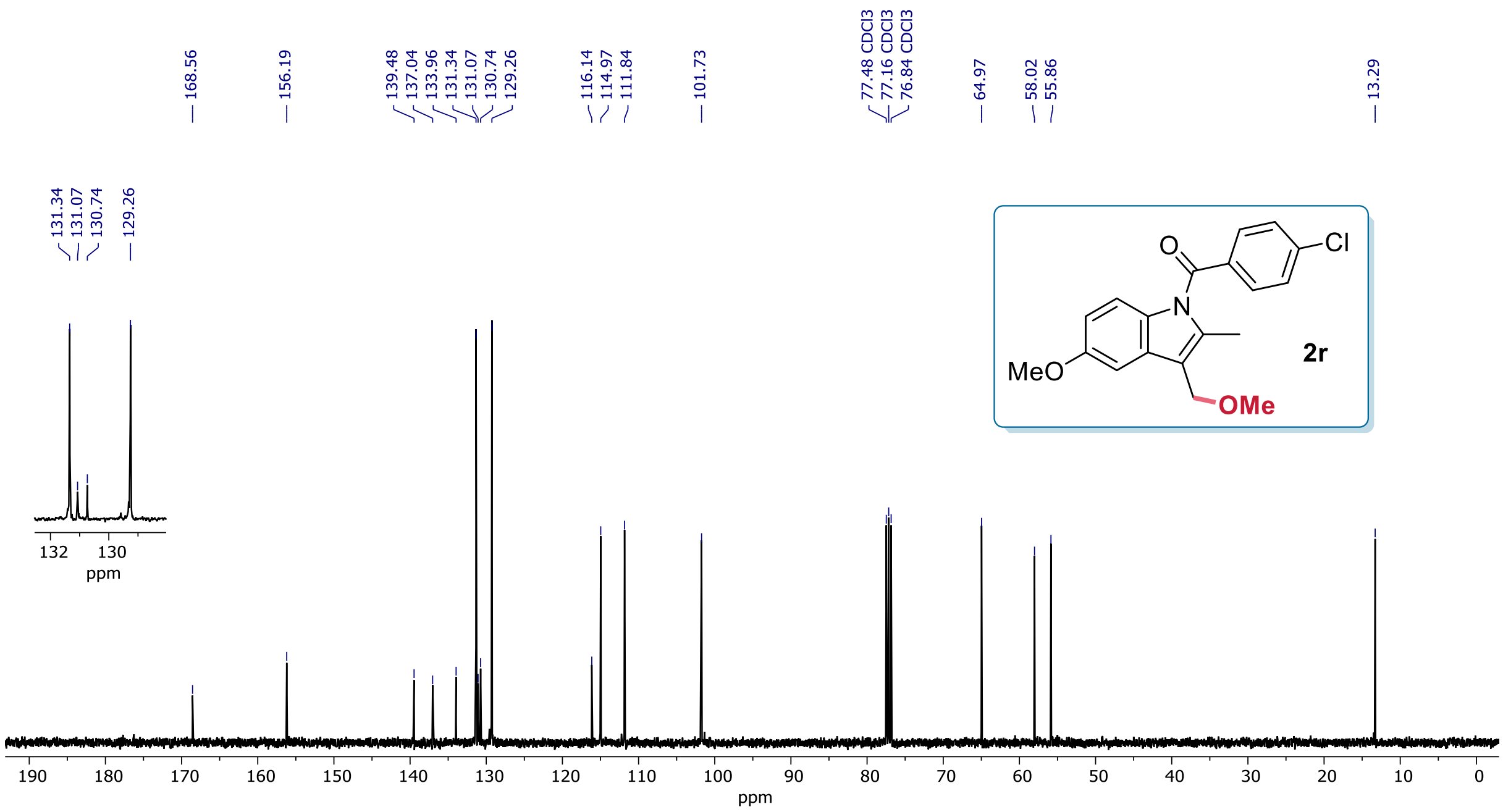

Figure. S34 $^{13}C\{^{1}H\}$ NMR spectrum of **2r** in $CDCl_3$ (101 MHz)

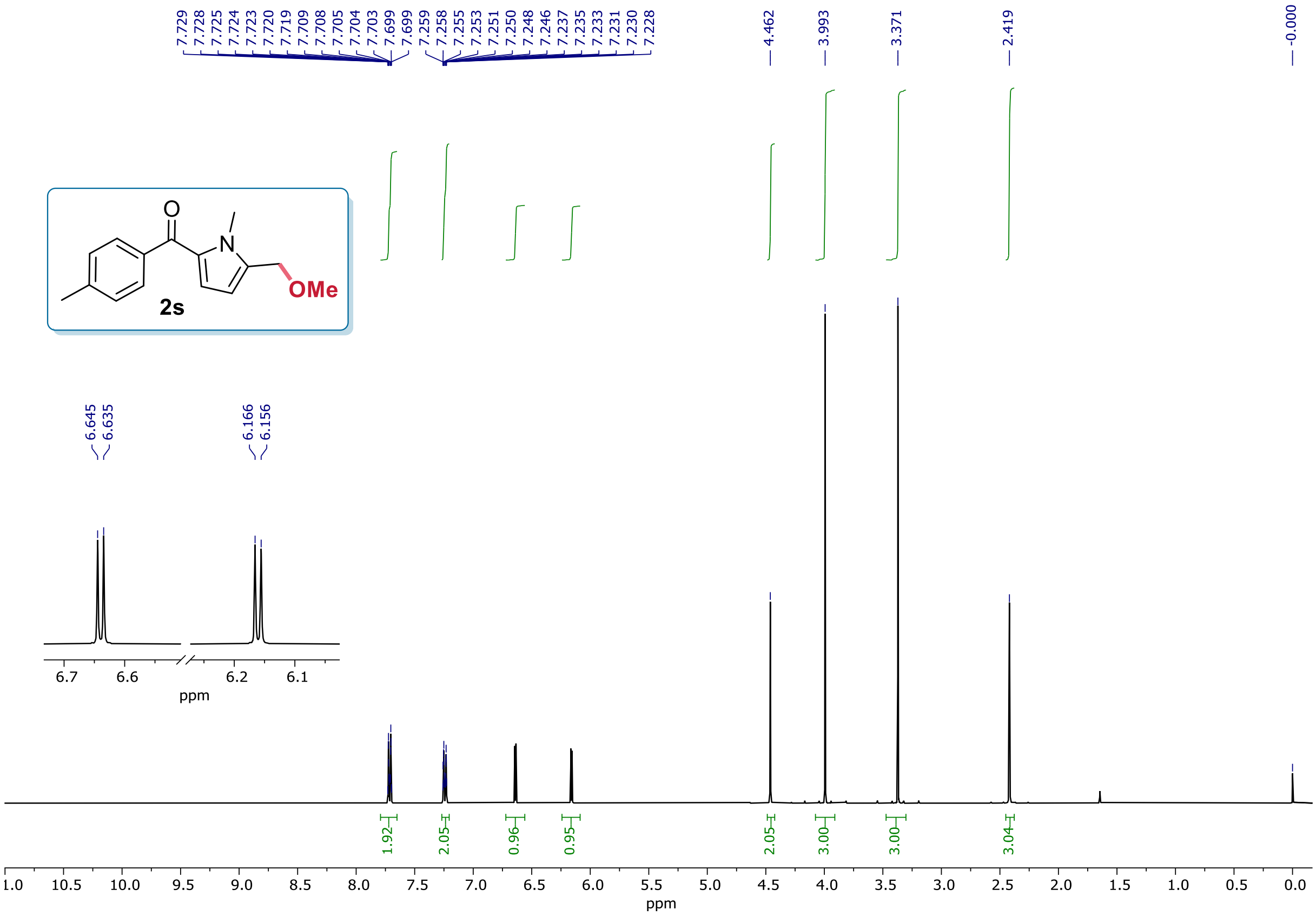

Figure. S35 ^{1}H NMR spectrum of **2s** in $CDCl_3$ (400 MHz)

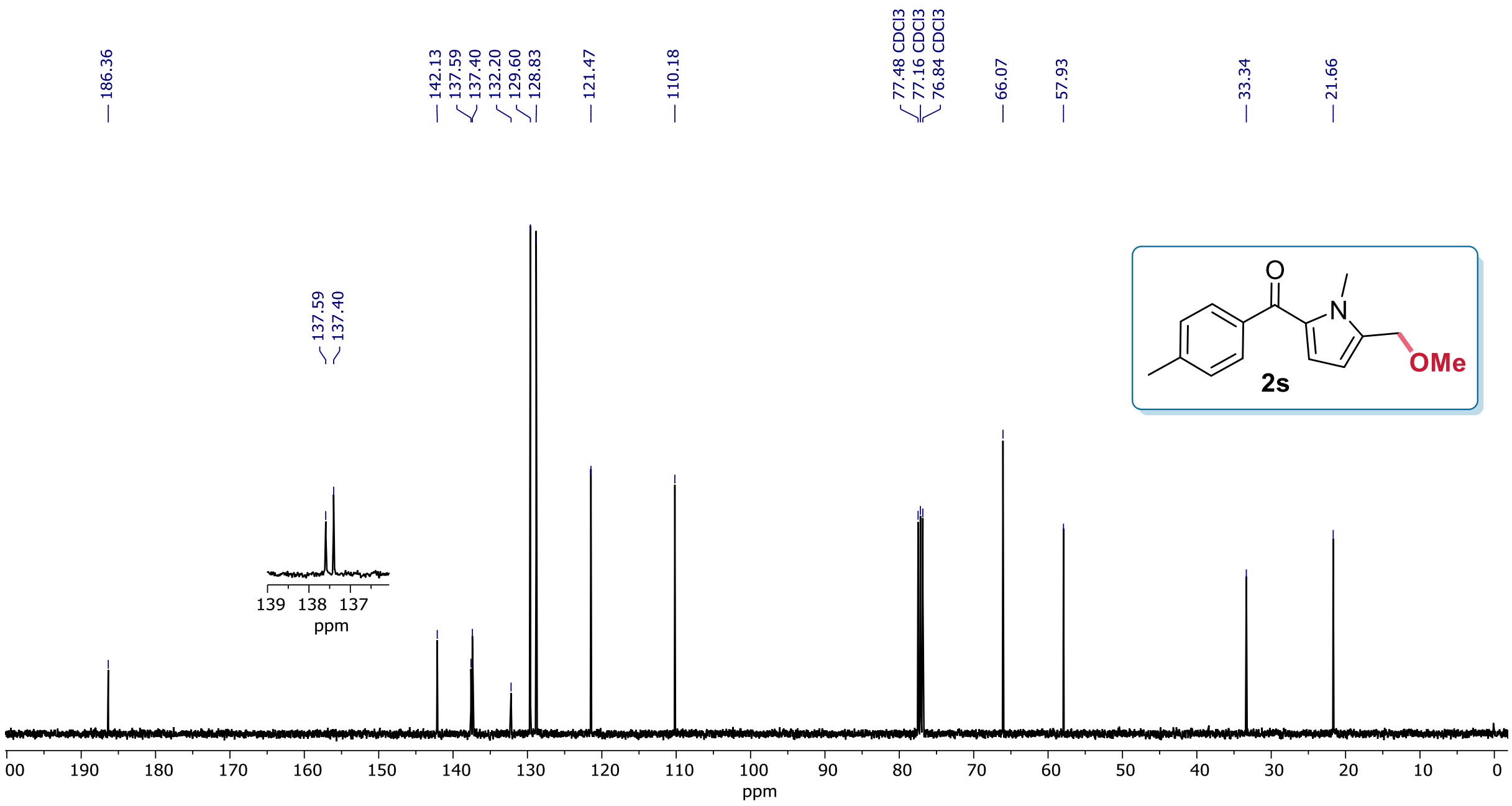

Figure. S36 $^{13}C\{^{1}H\}$ NMR spectrum of **2s** in $CDCl_3$ (101 MHz)

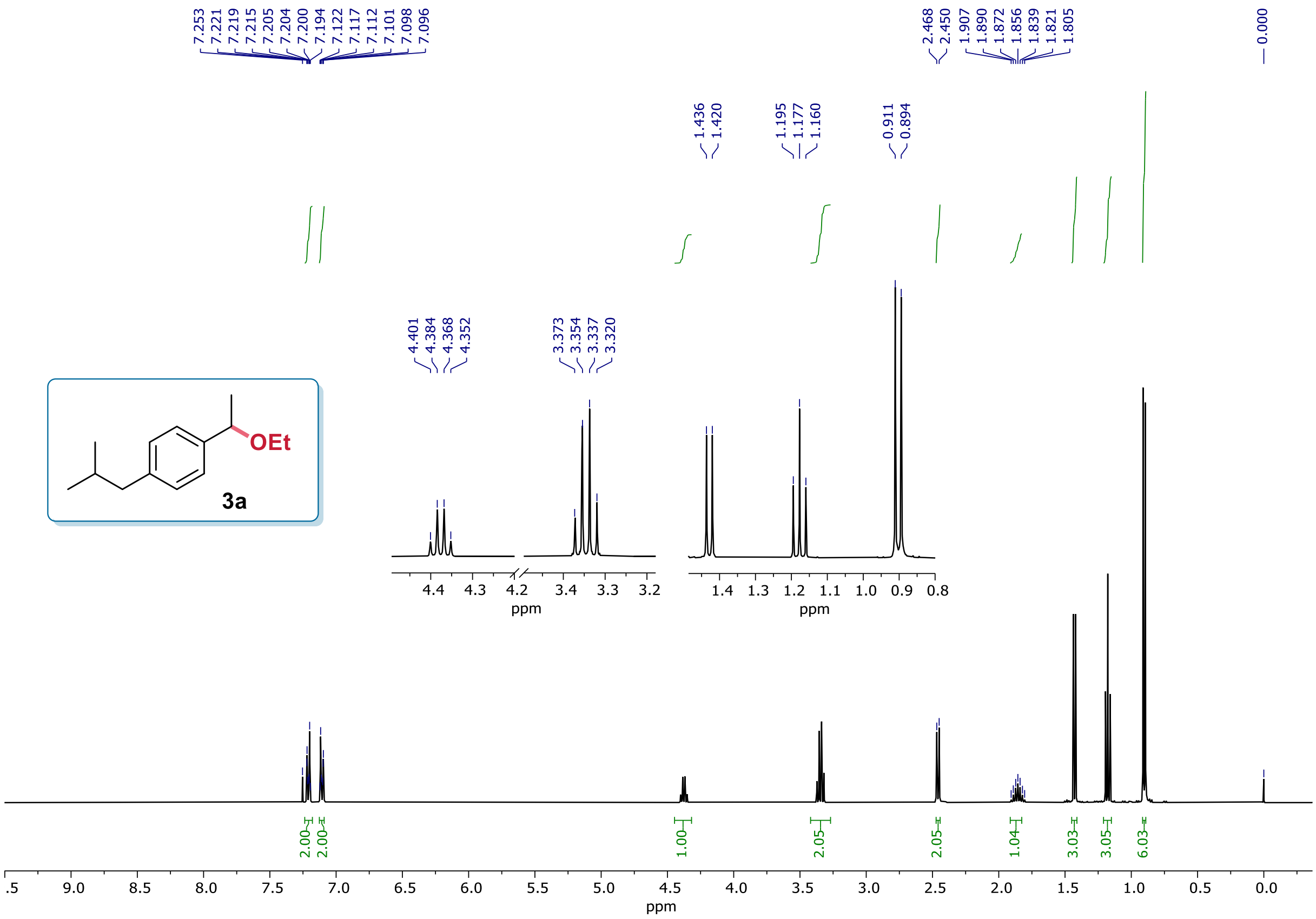

Figure. S37 ^{1}H NMR spectrum of **3a** in $CDCl_3$ (400 MHz)

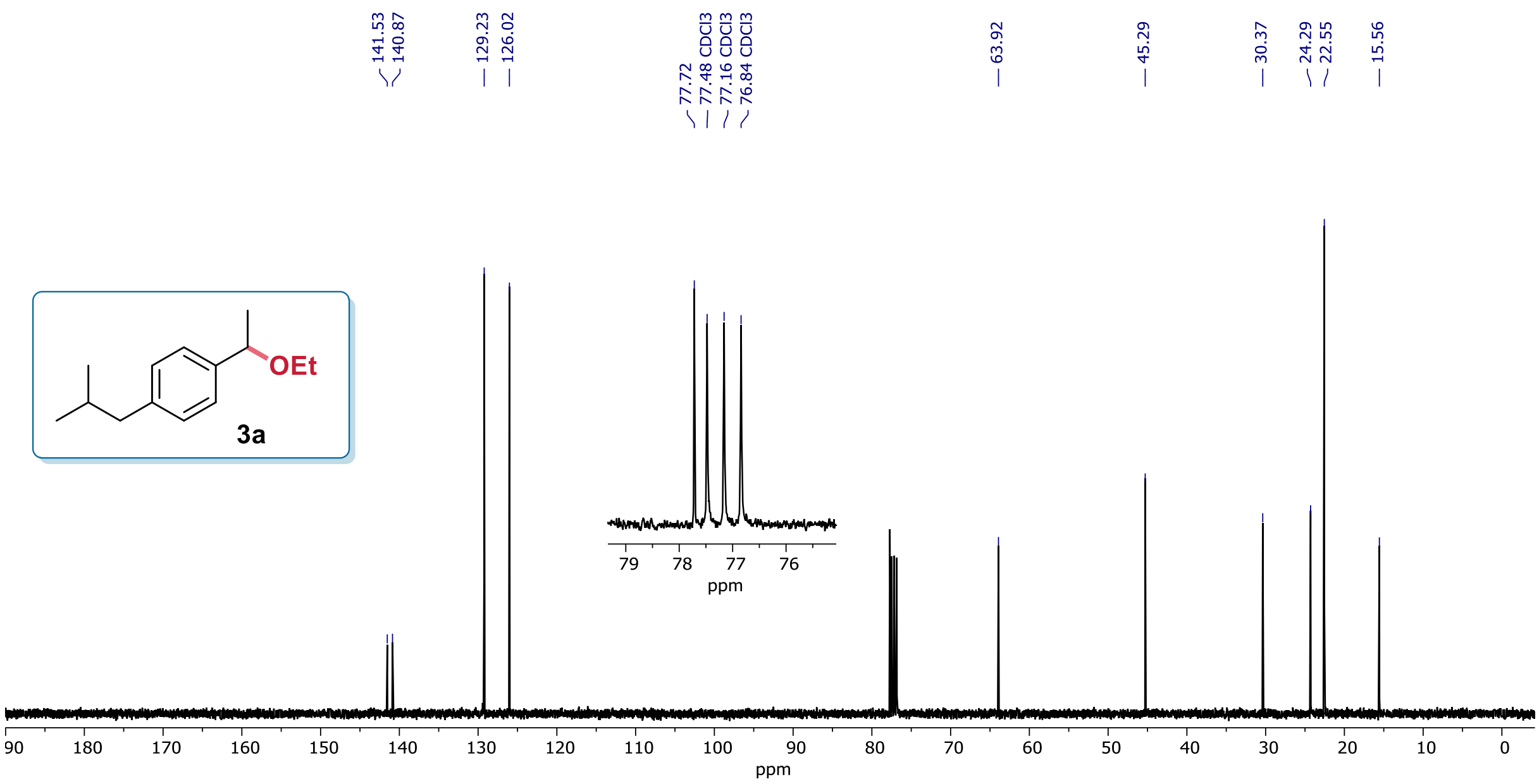

Figure. S38 $^{13}C\{^{1}H\}$ NMR spectrum of **3a** in $CDCl_3$ (101 MHz)

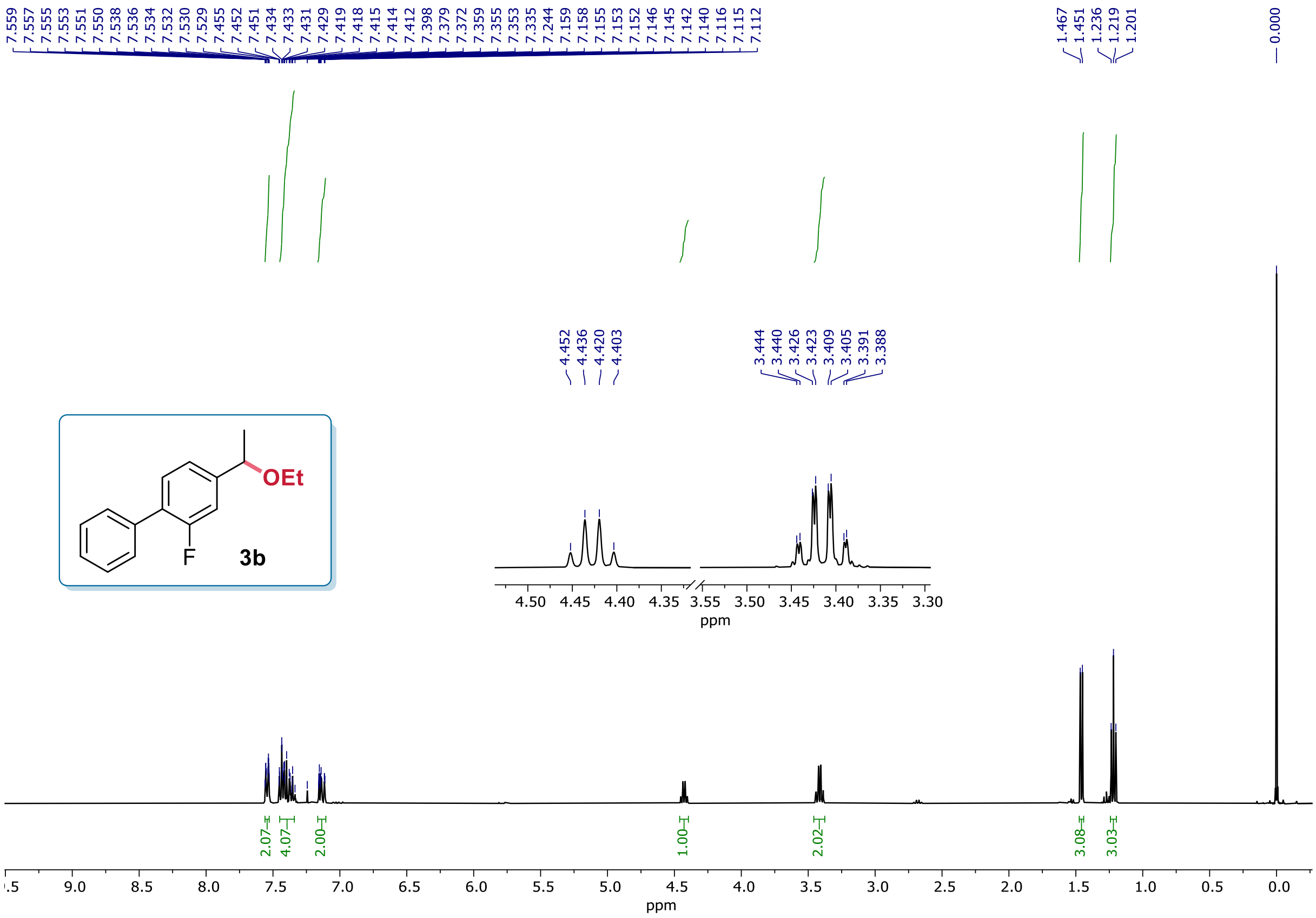

Figure. S39 ^{1}H NMR spectrum of **3b** in $CDCl_3$ (400 MHz)

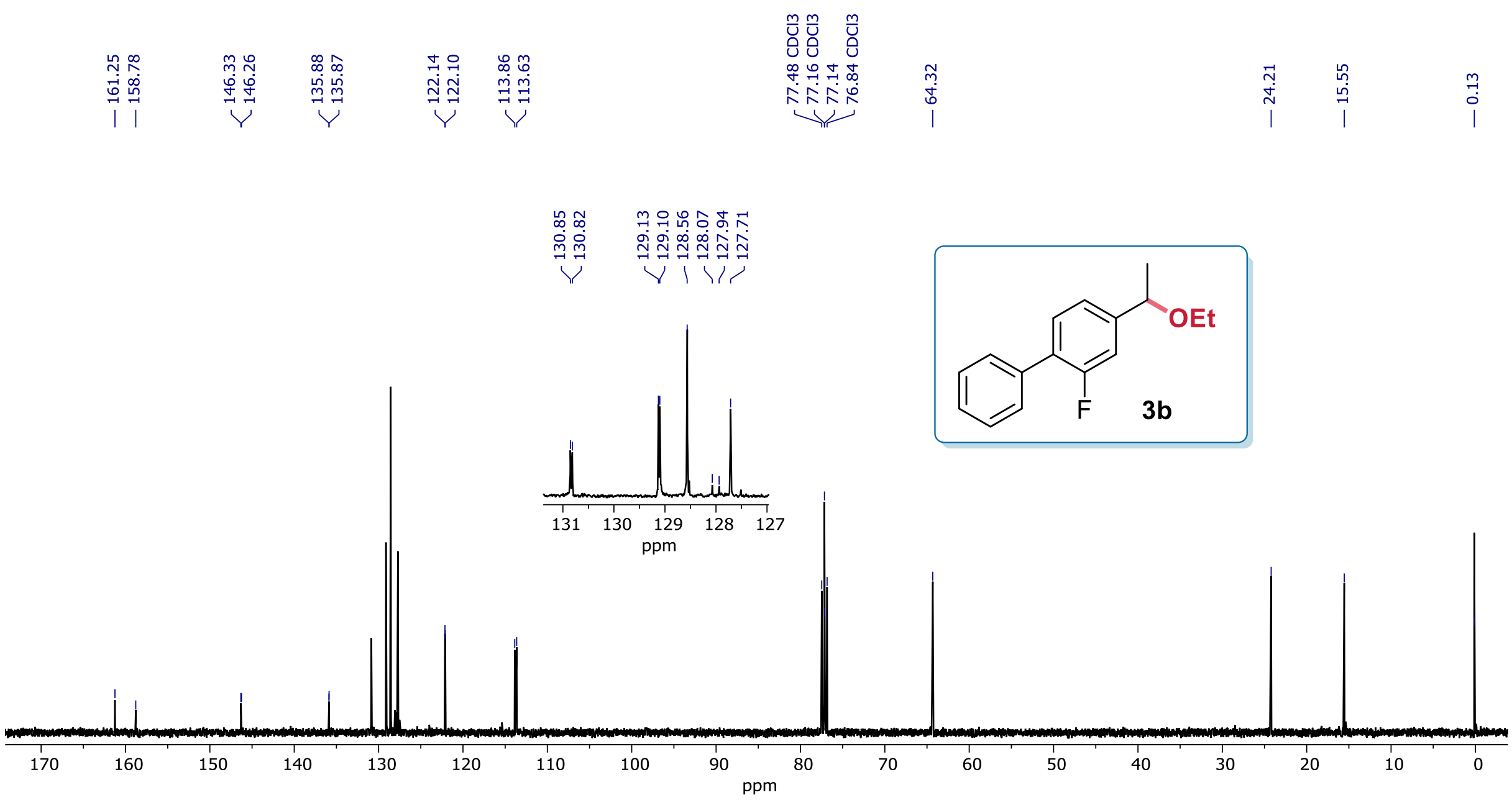

Figure. S40 $^{13}C\{^{1}H\}$ NMR spectrum of **3b** in $CDCl_3$ (101 MHz)

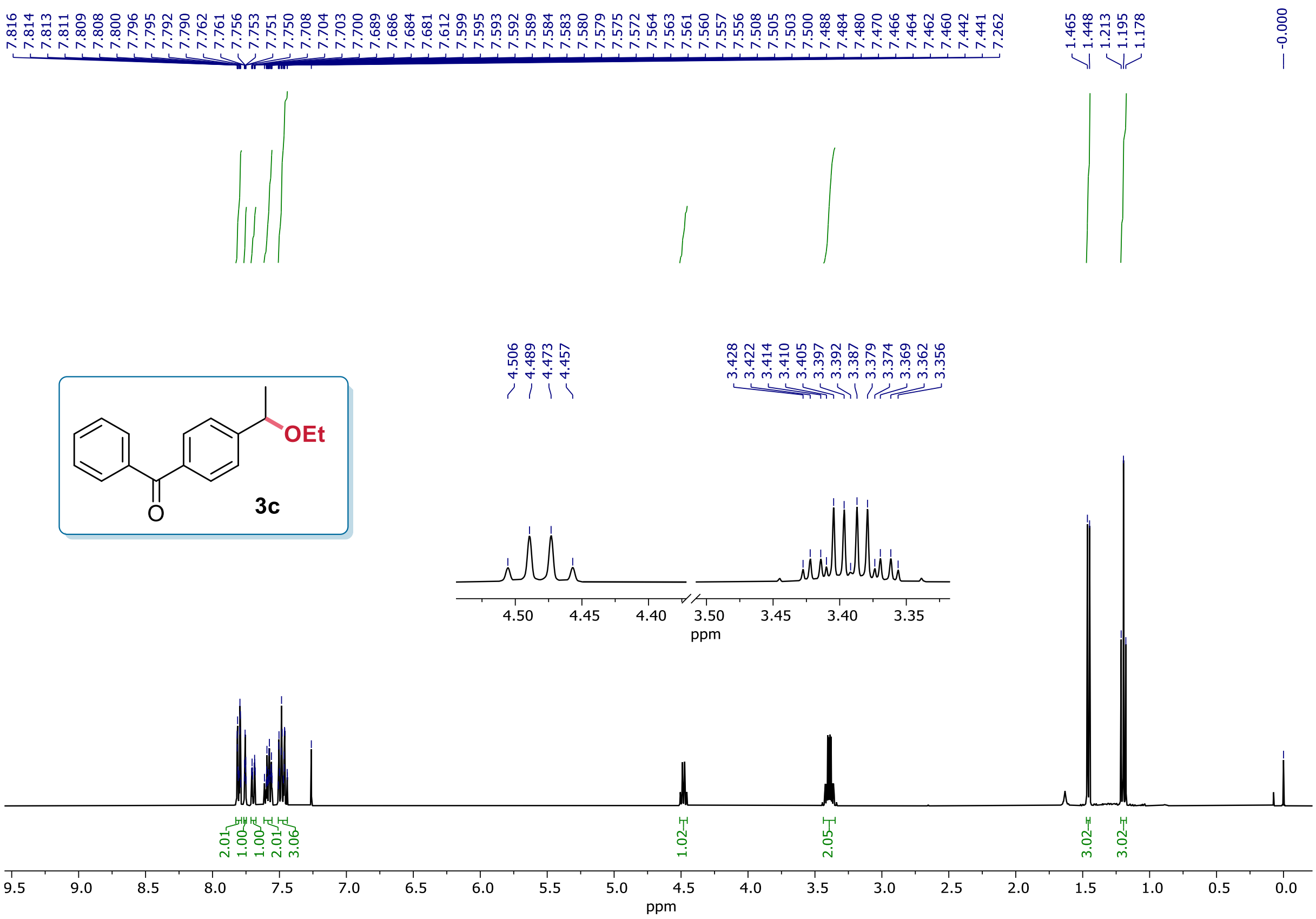

Figure. S41 ^{1}H NMR spectrum of **3c** in $CDCl_3$ (400 MHz)

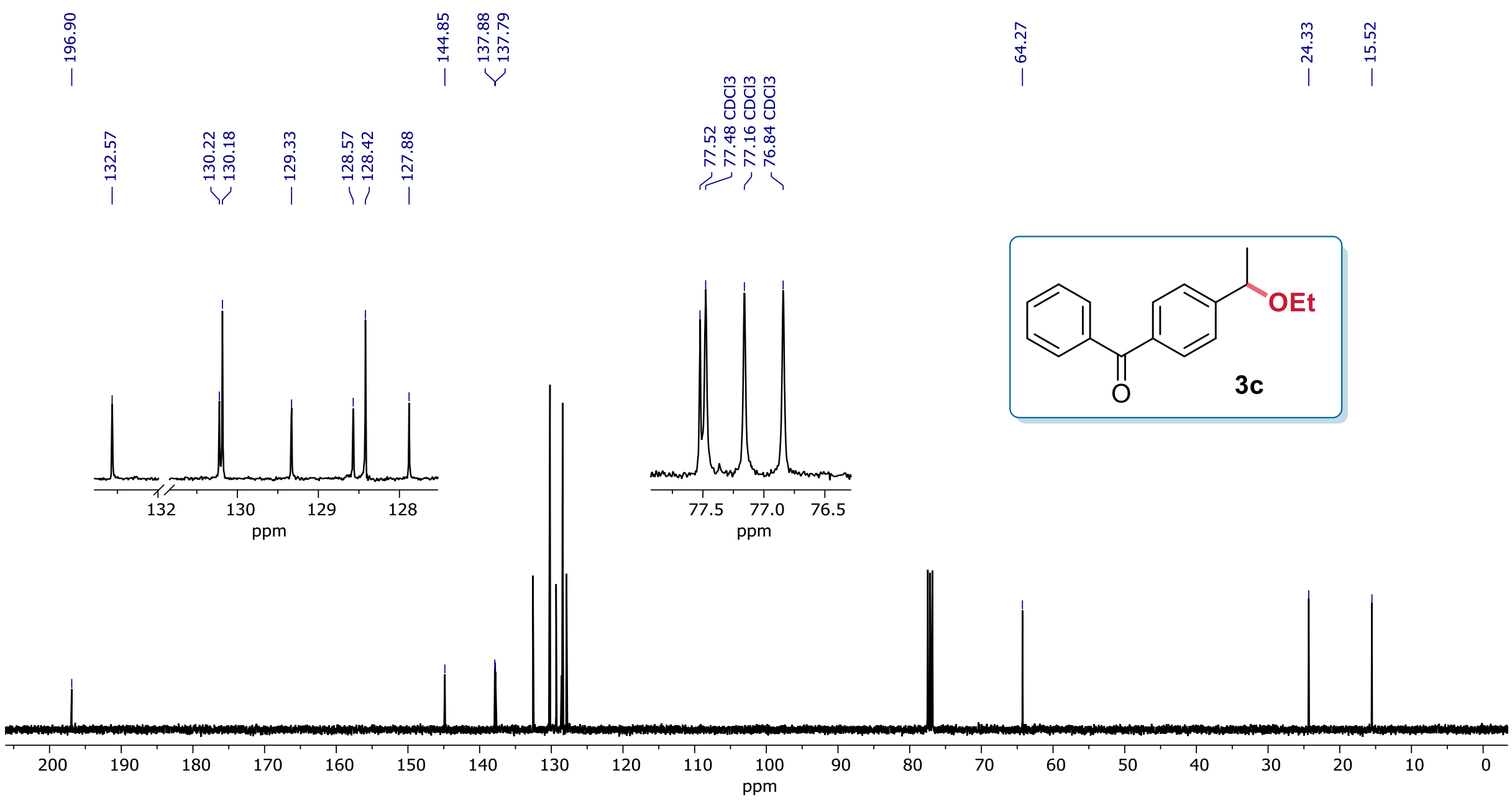

Figure. S42 $^{13}C\{^{1}H\}$ NMR spectrum of **3c** in $CDCl_3$ (101 MHz)

5. ^{1}H NMR spectra of the selected reaction mixtures

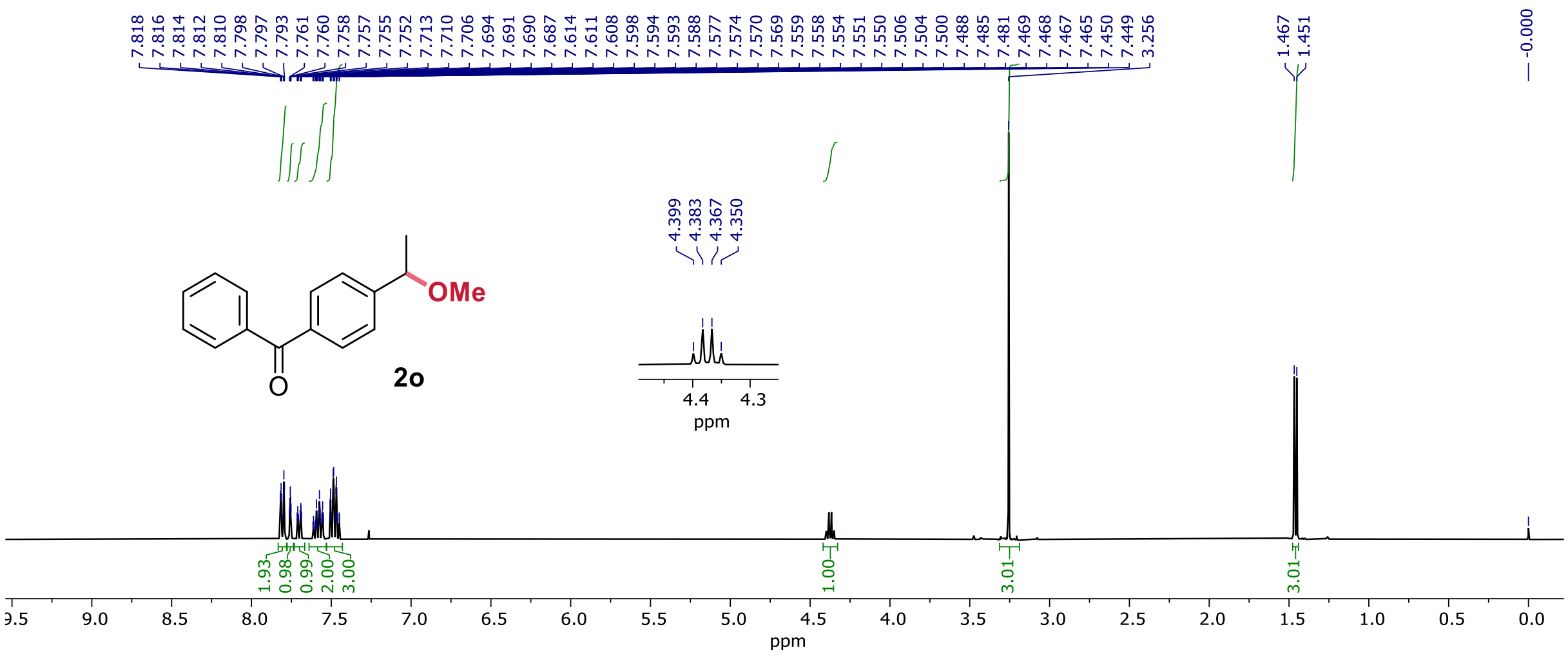

Figure. S43 ^{1}H NMR spectrum of the isolated compound **2o** in $CDCl_3$ (400 MHz)

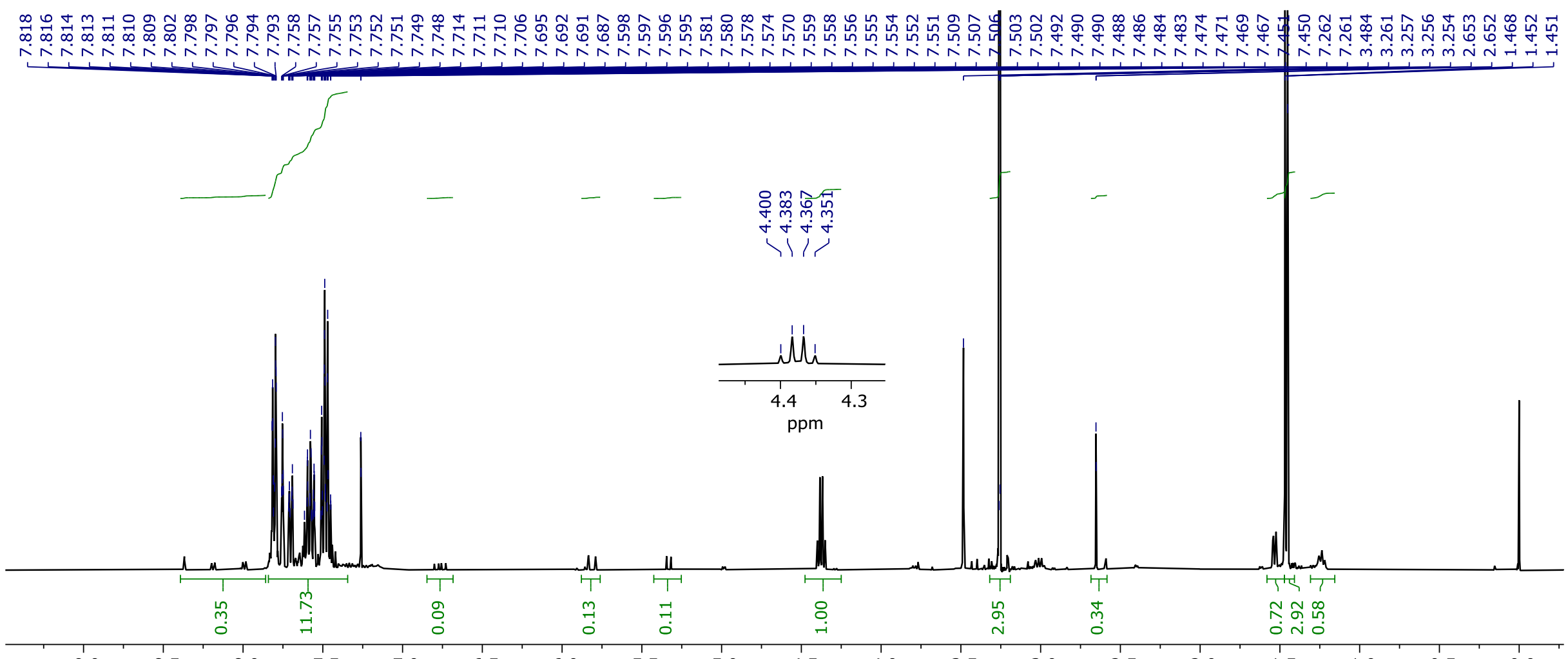

Figure. S44 ^{1}H NMR spectrum of the reaction mixture obtained in the synthesis of compound **2o** in $CDCl_3$ (400 MHz)

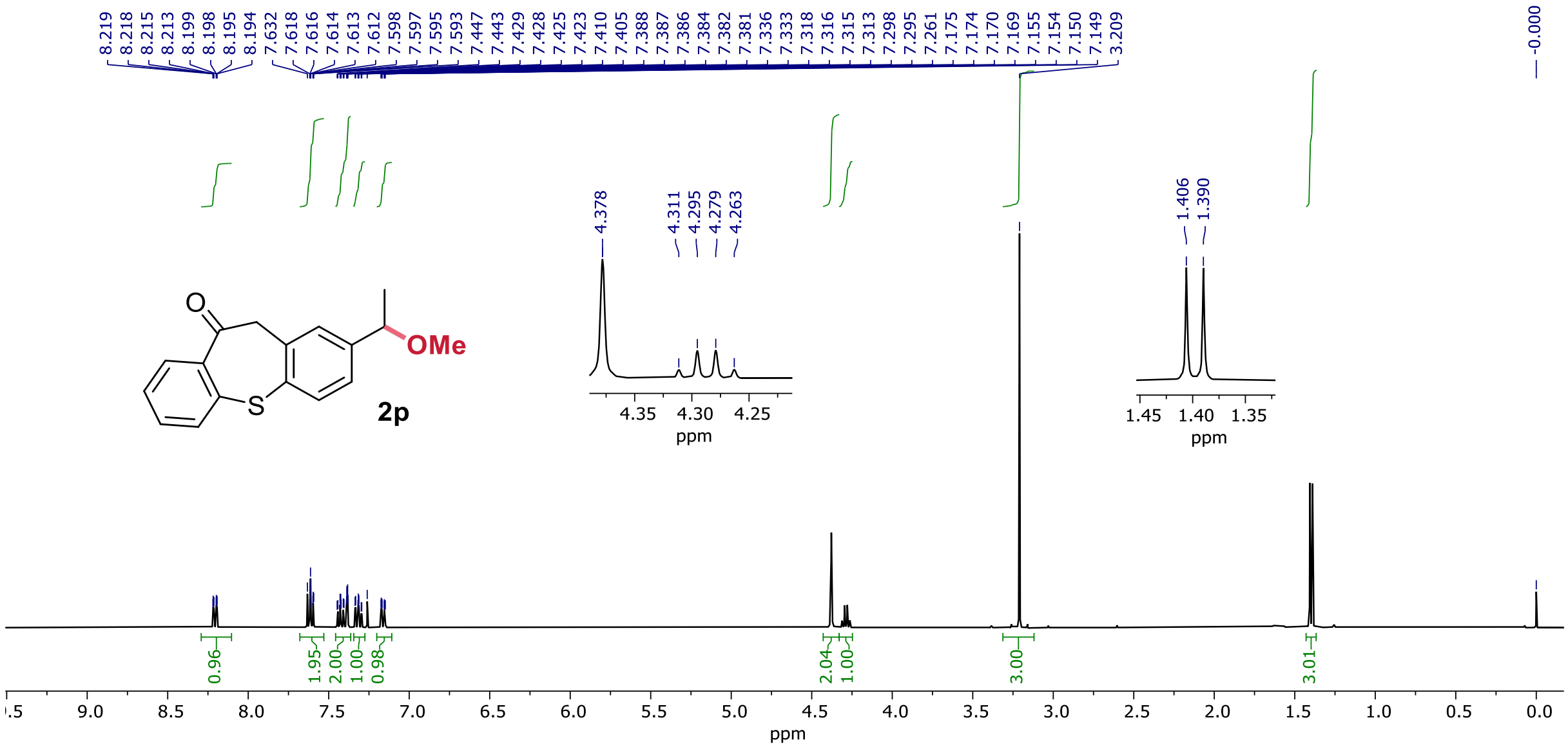

Figure. S45 ¹H NMR spectrum of the isolated compound **2p** in $CDCl_3$ (400 MHz)

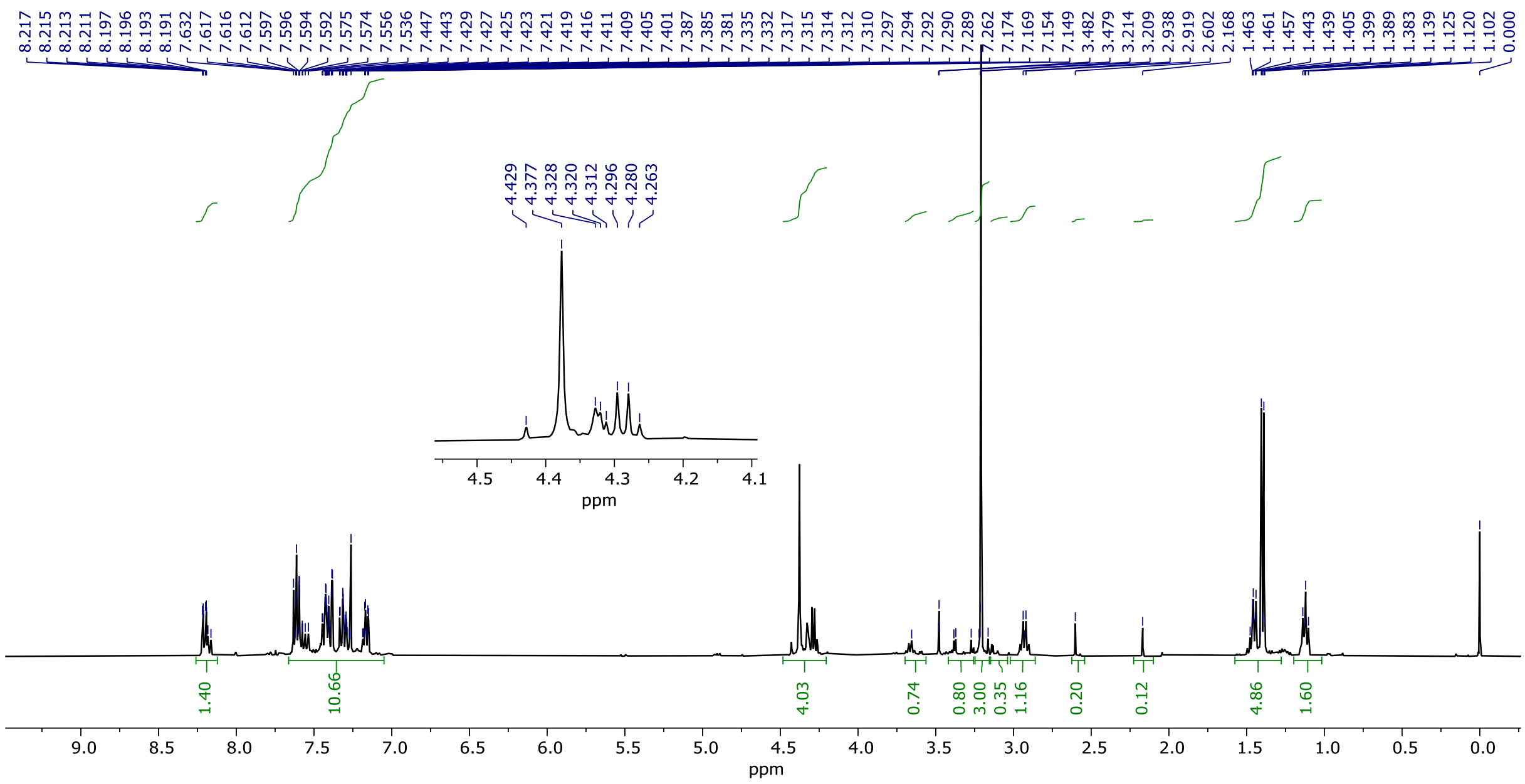

Figure. S46 ¹H NMR spectrum of the reaction mixture obtained in the synthesis of compound **2p** in $CDCl_3$ (400 MHz)

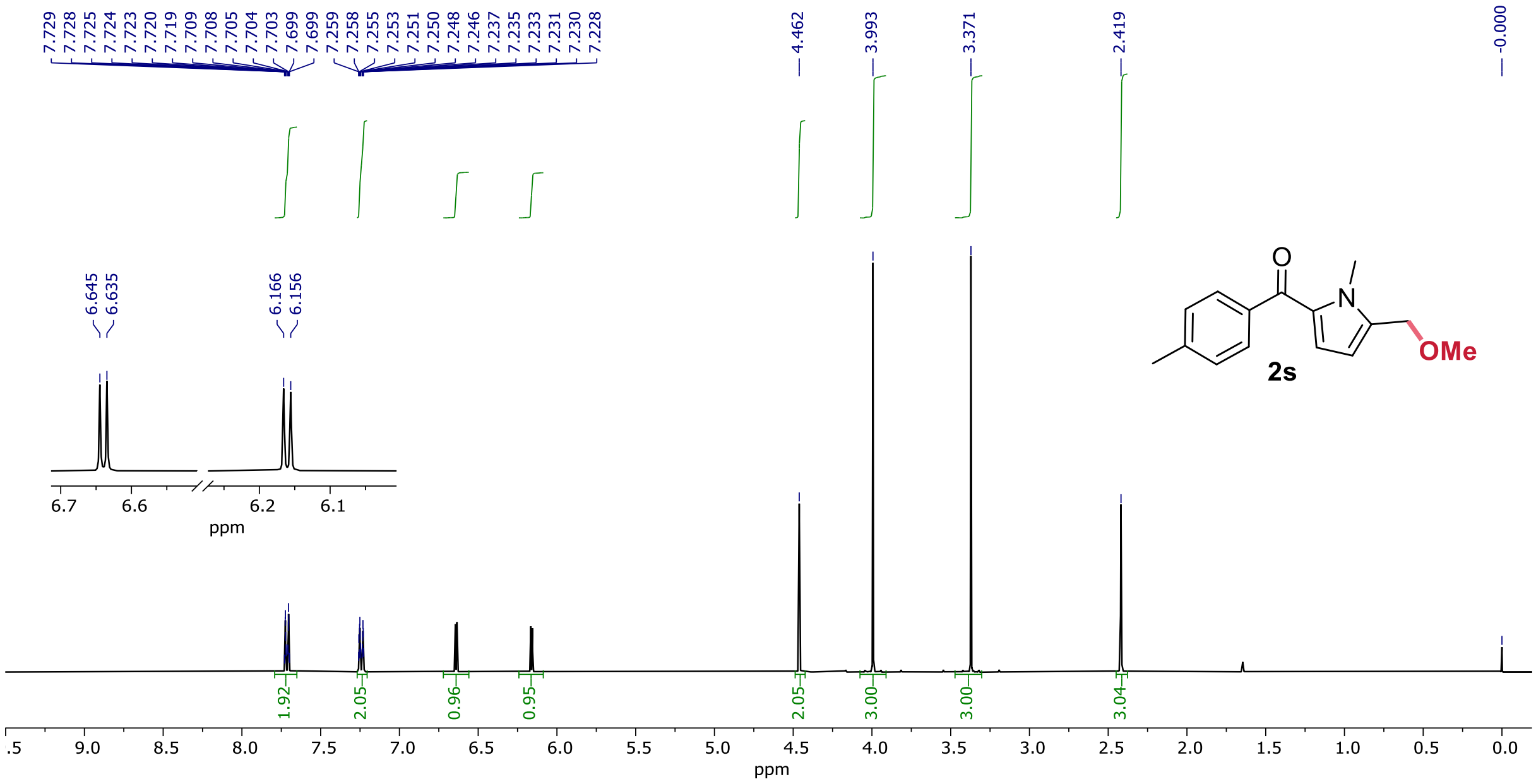

Figure. S47 ^{1}H NMR spectrum of the isolated compound **2s** in $CDCl_3$ (400 MHz)

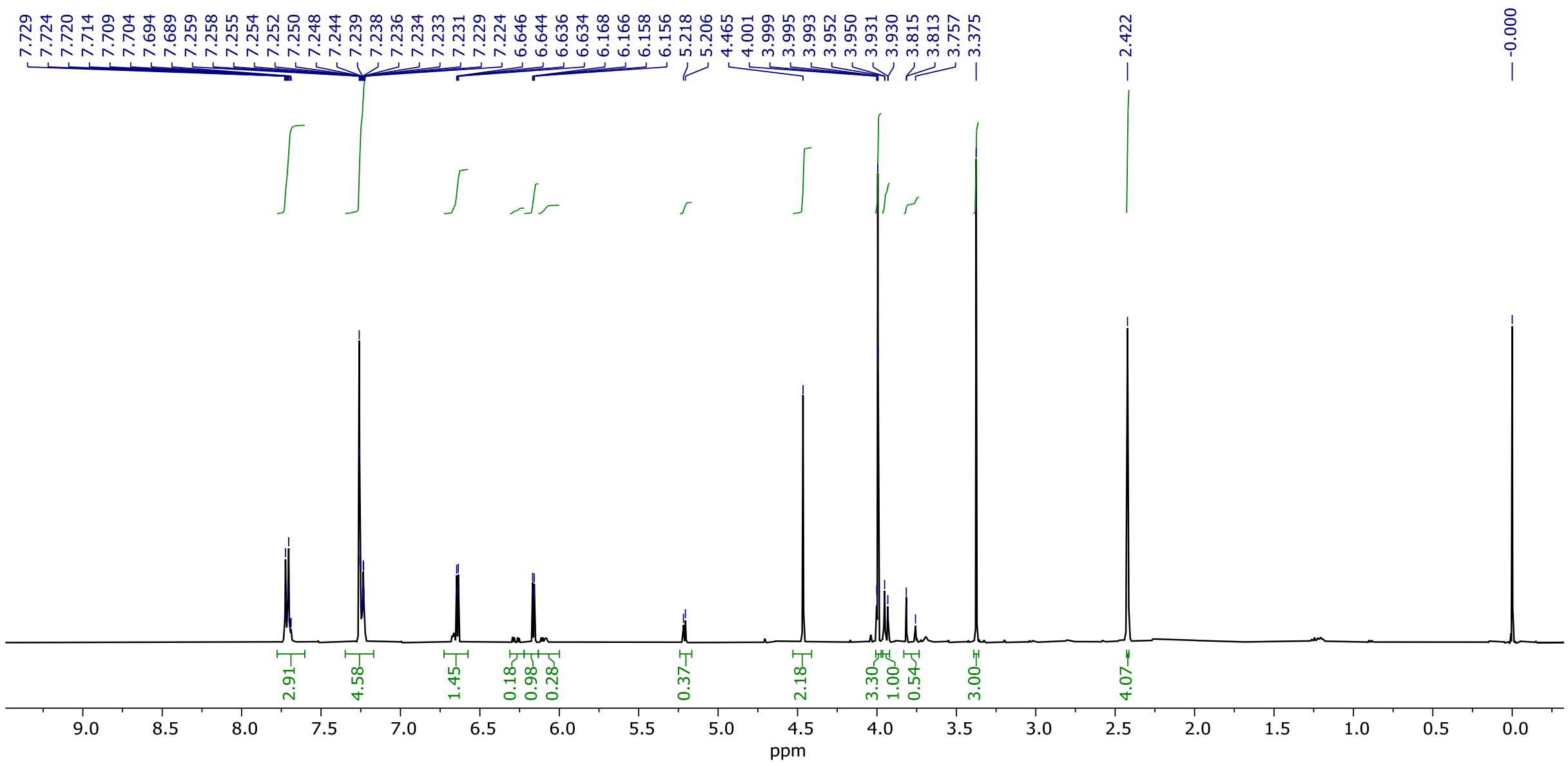

Figure. S48 ^{1}H NMR spectrum of the reaction mixture obtained in the synthesis of compound **2s** in $CDCl_3$ (400 MHz)

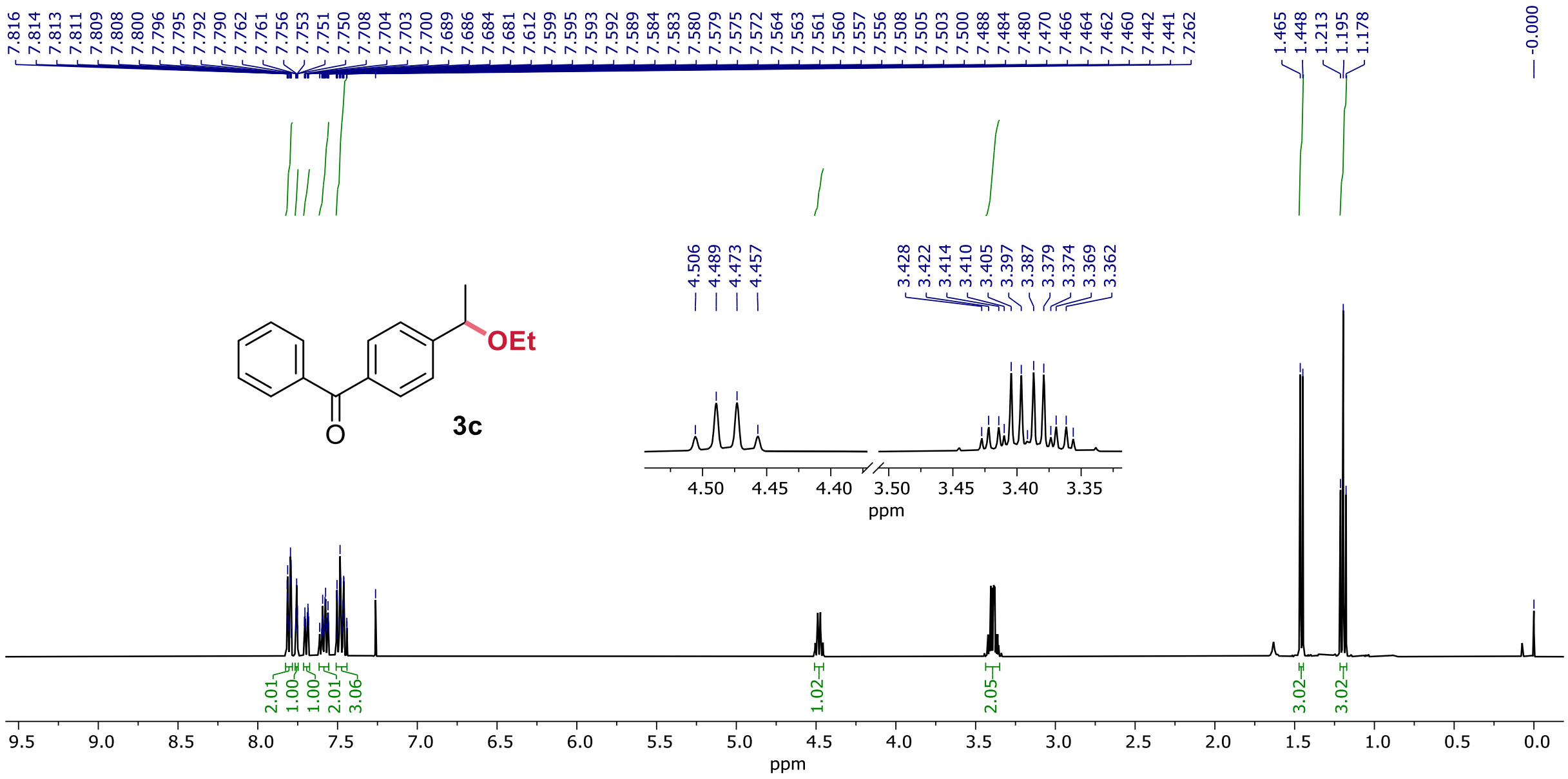

Figure. S49 ^{1}H NMR spectrum of the isolated compound **3c** in $CDCl_3$ (400 MHz)

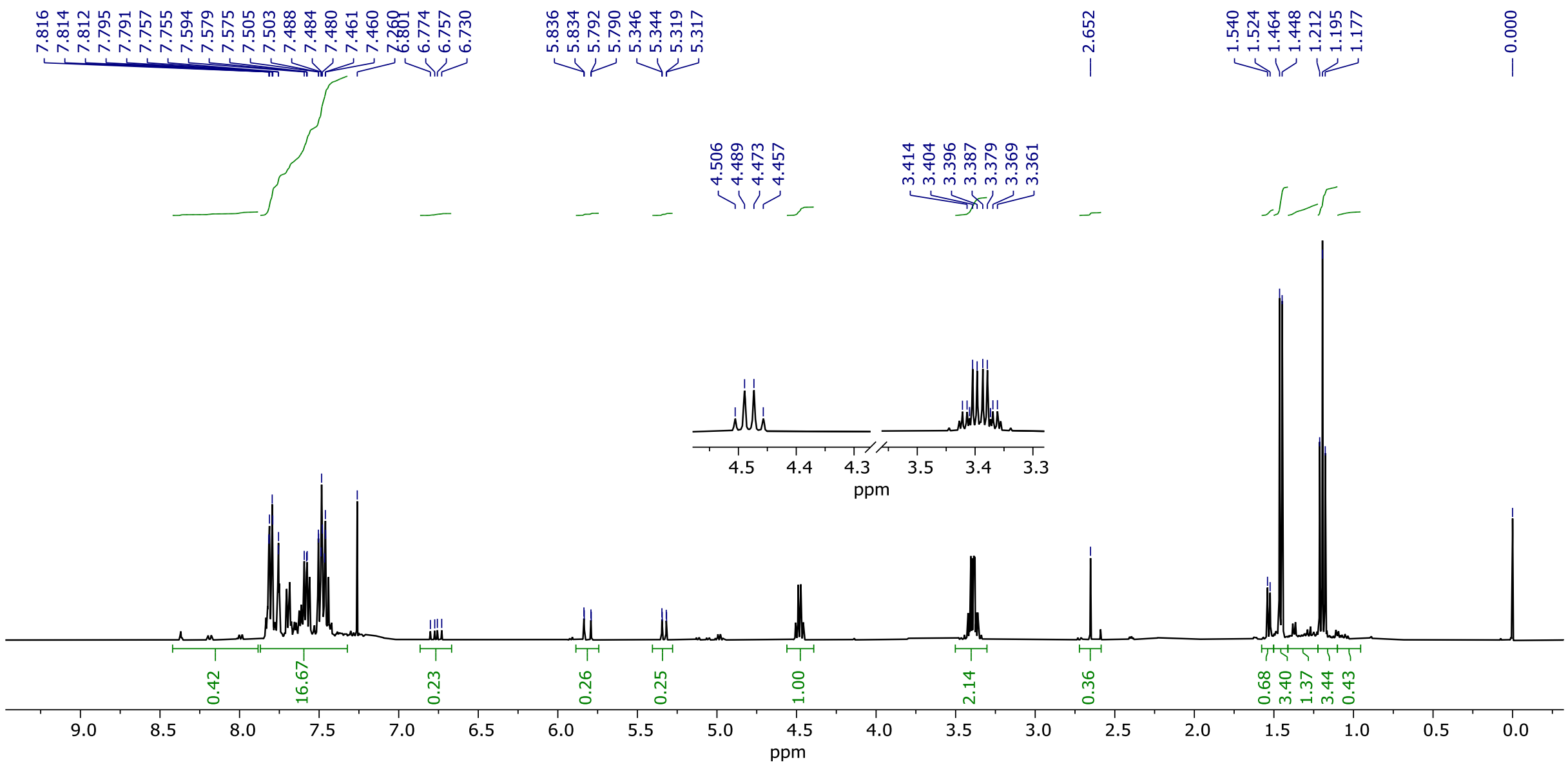

Figure. S50 ^{1}H NMR spectrum of the reaction mixture obtained in the synthesis of compound **3c** in $CDCl_3$ (400 MHz)

6. References

1. Ghosh, A.; Parida, V.K.; von Münchow, T.; Ackermann, L.; Banerjee, D. Highly-Selective Electrochemical Decarboxylative Late-Stage Functionalization of Amino Acids, *Chemistry – A European Journal*, **2025**, *31*, e202501287.

2. Yu, P.; Huang, X.; Wang, D.; Yi, H.; Song, C.; Li, J. Electrochemical Decarboxylative Cross-Coupling with Nucleophiles, *Chemistry – A European Journal*, **2024**, *30*, e202402124.

3. Li, Q.Y.; Gockel, S.N.; Lutovsky, G.A.; DeGlopper, K.S.; Baldwin, N.J.; Bundesmann, M.W.; Tucker, J.W.; Bagley, S.W.; Yoon, T.P. Decarboxylative cross-nucleophile coupling via ligand-to-metal charge transfer photoexcitation of Cu(ii) carboxylates, *Nature Chemistry*, **2022**, *14*, 94-99.

4. Ke, F.; Li, Z.; Xiang H.; Zhou, X. Catalytic hydroalkoxylation of alkenes by iron(III) catalyst, *Tetrahedron Letters*, **2011**, *52*, 318-320.

5. Ayub, R.; Papadakis, R.; Jorner, K.; Zietz B.; Ottosson, H. Cyclopropyl Group: An Excited-State Aromaticity Indicator?, *Chemistry – A European Journal*, **2017**, *23*, 13684-13695.

6. Azzena, U.; Pilo, L.; Sechi, A. Metalation of arylmethyl alkyl ethers, *Tetrahedron*, **1998**, *54*, 12389-12398.

7. Shi, M.; Okamoto, Y.; Takamuku, S. Photolysis of 1,1,1-triarylalkane. A new photochemical carbene generation process, *J. Org. Chem.*, **1990**, *55*, 3821-3824.

8. Guo, P.; Song, X.; Huang, B.; Zhang, R.; Zhao, J. Photoinduced Low-Valent Zirconium Catalysis for Cross-Electrophile Coupling of Ethers, *Angew. Chem. Int. Ed.*, **2024**, *63*, e202405449.

9. Boscá, F.; Miranda, M.A.; Vargas, F. Photochemistry of tiaprofenic acid, a nonsteroidal anti-inflammatory drug with phototoxic side effects, *Journal of Pharmaceutical Sciences*, **1992**, *81*, 181-182.

10. Koyanagi, T.; Herath, A.; Chong, A.; Ratnikov, M.; Valiere, A.; Chang, J.; Molteni, V.; Loren, J. One-Pot Electrochemical Nickel-Catalyzed Decarboxylative Sp2–Sp3 Cross-Coupling, *Org. Lett.*, **2019**, *21*, 816-820.

11. Lutovsky, G. A.; Gockel, S. N.; Bundesmann, M. W.; Bagley, S. W.; Yoon, T. P., Iron-mediated modular decarboxylative cross-nucleophile coupling. *Chem* **2023,** *9*, 1610-1621.
